# Antigen-derived peptides engage the ER stress sensor IRE1α to curb dendritic cell cross-presentation

**DOI:** 10.1083/jcb.202111068

**Published:** 2022-04-21

**Authors:** Ofer Guttman, Adrien Le Thomas, Scot Marsters, David A. Lawrence, Lauren Gutgesell, Iratxe Zuazo-Gaztelu, Jonathan M. Harnoss, Simone M. Haag, Aditya Murthy, Geraldine Strasser, Zora Modrusan, Thomas Wu, Ira Mellman, Avi Ashkenazi

**Affiliations:** 1 Departments of Cancer Immunology, Genentech, South San Francisco, CA; 2 Departments of Microchemistry, Proteomics and Lipidomics, Genentech, South San Francisco, CA; 3 Departments of Oncology Bioinformatics, Genentech, South San Francisco, CA

## Abstract

Dendritic cells (DCs) promote adaptive immunity by cross-presenting antigen-based epitopes to CD8^+^ T cells. DCs process internalized protein antigens into peptides that enter the endoplasmic reticulum (ER), bind to major histocompatibility type I (MHC-I) protein complexes, and are transported to the cell surface for cross-presentation. DCs can exhibit activation of the ER stress sensor IRE1α without ER stress, but the underlying mechanism remains obscure. Here, we show that antigen-derived hydrophobic peptides can directly engage ER-resident IRE1α, masquerading as unfolded proteins. IRE1α activation depletes MHC-I heavy-chain mRNAs through regulated IRE1α-dependent decay (RIDD), curtailing antigen cross-presentation. In tumor-bearing mice, IRE1α disruption increased MHC-I expression on tumor-infiltrating DCs and enhanced recruitment and activation of CD8^+^ T cells. Moreover, IRE1α inhibition synergized with anti–PD-L1 antibody treatment to cause tumor regression. Our findings identify an unexpected cell-biological mechanism of antigen-driven IRE1α activation in DCs, revealing translational potential for cancer immunotherapy.

## Teaser

Antigen cross-presenting dendritic cells (DCs) activate the ER stress sensor IRE1α without ER stress. Guttman et al. show that antigen-derived ER-imported peptides can directly engage IRE1α. Regulated IRE1α-dependent MHC-I mRNA decay curbs DC cross-presentation, while IRE1α inhibition augments antitumor immunity.

## Introduction

The ER mediates 3D folding of newly synthesized proteins that are destined for plasma membrane insertion or extracellular secretion. Elevated demand for protein folding causes ER stress and triggers the unfolded protein response (UPR), which drives ER adaptation to restore homeostasis (Hetz, 2012; Walter and Ron, 2011; Wang and Kaufman, 2016). The mammalian UPR comprises three branches, controlled by corresponding ER-transmembrane proteins: IRE1α, PERK, and ATF6. IRE1α senses ER stress mainly through its ER-lumenal domain and transmits intracellular signals via a cytoplasmic kinase-endoribonuclease (RNase) module (Cox et al., 1993; Lee et al., 2008). IRE1α detects misfolded proteins through indirect and direct mechanisms: Indirect engagement involves unfolded-protein binding to the ER chaperone BiP/GRP78, which otherwise keeps IRE1α in check (Amin-Wetzel et al., 2017; Bertolotti et al., 2000). Direct engagement involves unfolded-protein binding to the lumenal domain of IRE1α through exposed hydrophobic regions that are otherwise buried within correctly folded proteins (Gardner and Walter, 2011; Karagoz et al., 2017). IRE1α activation entails molecular changes that include homo-oligomerization, kinase trans-autophosphorylation, and consequent RNase engagement (Korennykh et al., 2009; Tirasophon et al., 1998).

The IRE1α RNase performs two central functions: (1) activation of the transcription factor X-box protein 1 spliced (XBP1s; Hetz, 2012; Walter and Ron, 2011; Wang and Kaufman, 2016); (2) depletion of select mRNAs through the process of regulated IRE1α-dependent decay (RIDD; Hollien et al., 2009; Hollien and Weissman, 2006). XBP1s induces multiple genes that support ER-mediated protein folding, as well as ER-associated degradation (ERAD) of misfolded proteins (Acosta-Alvear et al., 2007; Brodsky, 2012; Lee et al., 2003; Smith et al., 2011; Travers et al., 2000). RIDD on the other hand depletes specific ER-targeted mRNAs to abate ER load (Hollien, 2013; Hollien et al., 2009; Hollien and Weissman, 2006; Lhomond et al., 2018; Maurel et al., 2014). RIDD also regulates additional cellular functions by degrading mRNAs encoding proteins that control triglyceride and cholesterol metabolism (So et al., 2012), apoptosis (Chang et al., 2018; Lam et al., 2020; Lu et al., 2014a), autophagy (Bae et al., 2019), antibody production (Tang et al., 2018), and DNA repair (Dufey et al., 2020; Tang et al., 2018).

To process XBP1 mRNA for splicing, the IRE1α RNase recognizes two stem-loop structures located 26 nucleotides apart; each loop contains the consensus sequence endomotif CNGCAGC (Calfon et al., 2002; Shen et al., 2001; Yoshida et al., 2001). IRE1α cleaves between the guanine (G) at position 3 and the cytosine (C) at position 4 (Hooks and Griffiths-Jones, 2011; Peschek et al., 2015). Subsequently, RtcB ligates the resulting 5′ and 3′ RNA exons to produce XBP1s (Jurkin et al., 2014; Kosmaczewski et al., 2014; Lu et al., 2014b; Peschek et al., 2015). In mammals, RIDD often requires an XBP1-like endomotif, CNGCAGN, within a predicted stem-loop structure (Hollien, 2013; Moore and Hollien, 2015; Moore et al., 2013; Oikawa et al., 2010). IRE1α can also cleave certain mRNAs through a more promiscuous process dubbed RIDDLE (for RIDD lacking endomotif; Le Thomas et al., 2021). Experimental XBP1s disruption increases IRE1α autophosphorylation and augments RIDD (Chen et al., 2014; Lu et al., 2014a; Osorio et al., 2014).

Dendritic cells (DCs) comprise a unique myeloid cell subset that plays a crucial role in antigen presentation during the development and elaboration of adaptive immunity (Mellman and Steinman, 2001; Steinman, 2007). Certain DC lineages mediate the specialized process of antigen cross-presentation, which initiates cytotoxic CD8^+^ T cell responses (Gatti and Pierre, 2003; Mellman and Steinman, 2001; Palucka et al., 2010). DCs are much more proficient when compared with other cell types in acquiring antigens from tissue microenvironments through endocytosis of soluble proteins or phagocytosis of cell fragments and corpses (Chen et al., 2001; Sallusto et al., 1995). During exposure to a pulse of protein antigen, DCs internalize the polypeptide, which reaches the cytoplasm and undergoes proteasomal processing into shorter peptides (Alloatti et al., 2016). Subsequently, the transporter associated with antigen processing (TAP), which resides in the ER membrane, enables importation of the peptides into the ER lumen. Within the ER, the peptides are bound through chaperone-aided events to major histocompatibility type I (MHC-I) protein complexes, which are composed of heavy and light polypeptide chains (Jhunjhunwala et al., 2021; Thomas and Tampe, 2017). The peptide–MHC complexes traffic to the DC surface, where the epitopes are cross-presented, engaging cognate T cell receptors (TCRs) on juxtaposing T cells. In cancer, both intrinsic and therapeutic mechanisms require efficient DC-mediated cross-presentation of tumor antigens to CD8^+^ T cells to achieve effective anti-tumor immunity (Barber et al., 2006; Barry et al., 2018; Chen and Mellman, 2013; Garris et al., 2018; Jansen et al., 2019; Spranger et al., 2016). However, few treatment strategies are currently available to directly modulate DC cross-presentation (Palucka and Banchereau, 2012; Timmerman et al., 2002).

DCs can exhibit IRE1α activation in the absence of canonical ER stress (Iwakoshi et al., 2007; Osorio et al., 2014; Tavernier et al., 2017). *XBP1* gene knockout (KO) studies indicate divergent effects on antigen cross-presentation in different subtypes of DCs. In CD8α^+^ DCs, *XBP1* KO led to a hyper-activated RIDD phenotype, which disrupted T cell activation by depleting mRNAs encoding specific components of the cross-presentation machinery, i.e., Lamp-1 and TAP binding protein (TAPBP; Iwakoshi et al., 2007; Osorio et al., 2014; Tavernier et al., 2017). In contrast, in tumor-associated DCs, *XBP1* KO led to changes in lipid metabolism, which improved cross-presentation of tumor antigens and consequent anti-tumor T cell activity (Cubillos-Ruiz et al., 2015). In conventional (c) DC1 subpopulations residing in the lung, *XBP1* KO together with partial *IRE1α* gene disruption reduced DC viability (Tavernier et al., 2017). Pulsing of bone marrow–derived DCs (BMDCs) with melanoma cell lysates as a source of antigens upregulated XBP1s without affecting RIDD, while IRE1α inhibition attenuated cross-presentation to CD8^+^ T cells (Medel et al., 2018). While these studies implicate IRE1α in the regulation of DCs, the cell-biological mechanisms that promote IRE1α activation in these cells in the absence of canonical ER stress remain poorly defined.

In the present study, we reveal that antigen-derived peptides can engage IRE1α in antigen-pulsed DCs, mimicking the action of misfolded proteins. Antigen-induced IRE1α activation curtailed cross-presentation through RIDD-mediated depletion of MHC-I heavy-chain mRNAs. Blocking IRE1α function in tumor-bearing mice using a highly specific kinase-based IRE1α inhibitor upregulated MHC-I levels on DCs, enhanced tumor recruitment and activation of CD8^+^ T cells, and cooperated with anti–programmed death ligand 1 (PD-L1) immune-checkpoint disruption to cause tumor regression. Our findings identify an unexpected mechanism of IRE1α activation in DCs, with potential implications for cancer immunotherapy.

## Results

### Antigen pulsing of BMDCs activates IRE1α

**Figure 1.**
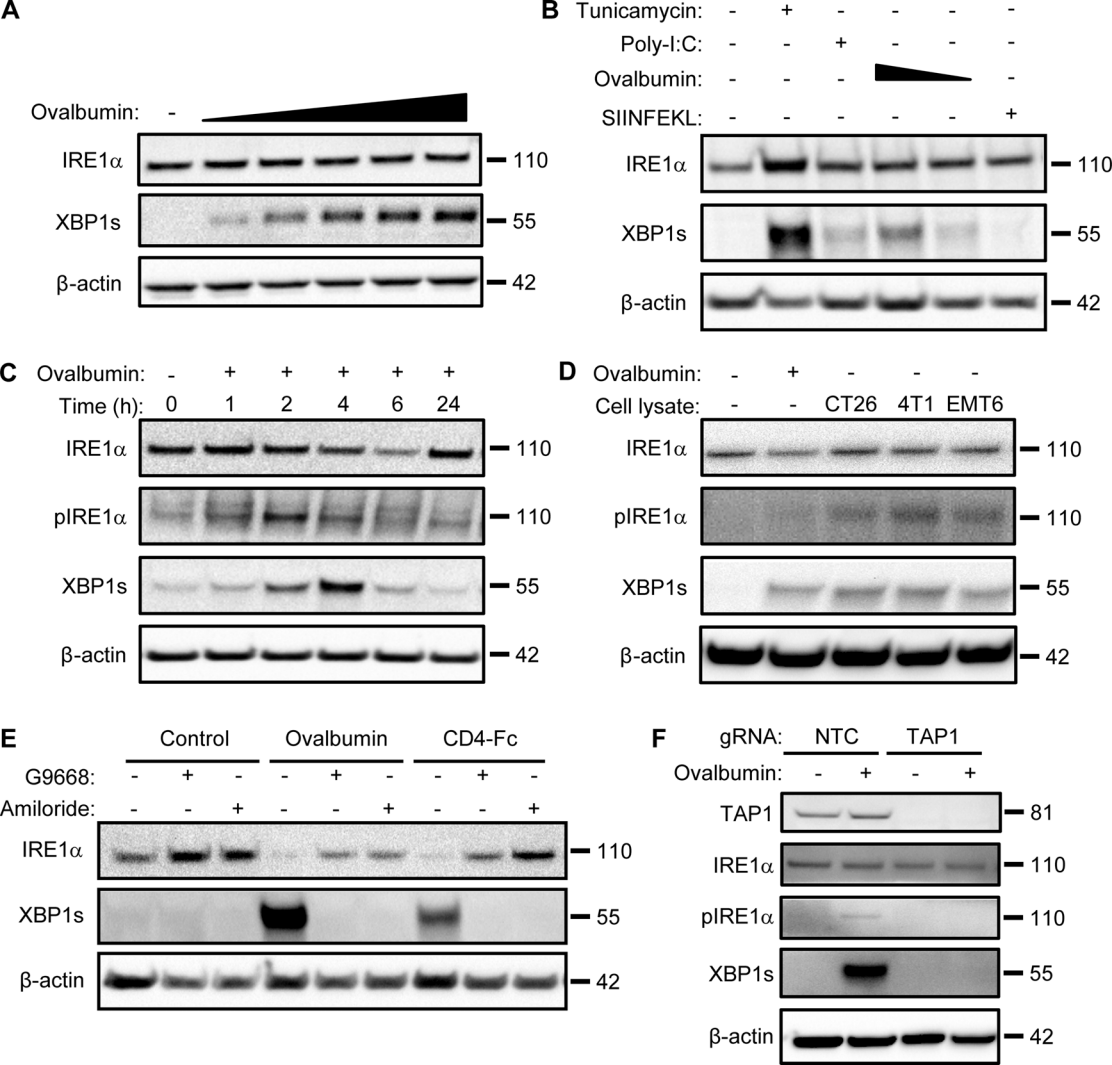
**Antigen pulsing of BMDCs activates IRE1α. (A)** BMDCs were pulsed with ovalbumin (starting at 62.5 μg/ml and sequentially doubled) and analyzed by immunoblot (IB) for the indicated markers. **(B)** BMDCs were pulsed with ovalbumin (starting at 500 μg/ml and sequentially halved) or SIINFEKL (1 μM), or stimulated with tunicamycin (1 μg/ml) or poly-I:C (25 μg/ml) for 4 h, and analyzed by IB. **(C)** BMDCs were pulsed with ovalbumin (500 μg/ml) for indicated time points and analyzed by IB. **(D)** BMDCs were pulsed for 4 h with ovalbumin or lysates derived from the indicated cell lines (500 μg/ml protein) and analyzed by IB. **(E)** BMDCs were pulsed for 4 h with ovalbumin or human soluble CD4-Fc fusion protein (both at 500 μg/ml), combined with DMSO or G9668 (3 μM) or amiloride (10 μM), and analyzed by IB. **(F)** Upon removal from bone marrow, total bone marrow cells were transfected with non-targeting control (NTC) or TAP1-targeting gRNAs, along with CRISPR/Cas9 delivery constructs. Cells were then subjected to standard BMDC-differentiation protocol, pulsed with ovalbumin (500 μg/ml) for 4 h, and analyzed by IB. All IB images are representative of at least two similar experiments, and molecular weights represent kD. Source data are available for this figure: SourceData F1.

**Figure S1.**
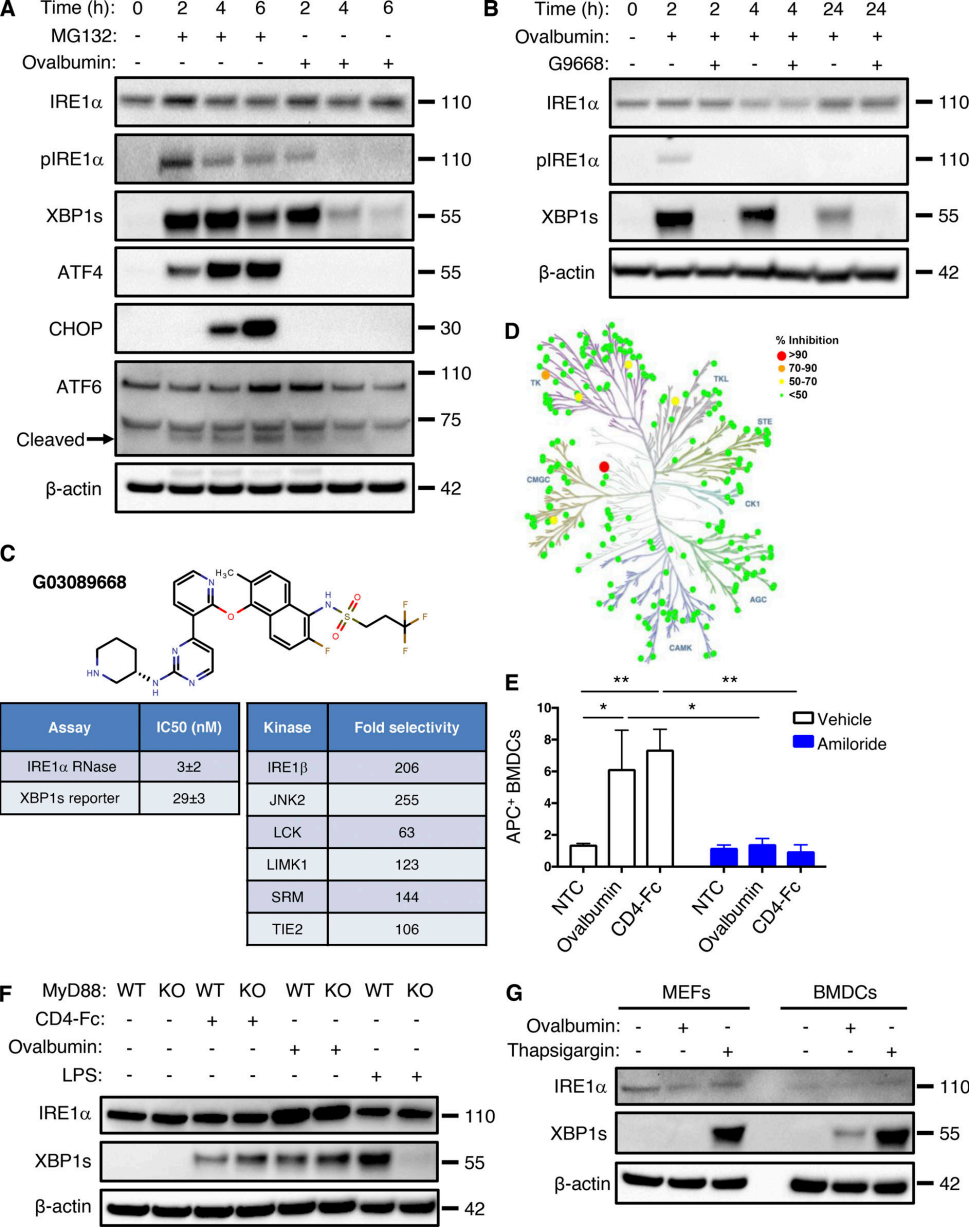
**Antigen pulsing of BMDCs activates IRE1α. (A)** BMDCs were treated with MG132 (5 μM) or ovalbumin (500 μg/ml) for indicated time points and analyzed by IB. **(B)** BMDCs were pulsed with ovalbumin (500 μg/ml) and G9668 (3 μM) for indicated time periods and analyzed by IB. **(C)** Molecular structure, potency, and kinase selectivity of G9668. **(D)** Schematic representation of G9668 interaction with 220 kinases at 1 μM. Size and color of circles represent interaction strength. Analysis was conducted by KinomeScan. **(E)** BMDCs were pulsed for 4 h with APC-tagged ovalbumin or soluble CD4-Fc protein (500 μg/ml) with amiloride (10 μM); uptake of protein was assayed by flow cytometry (*n* = 3). **(F)** WT or MyD88 KO Flt3L-derived BMDCs were pulsed with ovalbumin (500 μg/ml) or soluble CD4-Fc protein (500 μg/ml), or treated with LPS (10 μg/ml) for 4 h and analyzed by IB. **(G)** MEFs and BMDCs were pulsed with ovalbumin or treated with Tg (100 nM) for 4 h and analyzed by IB. Images in A, B, F, and G represent at least two similar experiments, and molecular weights represent kD; E bar graphs represent mean ± SD from three independent biological repeats. Analysis for E was performed using unpaired, two-tailed *t* test; *, P ≤ 0.05; **, P ≤ 0.01. Source data are available for this figure: SourceData FS1.

Although ex vivo maturation of bone marrow–derived myeloid cells with granulocyte-macrophage colony-stimulating factor (GM-CSF) yields mainly macrophage-like characteristics (Helft et al., 2015), the addition of IL-4 on top of GM-CSF during maturation strongly favors differentiation toward the DC phenotype (Jin and Sprent, 2018). Besides greater efficiency, an important advantage of this latter maturation approach is that it produces DCs that are relatively quiescent, as compared to direct isolation of primary DCs from spleen or other tissues, which typically yields already stimulated cells (Schlecht et al., 2006). Therefore, to explore potential mechanisms of IRE1α activation in DCs, we first generated mouse BMDCs through ex vivo maturation with GM-CSF plus IL-4 and pulsed them with the classical protein antigen ovalbumin. To cross-present ovalbumin, DCs must internalize the pulsed protein and process it intracellularly; in contrast, DCs can directly present the ovalbumin-derived octapeptide SIINFEKL based on its ability to displace antigens already bound to MHC-I complexes at the cell surface (Alloatti et al., 2016). Ovalbumin pulsing of BMDCs induced concentration- and time-dependent activation of IRE1α, evident by increased protein levels of XBP1s (Fig. 1, A and B). The toll-like receptor (TLR) agonist polyinosinic:polycytidylic acid (poly-I:C) was previously shown to activate IRE1α in leukocytes (Martinon et al., 2010). Similar to poly-I:C, ovalbumin pulsing induced much weaker IRE1α activation than did the potent pharmacological ER-stressor tunicamycin (Fig. 1 B). In contrast to ovalbumin, SIINFEKL had little effect on IRE1α activity (Fig. 1 B), suggesting a requirement for intracellular events. In response to ER-stress induction by the proteasome inhibitor MG132, all three major UPR branches showed robust activation in BMDCs (Fig. S1 A); in contrast, in response to ovalbumin pulsing of BMDCs, only IRE1α exhibited substantial activation, whereas PERK did not show a measurable response, as judged by its downstream mediators ATF4 and CHOP, while ATF6 displayed relatively minimal activation as indicated by its proteolytic processing (Fig. S1 A). To verify a bona fide IRE1α activation during ovalbumin pulsing, we added the novel and highly selective kinase-based small molecule IRE1α inhibitor, G03089668 (G9668; Fig. S1, B–D; Ghosh et al., 2014; Harnoss et al., 2020; Harnoss et al., 2019; Harrington et al., 2015), which completely blocked IRE1α phosphorylation and XBP1s induction. During a 4-h pulse of ovalbumin, IRE1α phosphorylation peaked at 2 h while XBP1s protein peaked at 2–4 h (Figs. 1 C and S1 A). Taken together, these results indicate a rapid, yet transient, stimulation of IRE1α activity in BMDCs in response to ovalbumin pulsing. Similarly, BMDC pulsing with lysates derived from CT26, 4T1, and EMT6 tumor cells (see below) also activated IRE1α (Fig. 1 D). Moreover, pulsing with a different, highly purified recombinant protein, i.e., clinical-grade soluble CD4-Fc, also led to IRE1α activation (Figs. 1 E and S1 F), indicating a specific stimulation mechanism independent of potential contamination with bacterial endotoxin (lipopolysaccharide [LPS]).

The micropinocytosis inhibitor amiloride (Koivusalo et al., 2010) blocked antigen-induced IRE1α stimulation in BMDCs (Figs. 1 E and S1 E), demonstrating a requirement for antigen internalization. Furthermore, CRISPR/Cas9-based disruption of the *TAP1* gene prevented IRE1α stimulation in response to ovalbumin (Fig. 1 F), indicating a requirement for peptide importation into the ER.

To corroborate the biological relevance of these observations, we used an alternative ex vivo maturation protocol to drive DC differentiation, which relies on Flt3-ligand (Flt3L; Brasel et al., 2000). Consistent with the IL-4–derived BMDCs, Flt3L-matured BMDCs also showed IRE1α activation in response to antigen pulsing (Fig. S1 F). As expected, Flt3L-derived BMDCs lacking the TLR adapter MyD88 failed to activate IRE1α in response to LPS (Martinon et al., 2010; Fig. S1 F); however, these cells showed unimpeded IRE1α stimulation in response to ovalbumin, further confirming that IRE1α activation in this setting is LPS independent. In contrast to BMDCs, mouse embryonic fibroblasts (MEFs), which are not expected to perform efficient antigen uptake, did not display detectable IRE1α activation after exposure to ovalbumin (Fig. S1 G), supporting a BMDC-specific mechanism of IRE1α engagement in response to extracellular protein or antigen. Taken together, these results demonstrate that a surge in DC exposure to an extracellular protein antigen leads to significant IRE1α activation above steady-state levels. This response is transient, occurs independently of LPS, and requires antigen uptake as well as importation of antigen-derived peptides into the ER. The uniqueness of DCs versus MEFs strengthens the physiological relevance of this activation.

### Antigen-derived peptides can directly engage IRE1α

**Figure 2.**
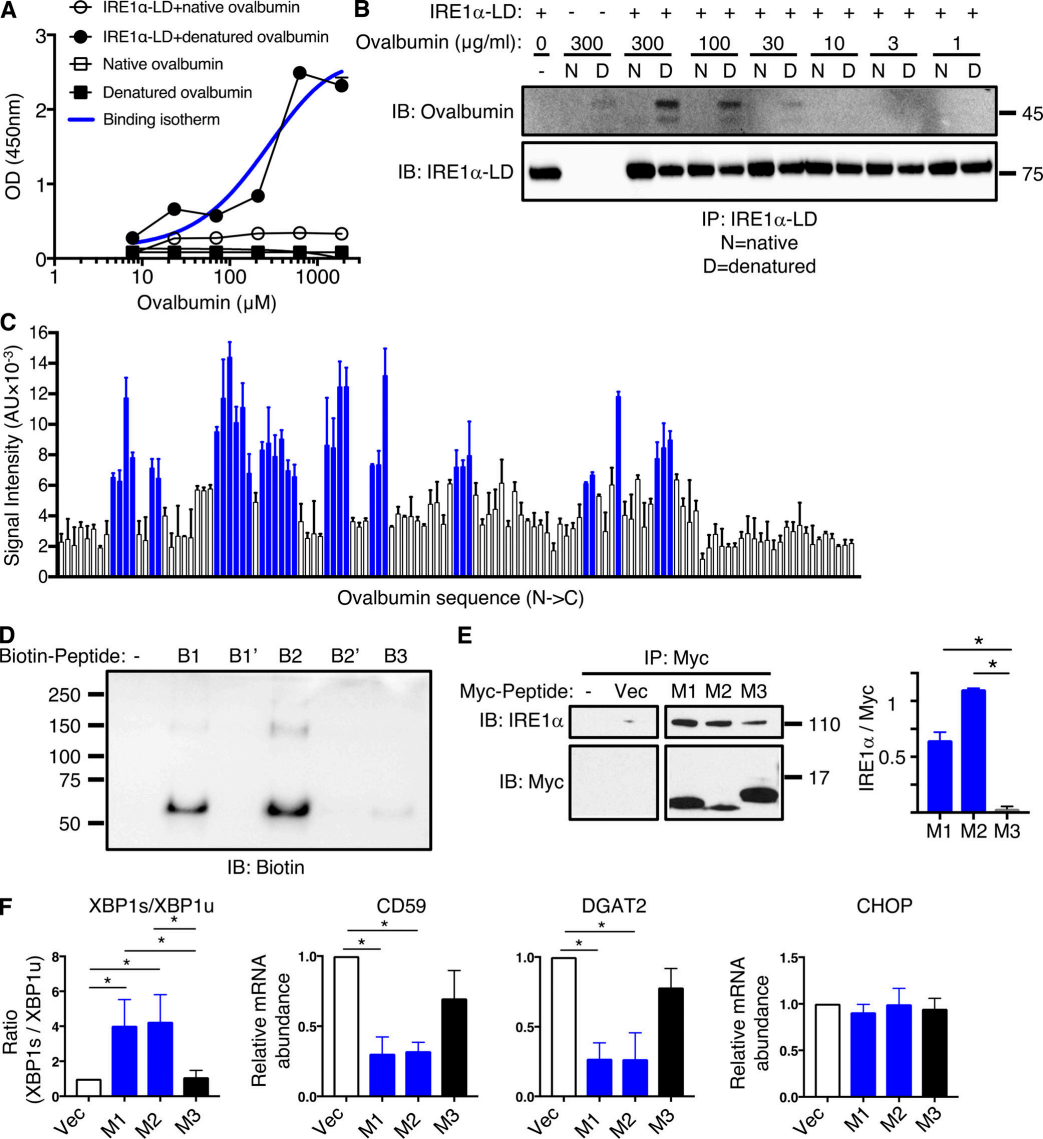
**Antigen-derived peptides can directly engage IRE1α. (A)** Polystyrene wells were coated with native or heat-denatured ovalbumin (10 μg/ml) and incubated with purified recombinant human IRE1α LD-Fc fusion protein (10 μg/ml), followed by colorimetric detection with an HRP-conjugated anti-human Fc antibody. **(B)** Native or heat-denatured ovalbumin at indicated concentrations was incubated with IRE1α LD-Fc (10 μg/ml), immunoprecipitated via monoclonal anti-IRE1α LD antibody, and analyzed by IB. **(C)** A tiled 18 aa-long peptide array spanning ovalbumin was incubated with IRE1α LD-Fc (500 nM), and an HRP-conjugated anti-human Fc antibody was used for detection. Blue bars represent bound peptides. **(D)** Biotin-tagged ovalbumin-based peptides (100 μM) were incubated with FLAG-tagged IRE1α LD (50 μM), cross-linked with disuccinimidyl suberate and analyzed by IB. B1, B2, B3 are WT peptides; B1′, B2′ are mutant peptides in which all hydrophobic residues were replaced by aspartate residues. **(E and F)** HEK293 cells were transfected with cDNA constructs encoding Myc-tagged peptides (M1, M2, M3) derived from corresponding ovalbumin regions (B1, B2, B3) and containing an ER-directed signal sequence; after 48 h analysis was performed by immunoprecipitation with anti-Myc antibody and IB for IRE1α or Myc (bar graph indicates signal ratio for IRE1α over Myc; E) or RT-qPCR analysis for mRNA levels of indicated transcripts (F). Graphs in A, C, and F represent mean ± SD from three independent technical repeats; images in B, D, and E represent at least two similar experiments, and molecular weights represent kD. Analysis was performed using unpaired, two-tailed *t* test; *, P ≤ 0.05. Source data are available for this figure: SourceData F2.

In the context of classical ER stress, otherwise buried hydrophobic segments of unfolded proteins can directly engage the ER-lumenal domain of IRE1α (Gardner and Walter, 2011). Accordingly, we reasoned that antigen-derived peptides may directly interact with IRE1α by mimicking the action of unfolded proteins. To examine this possibility, we first compared the ability of heat-denatured and native forms of the ovalbumin antigen to bind to a recombinant protein comprising the IRE1α lumenal domain fused to an Fc tag (IRE1α LD-Fc). Heat-denatured ovalbumin displayed specific and saturable binding to immobilized IRE1α LD-Fc, whereas native ovalbumin showed little binding over background (Fig. 2 A). We estimated an equilibrium dissociation constant (Kd) of ∼300 ± 75 μM for denatured ovalbumin, in line with affinities reported for unfolded-protein binding to IRE1α LD (Gardner and Walter, 2011). Co-immunoprecipitation studies confirmed concentration-dependent association of heat-denatured ovalbumin with IRE1α LD-Fc, whereas native ovalbumin again did not show appreciable binding (Fig. 2 B). Thus, denaturation-mediated unfolding of ovalbumin permits its direct binding to IRE1α’s LD.

**Figure S2.**
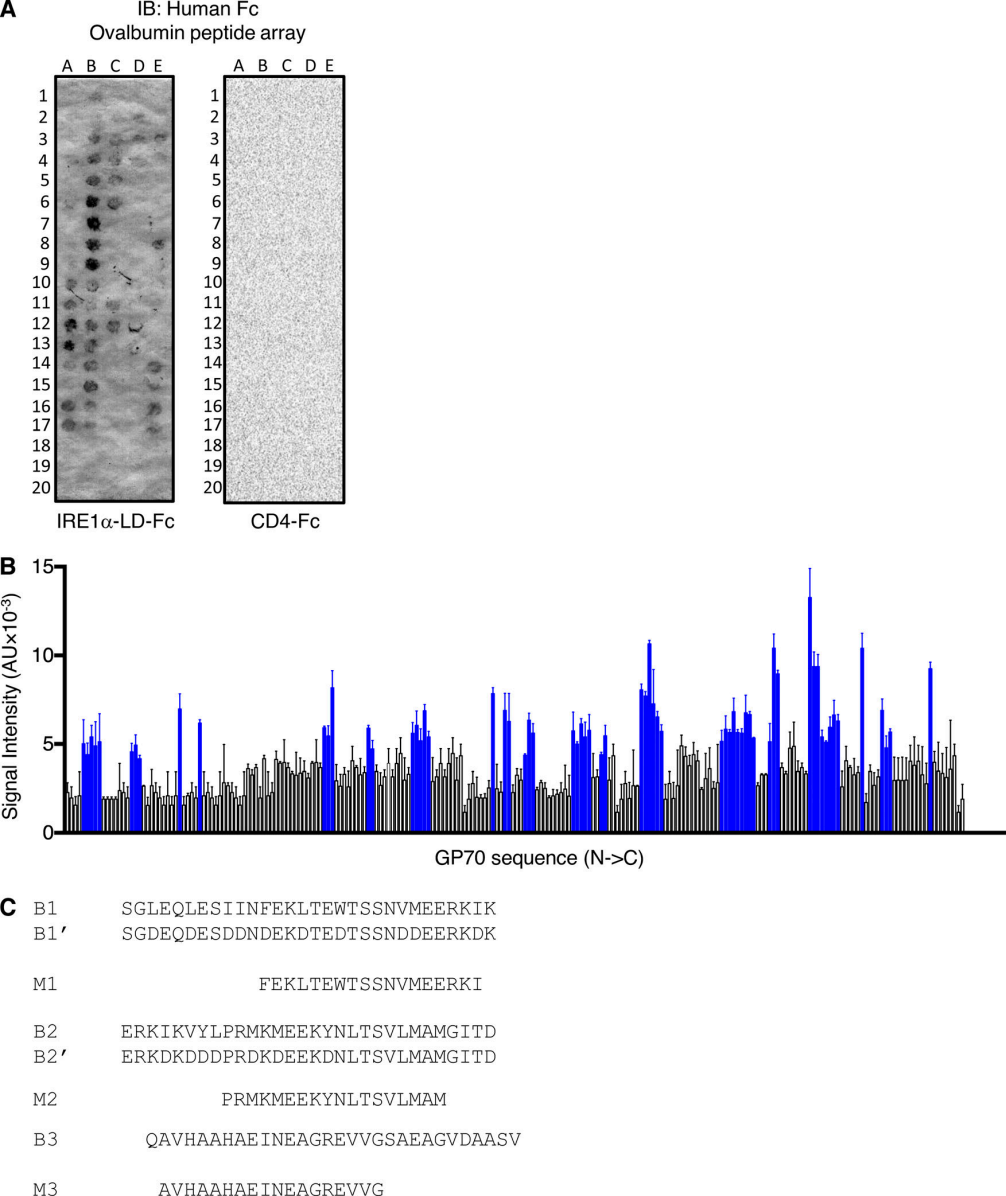
**Antigen-derived peptides can directly engage IRE1α. (A)** A tiled 18 aa-long peptide array spanning ovalbumin was incubated with IRE1α LD-Fc (500 nM) followed by IB analysis with an HRP-conjugated anti-human Fc antibody. **(B)** A tiled 18 aa-long peptide array spanning GP70 was incubated with IRE1α LD-Fc (500 nM) followed by colorimetric detection with an HRP-conjugated anti-human Fc antibody. Blue bars represent bound peptides. **(C)** Sequences of biotin-tagged peptides (labeled B) used in Fig. 1 D and Myc-tagged signal peptides (labeled M) used in Fig. 2, E and F. Bar graphs in B represent mean ± SD from three independent technical repeats. Source data are available for this figure: SourceData FS2.

To determine whether specific ovalbumin subsegments could interact with IRE1α, we generated a “tiled” peptide array spanning the polypeptide sequence, consisting of 18 amino acid–long synthetic peptides with a 3-residue overlap, as previously described (Gardner and Walter, 2011). We spotted the peptides on a membrane and examined binding of IRE1α LD-Fc, using horseradish peroxidase–based colorimetric detection. To test an independent antigen, we generated a similar peptide array based on the GP70 protein, which is expressed by CT26 colorectal cancer cells (Takeda et al., 2000). The analyses revealed that 34/123 ovalbumin peptides (clustered in 10 regions) and 64/210 GP70 peptides (19 regions) displayed significant binding to IRE1α LD-Fc (Fig. 2 C; and Fig. S2, A and B). In contrast, arrayed peptides derived from ovalbumin did not exhibit detectable binding to CD4-Fc under identical conditions (Fig. S2 A).

We next evaluated the importance of hydrophobic side-chains for peptide binding. We synthesized biotin-tagged peptides corresponding to two binding and one non-binding segment of the tiled ovalbumin array (B1, B2, and B3, respectively), and mutated variants of the binders with hydrophobic residues substituted by aspartic acids (B1′, B2′; Fig. S2 C). We incubated each peptide with FLAG-tagged IRE1α LD, stabilized bound complexes by chemical crosslinking, and visualized the products by anti-biotin immunoblotting. While peptide B3 showed no significant interaction, B1 and B2 exhibited specific binding, associating not only with monomers but also with apparent dimers or oligomers of IRE1α LD (Fig. 2 D). In contrast to B1 and B2, mutated peptides B1′ and B2′ failed to show significant binding, indicating a critical role of hydrophobic B1 and B2 side-chains for interaction with IRE1α LD.

To test specifically whether ovalbumin-based peptides can directly interact with IRE1α in a cellular setting, we employed a strategy that bypasses the unique DC features of antigen uptake, release from endosomes, proteasomal processing, and ER importation. To this end, we transfected HEK293 cells with cDNA constructs encoding shorter Myc-tagged versions of B1, B2, and B3 (M1, M2, and M3) fused to a signal sequence for direct ER targeting (Fig. S2 C). Immunoprecipitation with anti-Myc antibody followed by immunoblotting with anti-IRE1α revealed specific co-immunoprecipitation of Myc-tagged peptides with IRE1α (Fig. 2 E). Analyzing the signal ratios for IRE1α over Myc confirmed significantly greater IRE1α interaction with M1 and M2 as compared with M3. To further assess functional IRE1α engagement, we transfected U20S cells with the cDNA constructs encoding the M1, M2, or M3 peptides. We determined IRE1α activation by real-time quantitative PCR (RT-qPCR) analysis of mRNA transcripts for XBP1s and XBP1u as well as the RIDD targets CD59 and DGAT2; for comparison, we examined PERK activation by measuring CHOP mRNA levels. The M1 and M2 peptides functionally engaged IRE1α, as evident by upregulation of XBP1 splicing and depletion of RIDD targets CD59 and DGAT2, without significant changes detected in CHOP mRNA (Fig. 2 F); in contrast, peptide M3 showed no activation of either UPR branch above the vector controls. Thus, congruent with the results obtained with corresponding synthetic peptides in a cell-free setting (Fig. 2, D and E), ovalbumin-derived, ER-targeted peptides specifically bind to the ER-resident IRE1α protein within cells and selectively stimulate its RNase activity in a manner that corresponds to their ability to bind directly to IRE1α LD.

### IRE1α inhibition in BMDCs augments cross-presentation

**Figure 3.**
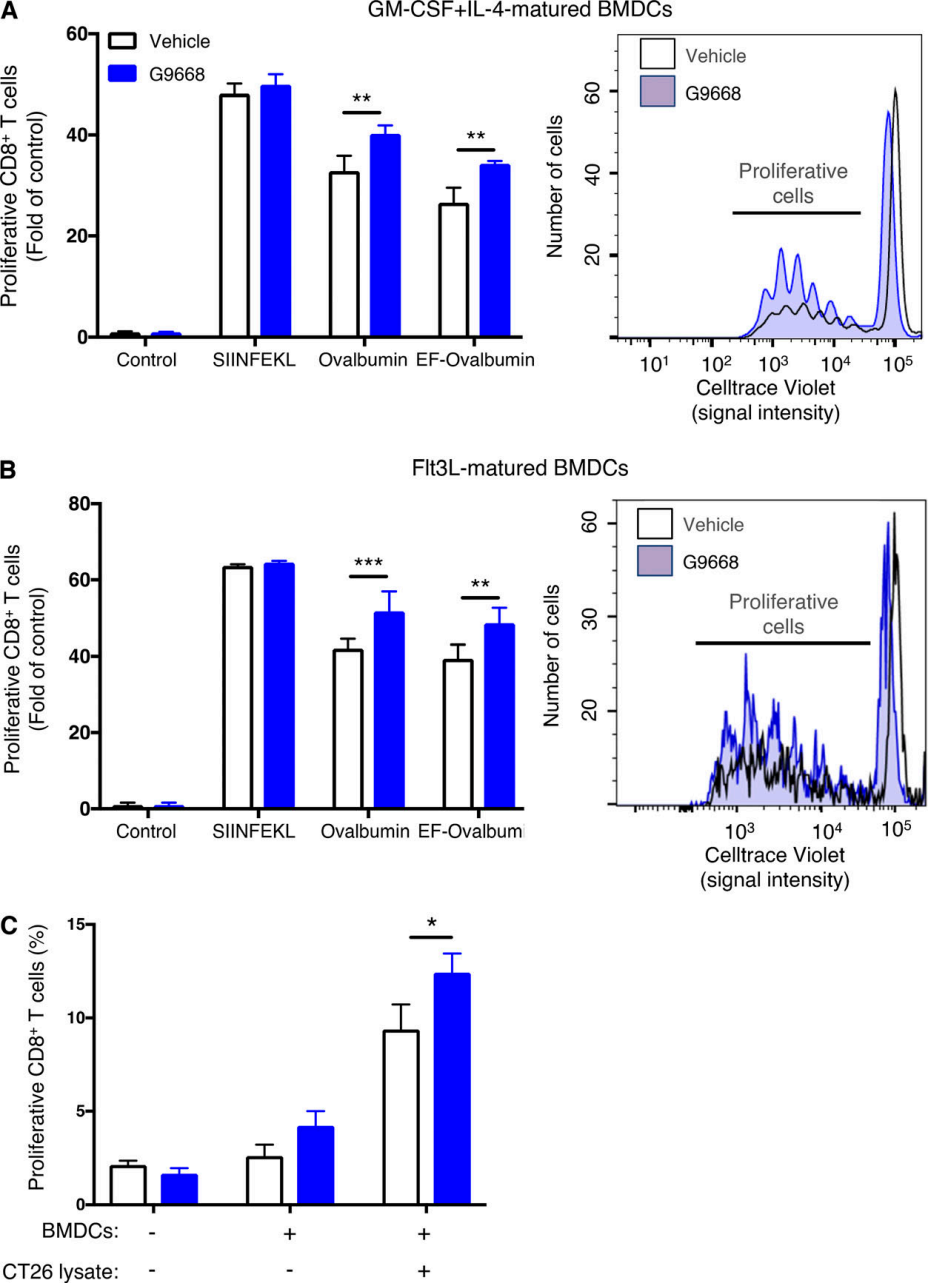
**IRE1α inhibition in BMDCs augments antigen cross-presentation to CD8**^**+**^ **T cells. (A and B)** GM-CSF + IL-4-matured (A) or Flt3L-matured (B) BMDCs were treated with LPS (10 μg/ml) for 2 h, pulsed with SIINFEKL (100 nM), ovalbumin (250 μg/ml) or EF-OVA (250 μg/ml) with or without G9668 (3 μM) for 24 h and subsequently co-cultured with magnetically separated splenic CD8^+^ OT-I T cells for 72 h, followed by assaying of T cell proliferation by flow cytometry analysis of Celltrace Violet staining loss. **(C)** GM-CSF + IL-4-matured BMDCs were treated with LPS (10 μg/ml) for 2 h, pulsed with lysates derived from CT26 cells (250 μg/ml protein) for 24 h and co-cultured with magnetically separated splenic CD8^+^ T cells from CT26 tumor-bearing mice for 72 h, followed by assaying of T cell proliferation by flow cytometry analysis of Celltrace Violet staining loss. Analysis was performed using unpaired, two-tailed *t* test; *, P ≤ 0.05; **, P ≤ 0.01; ***, P ≤ 0.001. Bar graphs in all panels represent mean ± SD from three independent biological repeats (*n* = 3 per repeat); A and B represent data from at least three independent experiments collated as fold of control for each experiment (*n* = 3 per experiment).

**Figure S3.**
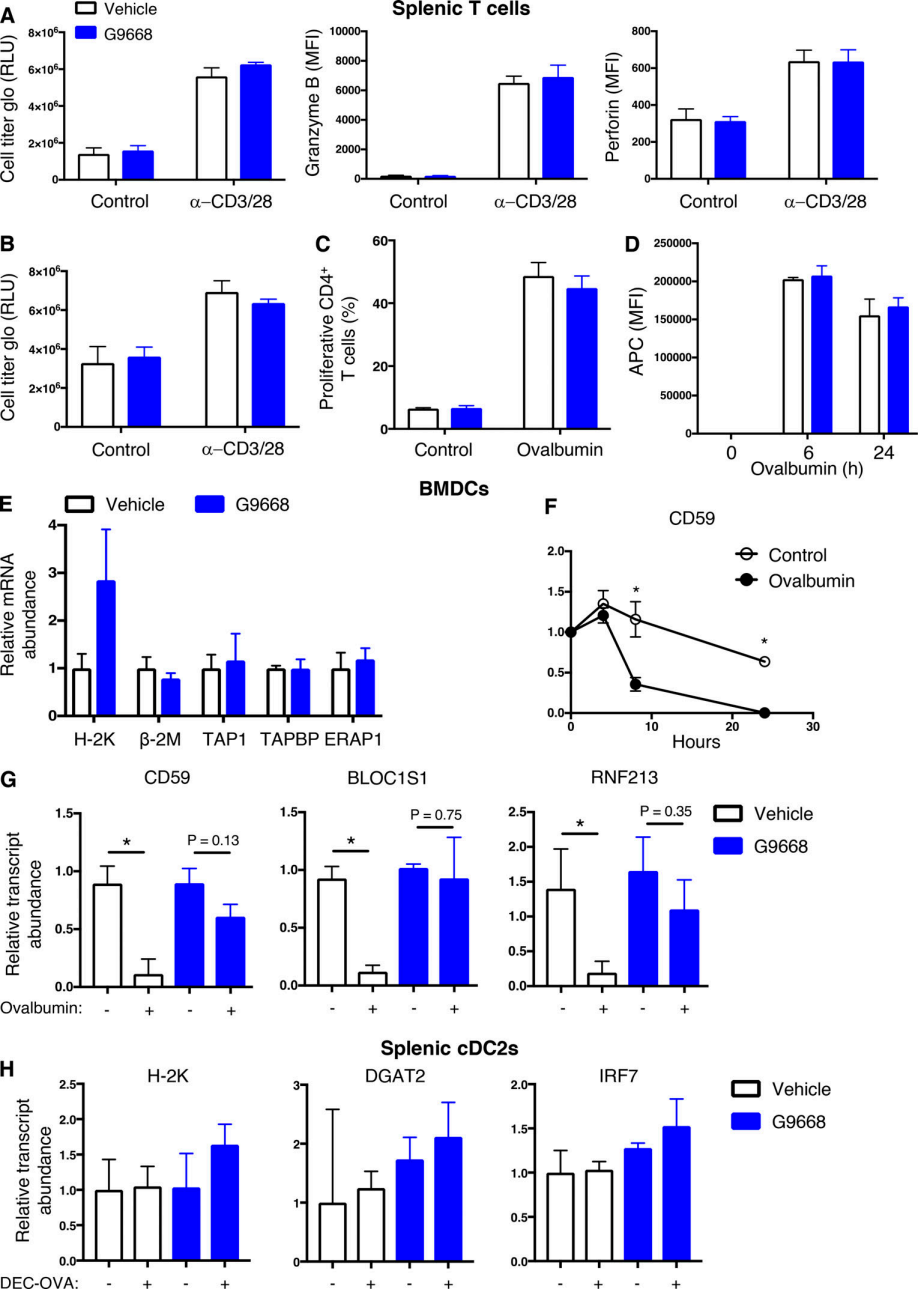
**IRE1α inhibition does not affect direct TCR-mediated activation of CD4**^**+**^ **and CD8**^**+**^ **T cells, MHC-II–restricted antigen presentation, and soluble antigen uptake by BMDCs; IRE1α RIDD activity specifically targets MHC-I heavy-chain transcripts. (A and B)** Magnetically separated splenic CD8^+^ OT-I (A) or CD4^+^ OT-II (B) T cells were activated by plate-bound anti-CD3 (2 μg/ml) and soluble anti-CD28 (8 μg/ml) antibodies in the absence or presence of G9668 (3 μM) for 72 h. Proliferation and activation were analyzed respectively by Cell Titer Glo and flow cytometry assay using Celltrace Violet signal loss. **(C)** GM-CSF + IL-4-matured BMDCs were treated with LPS (10 μg/ml) for 2 h, pulsed with ovalbumin (500 μg/ml) for 24 h with or without G9668 (3 μM), and subsequently co-cultured with magnetically separated CD4^+^ OT-II T cells for 72 h, followed by the assay of T cell proliferation by flow cytometry analysis of Celltrace Violet signal loss. **(D)** BMDCs were pulsed with APC-labelled ovalbumin (500 μg/ml) for indicated time points and analyzed for internalization of ovalbumin by flow cytometry. **(E)** BMDCs were pulsed with ovalbumin (500 μg/ml) for 8 h in the absence or presence of G9668 (3 μM), followed by RT-qPCR analysis of the indicated RIDD substrates. **(F and G)** BMDCs were treated with actinomycin D (2 μg/ml) to block de novo transcription and pulsed with ovalbumin (500 μg/ml) with or without G9668 (3 μM) for indicated time points (F) or for 8 h (G), followed by RT-qPCR analysis of indicated transcripts. **(H)** C57BL/6 mice were injected with Flt3L-Fc (i.v., 10 mg/kg) and 8 d later were treated with G9668 (p.o., 250 mg/kg, BID) for 24 h in combination with a single injection of DEC-OVA (i.v., 2.5 mg/kg) given 2.5 h before sacrifice. Viable splenic DCs (CD11c^+^ MHC-II^high^ F4/80^−^) were then gated and sorted for the cDC2 (CD103^−^ XCR1^−^ CD11b^+^) subpopulation. Sorted cells were analyzed by RT-qPCR for levels of indicated transcripts. Analysis was performed using unpaired, two-tailed *t* test; *, P ≤ 0.05. Graphs in A–G represent mean ± SD from three independent biological repeats (*n* = 3 per repeat), while bar graphs in H represent mean ± SD from two independent technical repeats. RLU, relative luminescence units; MFI, mean fluorescent intensity.

To examine whether antigen-induced IRE1α activation impacts cross-presentation, we pulsed LPS-primed BMDCs with ovalbumin in the absence or presence of G9668. We then tested BMDC capacity to activate mouse splenic OT-I CD8^+^ T cells, which express a transgenic TCR specific to the SIINFEKL epitope. During SIINFEKL pulsing, IRE1α inhibition with G9668 had little effect; however, upon ovalbumin pulsing, it significantly and reproducibly augmented subsequent induction of OT-I CD8^+^ T cell proliferation by ∼20% (Fig. 3 A). To ensure LPS-independent augmentation via IRE1α, we pulsed BMDCs with endotoxin-free ovalbumin (EF-OVA); comparably, IRE1α inhibition in this setting augmented OT-I CD8^+^ T cell proliferation by ∼29% (Fig. 3 A). Furthermore, G9668 treatment during pulsing of Flt3L-matured BMDCs with either ovalbumin or EF-OVA increased OT-I CD8^+^ T cell proliferation by 23 or 24%, respectively (Fig. 3 B). Importantly, G9668 did not directly affect proliferation or activation of naive CD4^+^ or CD8^+^ splenic T cells upon TCR stimulation with anti-CD3 plus anti-CD28 antibodies (Fig. S3, A and B). Moreover, in contrast to CD8^+^ T cell stimulation, IRE1α inhibition during ovalbumin pulsing did not alter MHC-II–restricted activation of splenic OT-II transgenic CD4^+^ T cells, which also harbor an ovalbumin-specific TCR transgene (Fig. S3 C). Taken together, these results indicate that protein-antigen pulsing of BMDCs leads to IRE1α activation, which in turn specifically dampens MHC-I–restricted antigen cross-presentation to CD8^+^ T cells. Functional inhibition of IRE1α reverses this curbing mechanism, enhancing antigen cross-presentation.

To investigate the impact of IRE1α inhibition on cross-presentation of tumor antigens, we subcutaneously inoculated BALB/c mice with CT26 cells and allowed tumors to form. We then isolated splenic CD8^+^ T cells (likely possessing TCRs that can recognize CT26 antigens) from these mice and co-incubated them with BMDCs pre-pulsed with CT26 cell lysates. Addition of G9668 augmented cross-presentation of CT26 epitopes to cognate splenic CD8^+^ T cells by ∼25% (Fig. 3 C), in keeping with ovalbumin cross-presentation. Thus, IRE1α inhibition in BMDCs enhances cross-presentation of tumor-derived antigens.

Next, we turned to interrogating which specific aspect of the cross-presentation process is modulated by IRE1α. IRE1α inhibition did not alter the uptake of fluorescently labeled ovalbumin by BMDCs (Fig. S3 D).

### IRE1α activation in DCs depletes MHC-I heavy-chain mRNAs via RIDD

Earlier work shows that, in lymph node–resident CD8^+^ DCs, RIDD constitutively suppresses mRNA transcripts encoding certain components of the cross-presentation machinery, such as TAPBP (Osorio et al., 2014). In ovalbumin-pulsed BMDCs, IRE1α inhibition minimally impacted mRNA levels of TAPBP, nor did it affect transcript abundance of MHC-I light-chain β-2 micro-globulin (β-2M), TAP1, or the ER aminopeptidase ERAP1; however, it markedly upregulated MHC-I H-2K heavy-chain mRNA levels by 2.8-fold (Fig. S3 E).

**Figure 4.**
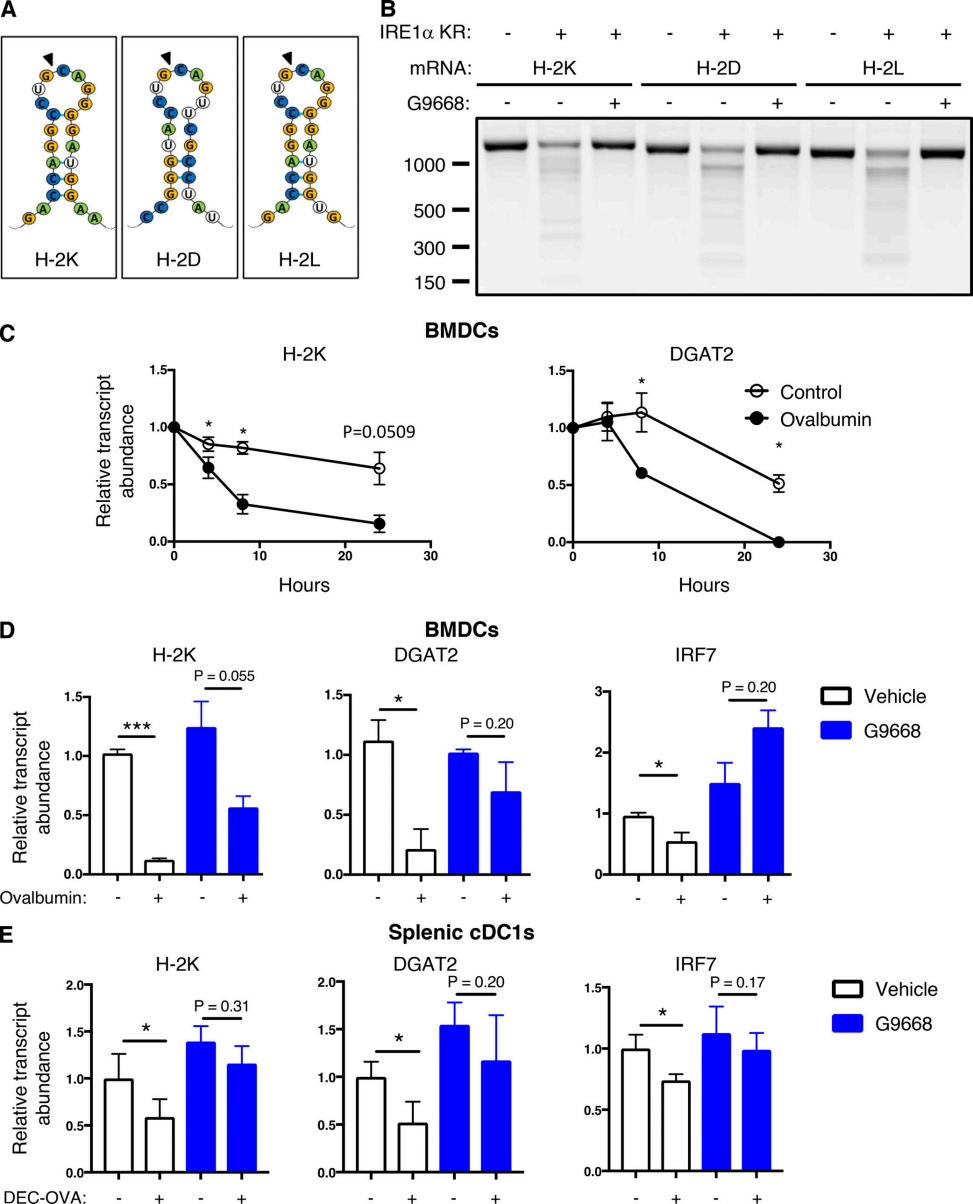
**IRE1α** **activation depletes MHC-I heavy-chain mRNAs via RIDD. (A)** Consensus stem-loop endomotifs for RIDD recognition in murine MHC-I heavy-chain H-2K, H-2D, and H-2L mRNA sequences. **(B)** Purified recombinant IRE1α kinase-RNase (KR) protein was incubated with RNA transcripts of H-2K, H-2D, and H-2L, and G9668 (10 μM), followed by agarose gel electrophoresis to determine transcript integrity. **(C and D)** BMDCs were treated with actinomycin D (2 μg/ml) to block de novo transcription and pulsed with ovalbumin (500 μg/ml) for indicated time points (C) or with G9668 (3 μM) for 8 h (D). Indicated transcripts were analyzed by RT-qPCR. **(E)** C57BL/6 mice were injected with Flt3L-Fc (i.v., 10 mg/kg) and 8 d later were treated with G9668 (p.o., 250 mg/kg, BID) for 24 h in combination with a single injection of DEC-OVA (i.v., 2.5 mg/kg) 2.5 h before sacrifice. Viable splenic DCs (CD11c^+^ MHC-II^high^ F4/80^−^) were gated and sorted cDC1 (CD103^+^ XCR1^+^ CD11b^−^) subpopulation cells were analyzed by RT-qPCR for indicated transcript levels. Statistical analysis was performed using unpaired, two-tailed *t* test; *, P ≤ 0.05; ***, P ≤ 0.001. Image in B represents three similar experiments, and molecular weights represent base pairs. Bar graphs in C and D represent mean ± SD from three independent biological repeats; bar graphs in E represent mean ± SD from two independent technical repeats. Source data are available for this figure: SourceData F4.

Therefore, we considered the possibility that IRE1α activation in response to antigen pulsing attenuates cross-presentation by decreasing MHC-I heavy-chain mRNAs through RIDD. Supporting this hypothesis, computational examination of mRNA sequences encoding the murine H-2K, H-2D, and H-2L heavy-chains using the gRIDD algorithm (Le Thomas et al., 2021) revealed the presence of consensus IRE1α-targeted stem-loop endomotifs (Fig. 4 A), whereas β-2M mRNA did not contain such sequences. To test whether IRE1α can directly cleave heavy-chain mRNAs, we incubated a phosphorylated recombinant protein comprising the cytoplasmic kinase-RNase module of IRE1α with RNA transcripts encoding H-2K, H-2D, and H-2L. IRE1α efficiently cleaved all three RNAs (Fig. 4 B), supporting the possibility that their RIDD-mediated degradation curbs cross-presentation.

To examine RIDD-mediated depletion in BMDCs, we prevented de novo transcription with actinomycin D. In control-pulsed BMDCs, the mRNA levels of H-2K, as well as the canonical RIDD targets DGAT2, and CD59 remained stable over a 24-h period; in contrast, in antigen-pulsed BMDCs, the levels of H-2K, DGAT2, and CD59 mRNAs markedly declined over time (Figs. 4 C and S3 F). Furthermore, the IRE1α inhibitor G9668 substantially rescued mRNAs encoding H-2K, DGAT2, and IRF7 (Fig. 4 D), as well as other RIDD targets, i.e., CD59, BLOC1S1, and RNF213 (Fig. S3 G).

To test whether these findings could be extended to antigen exposure of DCs in vivo, we enriched splenic DCs in C57BL/6 mice by pretreatment with Fc-fused Flt3 ligand (Flt3L-Fc) for 8 d (Tu et al., 2014). We then treated the mice orally with G9668 over a 24-h period, in conjunction with an i.v. injection—2.5 h before sacrifice—of an ovalbumin-fused anti-DEC205 antibody (DEC-OVA), which specifically and directly delivers ovalbumin to DCs (Boscardin et al., 2006; Hawiger et al., 2001). We then sorted viable splenic DCs (CD11c^+^ MHC-II^high^ F4/80^−^) and separated the cDC1 (CD103^+^ XCR1^+^ CD11b^−^) subpopulation, which performs efficient cross-presentation, and the cDC2 (CD103^−^ XCR1^−^ CD11b^+^) subpopulation, which is much less capable of this function (den Haan et al., 2000). In response to DEC-OVA injection, the cDC1 subpopulation showed a significant decrease in the mRNA transcript levels of H-2K and of the RIDD markers DGAT2 and IRF7, and this reduction was inhibited by G9668 treatment (Fig. 4 E). In contrast, the cDC2 subpopulation showed little change in the levels of these mRNAs in context of DEC-OVA exposure or treatment with G9668 (Fig. S3 H). These results indicate that IRE1α suppresses H-2K mRNA abundance via RIDD both upon ex vivo antigen pulsing of BMDCs and during in vivo antigen exposure of splenic cDC1s.

### IRE1α inhibition in tumor-bearing mice upregulates MHC-I on tumor DCs and augments CD8^+^ T cell engagement

**Figure S4.**
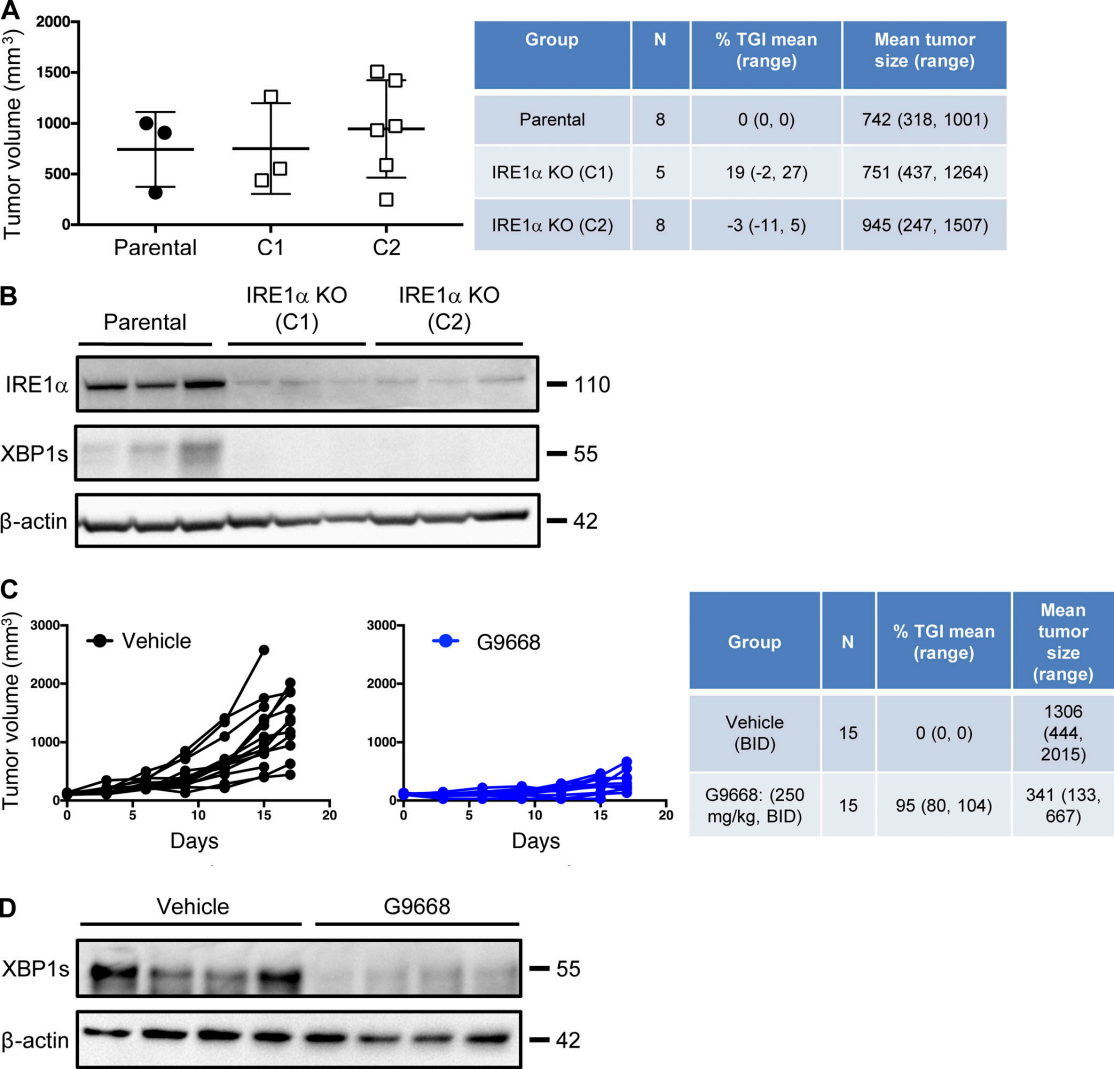
**IRE1α inhibition attenuates CT26 tumor growth. (A and B)** Animals were inoculated s.c. with parental or IRE1α KO CT26 cells, and tumor growth was measured over 27 d. Final day tumor measurements (A) and IB analysis (B) of IRE1α expression and activation are presented. **(C and D)** Mice were inoculated s.c. with parental CT26 cells, grouped out 7 d later, and treated with vehicle or G9668 (p.o., 250 mg/kg, BID). Growth trajectories of CT26 tumors in individual vehicle- and G9668-treated animals over 17 d (C) and IB analysis of total tumor lysates (D) are depicted. The number of animals included in each study is noted in corresponding tables. Scatter plots in A represents mean ± SD, and molecular weights in B and D represent kD. Source data are available for this figure: SourceData FS4.

**Figure 5.**
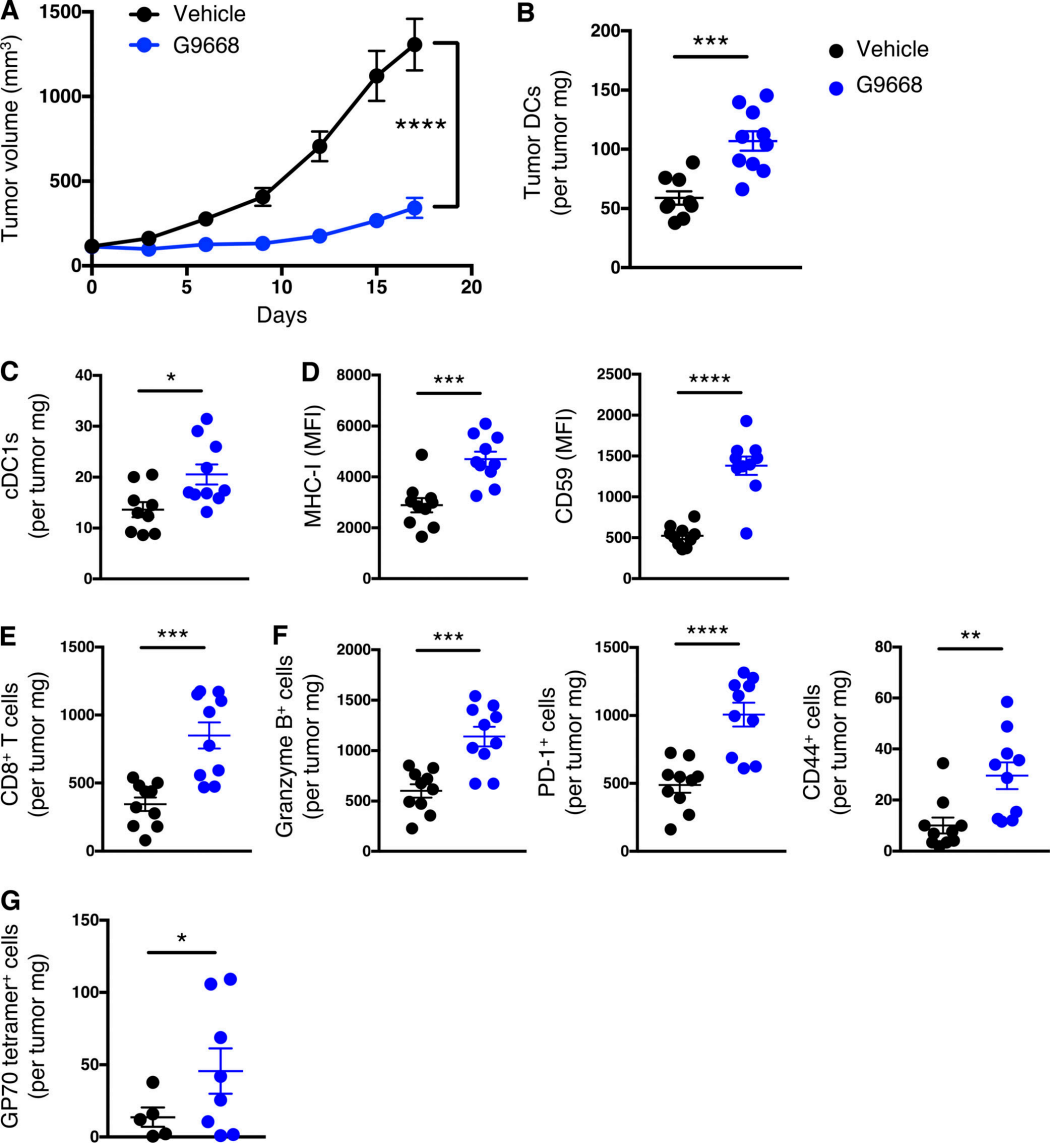
**IRE1α inhibition attenuates CT26 tumor growth in conjunction with enhanced MHC-I expression on tumor DCs and CD8**^**+**^ **T cell recruitment and activation.** Mice were inoculated s.c. with CT26 cells, grouped out 7 d later, and treated with vehicle or G9668 (p.o., 250 mg/kg, BID). **(A)** Growth trajectories of CT26 tumors in vehicle- and G9668-treated animals over 17 d (*n* = 15). **(B–G)** Flow cytometry analysis of tumor-infiltrating DCs and T cells from mice treated for 7 d. **(B–D)** Quantification of tumor-infiltrating total DCs (B) and cDC1s (C) and characterization of cDC1 expression of MHC-I and CD59 (D) by flow cytometry (*n* = 9 for vehicle-treated group and *n* = 10 for G9668-treated group). Total DCs were characterized as F4/80^low^, CD11c^+^, and MHC-II^high^, while cDC1s were characterized as CD103^+^ XCR1^+^ CD11b^−^. **(E–G)** Measurement of tumor-infiltrating CD8^+^ T cell abundance (E), expression of indicated activation markers (F; *n* = 9 for vehicle-treated group and *n* = 10 for G9668-treated group)**,** and binding of GP70 tetramers (G; *n* = 6 for vehicle and 8 for G9668 group). Analysis was performed using one-way ANOVA for A and unpaired, two-tailed *t*-test for B–G; *, P ≤ 0.05; **, P ≤ 0.01; ***, P ≤ 0.001; ****, P ≤ 0.0001. Scatter plots in all panels represent mean ± SEM.

To investigate IRE1α regulation of MHC-I in vivo, we turned to syngeneic tumor models in mice. Cell-autonomous knockout of *IRE1α* by CRISPR/Cas9 in CT26 colon cancer cells had little effect on subcutaneous CT26 tumor growth in vivo (Fig. S4, A and B). In contrast, systemic pharmacological inhibition of IRE1α with G9668 substantially attenuated tumor progression as compared with vehicle treatment (Fig. 5 A; and Fig. S4, C and D), suggesting enhancement of host-mediated anti-tumor activity. To elucidate potential immune effects, we analyzed the tumor-associated leukocyte populations by flow cytometry after 7 d of treatment. As compared with controls, tumors from G9668-treated mice showed significantly greater infiltration by CD11c^+^ MHC-II^high^ DCs (Fig. 5 B), specifically belonging to the cDC1 (XCR1^+^ CD103^+^) subpopulation (Fig. 5 C). Importantly, tumor-infiltrating cDC1s showed significantly higher surface levels of MHC-I, as well as the RIDD marker CD59 in G9668-treated mice as compared with controls (Fig. 5 D). Moreover, G9668 treatment led to significantly greater numbers of tumor-infiltrating cytotoxic CD8^+^ T cells (Fig. 5 E) and higher expression by these cells of the activation markers granzyme B, PD-1, and CD44 (Fig. 5 F). Staining with recombinant MHC-I tetramer complexes presenting the CT26 tumor antigen GP70 revealed significantly higher levels of tumor-infiltrating GP70-specific CD8^+^ T cells in G9668-treated mice (Fig. 5 G). Thus, although effects on NK cells (Dong et al., 2019), MDSC (Harnoss et al., 2020), or CD8^+^ T cells (Kamimura and Bevan, 2008) may also contribute, the attenuation of CT26 tumor growth by IRE1α inhibition is attributable, at least in part, to an elevated tumor infiltration, MHC-I expression, and tumor-antigen cross-presentation by cDC1s, which augments the recruitment and activation of tumor-reactive CD8^+^ T cells.

### Single-cell RNA sequencing (scRNAseq) indicates IRE1α regulation of MHC-I mRNA in tumor DCs

**Figure S5.**
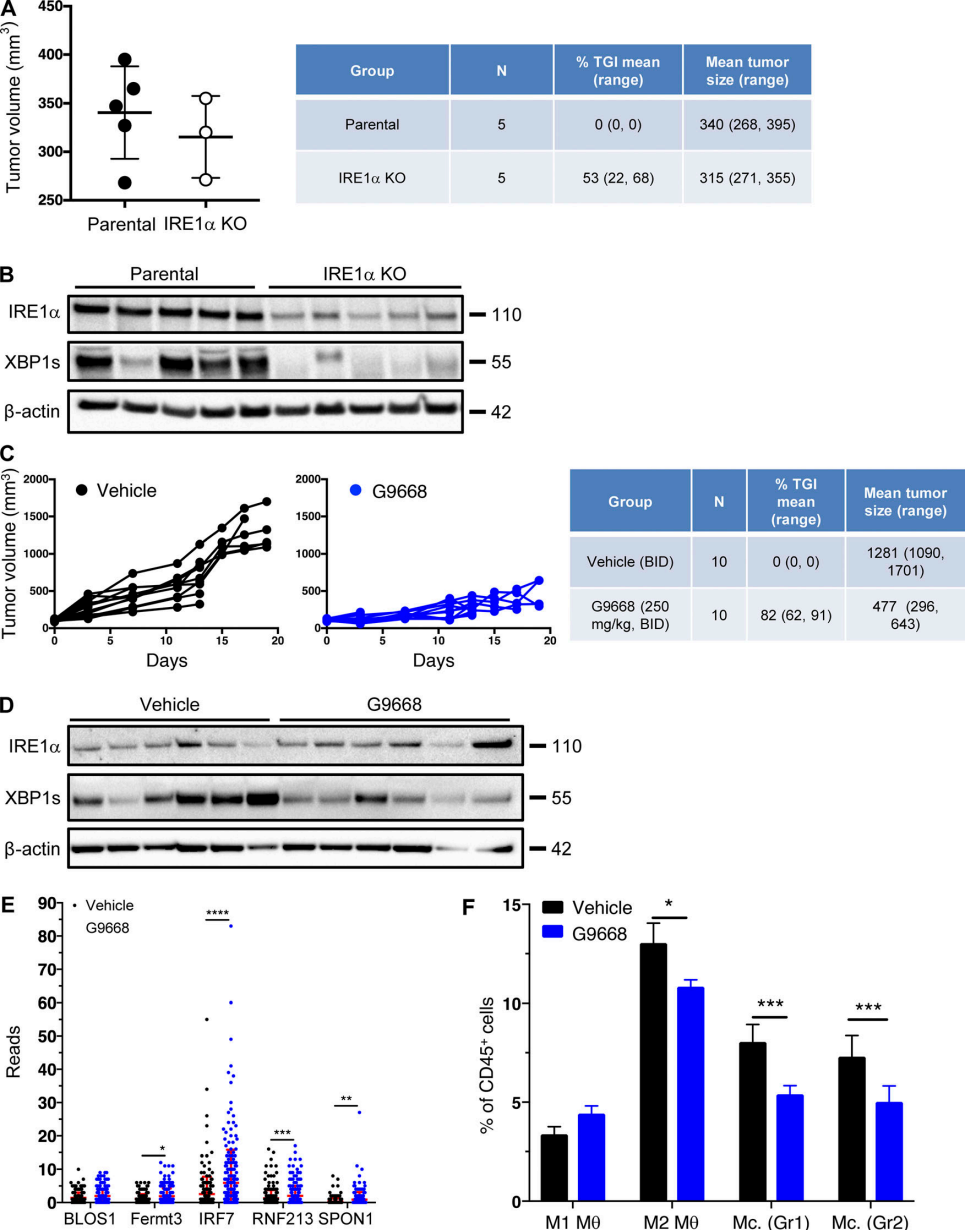
**IRE1α inhibition attenuates 4T1 tumor growth. (A and B)** Animals were inoculated s.c. with parental or IRE1α KO 4T1 cells and tumor growth was monitored over 25 d, with final measurements (A) and IB analysis of total tumor lysates (B) presented. **(C–F)** Mice were inoculated s.c. with parental 4T1 cells, grouped out 7 d later, and treated with G9668 (p.o., 250 mg/kg, BID). Tumor growth was measured over 19 d (C), and IRE1α expression and activation were analyzed by IB (D). **(E and F)** Mice were treated with G9668 for 6 d, and tumor-infiltrating leukocytes were then analyzed by scRNAseq. **(E)** Transcript levels of indicated genes characterized as RIDD targets in tumor-infiltrating DCs (256 reads per condition). **(F)** Relative abundance of group 1 (Hcar) and group 2 (Hilpda) tumor-infiltrating monocytes (Mc.) and M1- or M2-polarized macrophages (θ) in vehicle- and G9668-treated animals. *, P ≤ 0.05; **, P ≤ 0.01; ***, P ≤ 0.001; ****, P ≤ 0.0001. The number of animals included in each study is noted in corresponding tables. Scatter plots in A, E, and F represent mean ± SD, and molecular weights in B and D represent kD. Source data are available for this figure: SourceData FS5.

**Figure 6.**
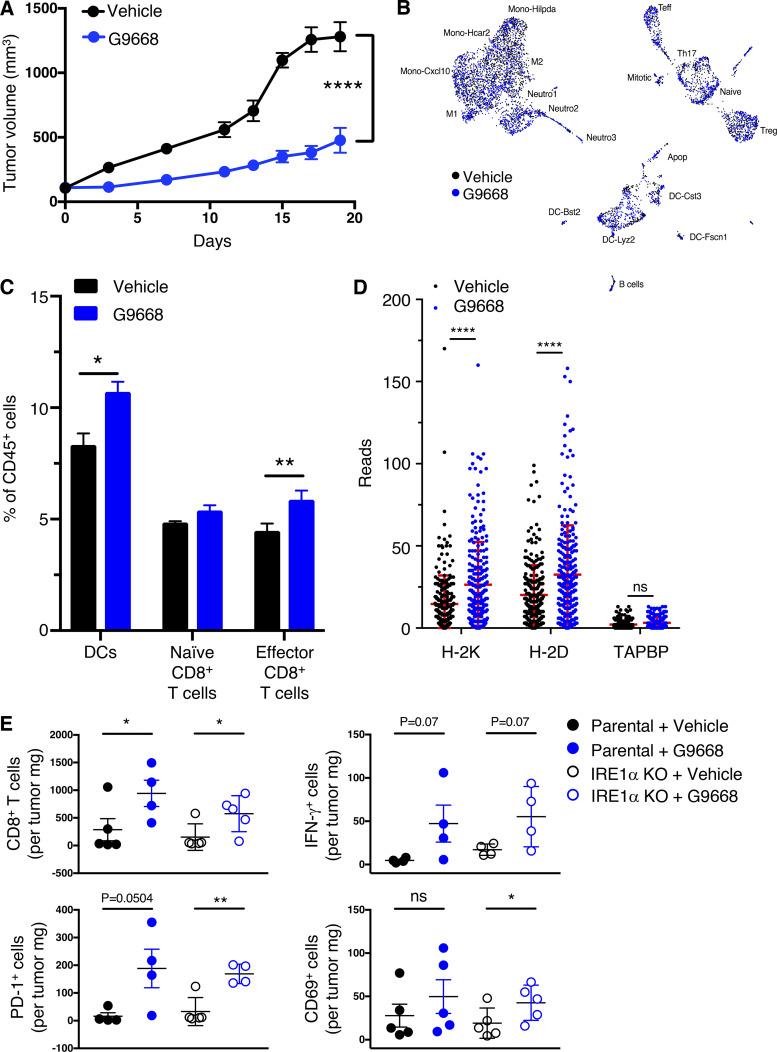
**IRE1α inhibition attenuates 4T1 tumor growth in conjunction with increased myeloid MHC-I mRNA levels and DC and CD8**^**+**^ **T cell tumor infiltration. (A–D)** Mice were inoculated s.c. with 4T1 cells, grouped out 7 d later, and treated with vehicle or G9668 (p.o., 250 mg/kg, BID). **(A)** Tumor growth trajectories were measured over 19 d (*n* = 15). **(B–D)** Mice were treated with G9668 for 6 d and then sacrificed. **(B)** Representation of tumor-infiltrating leukocytes by t-distributed stochastic neighbor embedding plots from scRNAseq data. **(C)** Abundance of tumor-infiltrating DCs and CD8^+^ T cells in vehicle- and G9668-treated animals. **(D)** Transcript levels of indicated genes in tumor-infiltrating DCs (256 reads per condition). **(E)** Mice were inoculated s.c. with parental or IRE1α KO 4T1 cells, grouped out 7 d later, and treated with vehicle or G9668 (p.o., 250 mg/kg, BID; *n* = 5). Tumor infiltration by CD8^+^ T cells and expression of indicated activation markers were assayed by flow cytometry. Analysis was performed using one-way ANOVA for A and unpaired, two-tailed *t* test for C–E; *, P ≤ 0.05; **, P ≤ 0.01; ****, P ≤ 0.0001. Scatter plots represent mean ± SEM in A and mean ± SD in C, D, and E.

To examine an additional tumor model, we used syngeneic 4T1 triple-negative breast cancer (TNBC) cells. In contrast to the CT26 model (Fig. S4, A and B), cell-autonomous knockout of *IRE1α* in 4T1 cells caused notable tumor-growth inhibition (TGI) of 53% (Fig. S5, A and B). Nevertheless, systemic treatment of mice bearing parental *IRE1α* wildtype 4T1 tumors with G9668 led to a stronger TGI of 82% (Fig. 6 A; and Fig. S5, C and D), suggesting both cell-autonomous and host-mediated anti-tumor effects. The myeloid compartment in tumors has been systematically studied by scRNAseq (Cheng et al., 2021; Mariathasan et al., 2018). We performed scRNAseq after 6 d of treatment to analyze the tumor leukocytic populations. Tumors in G9668-treated mice showed enrichment in both DCs and CD8^+^ T effector cells, but not in naive T cells (Fig. 6, B and C). Importantly, tumor-infiltrating DCs in G9668-treated mice displayed significantly higher mRNA levels of H-2K and H-2D heavy-chains, though not of TAPBP transcripts (Fig. 6 D). CD59 and DGAT2 mRNAs were insufficiently abundant to enable accurate quantification, but five other RIDD targets that were detected, i.e., BLOC1S1, FERMT3, IRF7, RNF213, and SPON1, showed significant increases (Fig. S5 E), confirming RIDD inhibition. Tumors in G9668-treated mice had unaltered levels of M1 macrophages, but showed significantly fewer M2 macrophages and monocytes as compared with controls (Fig. S5 F). Flow cytometric analysis after 6 d of treatment showed that IRE1α inhibition increased tumor infiltration by cytotoxic CD8^+^ T cells and their expression of the activation markers IFN-γ, PD-1, and CD69, independent of *IRE1α* status in the malignant cells (Fig. 6 E). These results demonstrate that IRE1α inhibition in tumor-associated DCs increases MHC-I expression, which likely contributes to enhanced engagement of tumor-infiltrating cytotoxic CD8^+^ T cells.

### Systemic IRE1α inhibition cooperates with immune-checkpoint blockade

**Figure S6.**
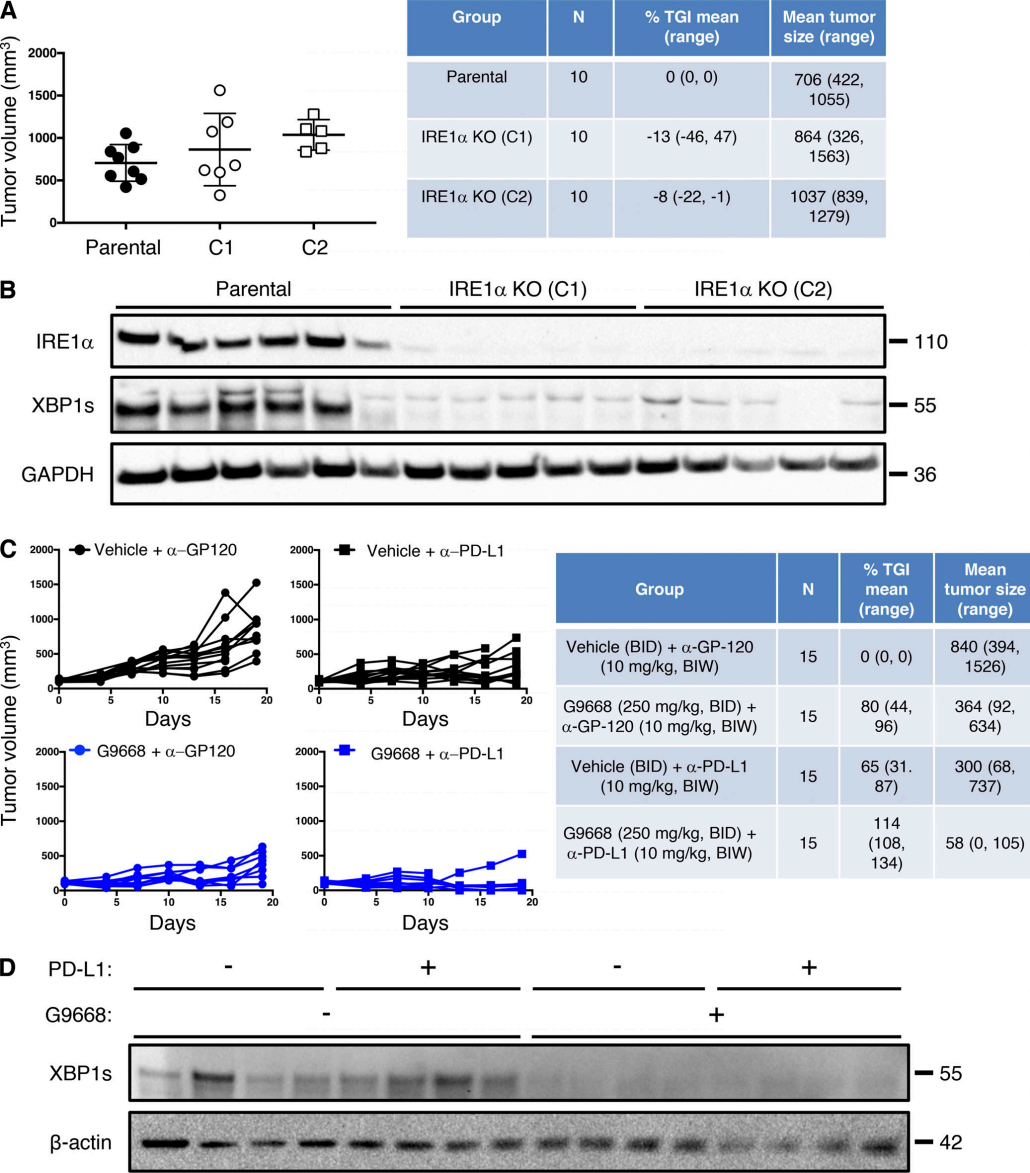
**IRE1α inhibition attenuates EMT6 tumor growth. (A and B)** Mice were inoculated orthotopically with parental or IRE1α KO EMT6 cells and tumor growth was measured over 24 d, with final tumor measurements (A) and IB analysis of total tumor lysates (B) presented. **(C and D)** Mice were inoculated orthotopically with parental EMT6 cells, grouped out 7 d later, and treated with vehicle, G9668 (p.o., 250 mg/kg, BID), anti–PD-L1 antibody (i.v., 10 mg/kg at first dose, i.p. 5 mg/kg BIW thereafter), or the combination. Tumor growth trajectories were measured over 19 d (C), and IRE1α activation was analyzed by IB (D). The number of animals included in each study is noted in corresponding tables. Scatter plots in A represent mean ± SD, and molecular weights in C and D represent kD. Source data are available for this figure: SourceData FS6.

**Figure 7.**
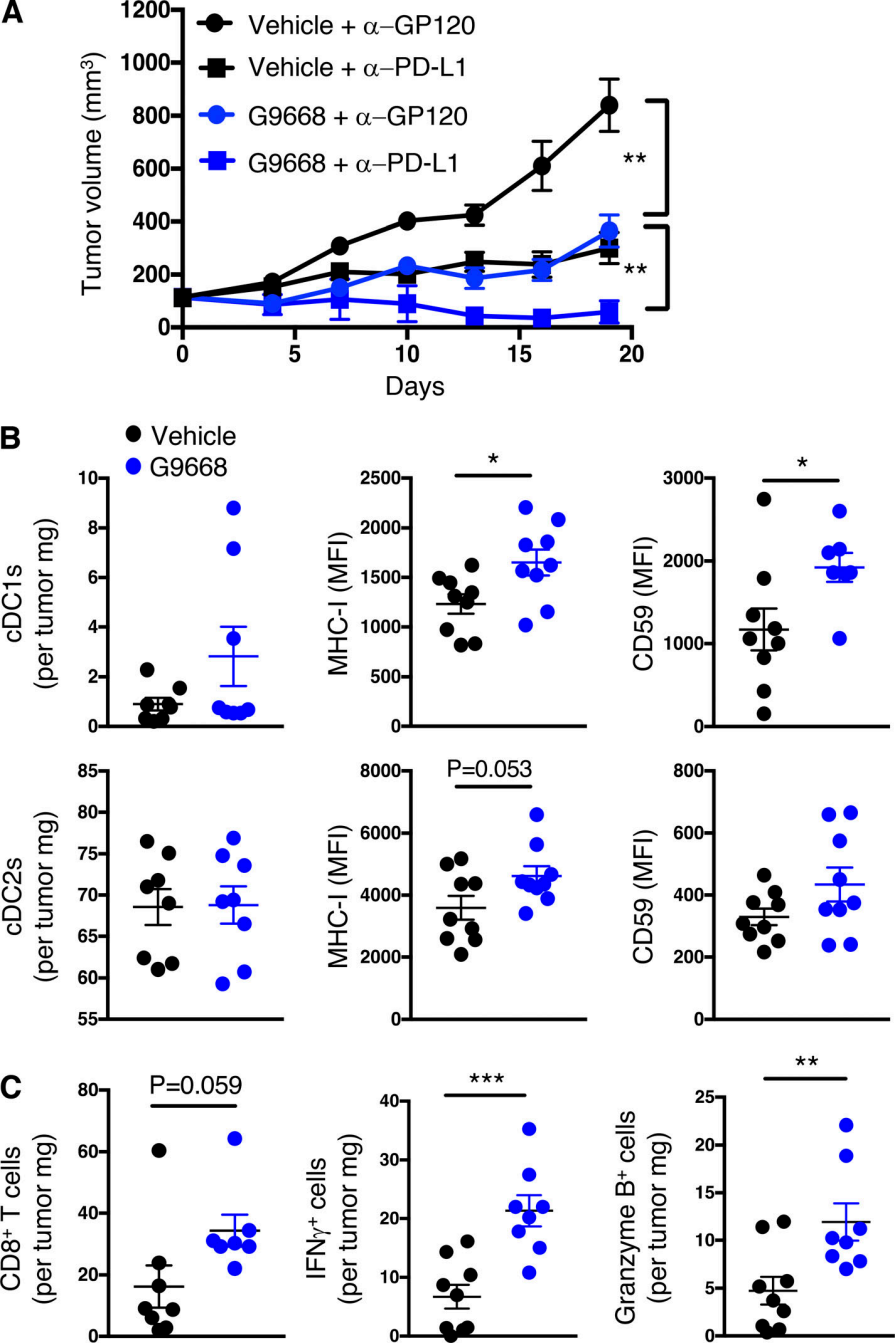
**IRE1α inhibition attenuates EMT6 tumor growth and synergizes with anti–PD-L1 treatment in conjunction with increased DC and CD8**^**+**^ **T cell tumor infiltration and activation.** Mice were inoculated orthotopically with EMT6 cells in the mammary fat pad, grouped out 7 d later, and treated with vehicle, G9668 (p.o., 250 mg/kg, BID), anti–PD-L1 antibody (i.v., 10 mg/kg at first dose, i.p., 5 mg/kg BIW thereafter), or the combination. **(A)** Tumor growth trajectories were measured over 19 d (*n* = 15). **(B and C)** Mice were treated with G9668 for 7 d and then sacrificed (*n* = 9). **(B)** Abundance of tumor cDC1s and cDC2s, as well as expression of MHC-I and CD59, were measured by flow cytometry. Total DCs were characterized as F4/80^low^, CD11c^+^ and MHC-II^high^, while cDC1s were further characterized as CD103^+^ XCR1^+^ CD11b^−^, and cDC2s were characterized as CD103^−^ XCR1^−^ CD11b^+^. MFI, mean fluorescence intensity. **(C)** Abundance and activation marker expression of tumor-infiltrating CD8^+^ T cells were assayed by flow cytometry. Analysis was performed using one-way ANOVA for A and unpaired, two-tailed *t* test for B and C; *, P ≤ 0.05; **, P ≤ 0.01; ***, P ≤ 0.001. Scatter plots in all panels represent mean ± SEM.

Although immune-checkpoint disruption has transformed patient benefit in a number of cancer settings (Marinelli et al., 2020), further advances are needed to achieve wider effectiveness. To investigate whether IRE1α inhibition would complement immune-checkpoint blockade, we turned to the orthotopic EMT6 TNBC model, previously found to exhibit partial responsiveness to anti–PD-L1 antibody therapy upon implantation in the mouse mammary fat pad (Mariathasan et al., 2018). Similar to the CT26 model, cell-autonomous *IRE1α* KO in EMT6 cells had minimal impact on tumor growth (Fig. S6, A and B), identifying an additional suitable model for interrogating the impact of IRE1α inhibition on immune modulation. Treatment of EMT6 tumor-bearing mice with either the anti-mouse PD-L1 monoclonal antibody 6E11 or the IRE1α inhibitor G9668 partially impaired EMT6 tumor progression (Fig. 7 A; and Fig. S6, C and D). Remarkably, combined administration of 6E11 and G9668 led to frank tumor regression with a mean TGI rate of 114%, resulting in significantly better efficacy than either monotherapy (P < 0.01). In keeping with the other models, treatment of EMT6 tumor-bearing mice with G9668 for 7 d significantly increased surface levels of MHC-I and CD59 in tumor-infiltrating cDC1s (Fig. 7 B). Moreover, G9668 increased tumor invasion by cytotoxic CD8^+^ T cells and their expression of the activation markers IFN-γ and granzyme B (Fig. 7 C). Thus, IRE1α inhibition effectively complements PD-L1–based immune-checkpoint disruption to reverse orthotopic EMT6 tumor progression.

## Discussion

The cell-biological mechanism of IRE1α activation in DCs in the absence of classical ER stress has been elusive (Iwakoshi et al., 2007; Medel et al., 2018; Osorio et al., 2014; Tavernier et al., 2017). Our present findings reveal that acute exposure of DCs to pulsed protein antigens drives intracellular IRE1α activation above steady-state levels through an LPS-independent mechanism that is analogous to direct engagement of IRE1α by unfolded proteins under canonical ER stress. We further show that antigen-induced IRE1α activity curbs cross-presentation by DCs through RIDD-mediated depletion of MHC-I heavy-chain mRNAs. This functional consequence likely represents a negative feedback loop that fine-tunes antigen cross-presentation, perhaps to prevent inappropriate or excessive T cell activation during sterile tissue injury. Disruption of this negative feedback by inhibiting IRE1α appears to cooperate effectively with immune-checkpoint blockade to enhance anti-tumor immune responses, suggesting unique potential for therapeutic translation.

Our BMDC studies showed that antigen pulsing selectively engages IRE1α with no measurable PERK activation and only minimal ATF6 stimulation, thus excluding general UPR activation as a key mechanistic driver. This contrasts with plasmacytoid DCs, which do not support cross-presentation, and interestingly display constitutive activation of PERK (Mendes et al., 2021). BMDC pulsing with distinct protein antigens or cancer-cell lysates induced significant levels of IRE1α activity. Although these levels were markedly weaker than those induced by strong pharmacological ER stressors, antigenic IRE1α stimulation was highly reproducible, reaching peak intensity within 2–4 h of exposure and then declining. The rapid yet transient activation kinetics are consistent with the time frame of antigen exposure, uptake, processing, and ER entry. Indeed, further mechanistic dissection indicated that antigen-driven IRE1α stimulation requires both pinocytosis and ER importation events, but not TLR signaling.

IRE1α resides in the ER membrane and responds through its LD to unfolded or misfolded proteins during canonical ER stress (Hetz, 2012; Walter and Ron, 2011). Based on the observation that antigen-induced IRE1α activation required TAP1—a critical mediator for importing antigen-derived peptides into the ER—we reasoned that antigen-based peptides entering the ER may directly engage IRE1α by masquerading as unfolded or misfolded proteins. We obtained several lines of evidence in support of this idea. First, although native ovalbumin failed to interact with the IRE1α LD, unfolded heat-denatured ovalbumin was capable of direct LD binding with affinity comparable with that of other unfolded proteins (Gardner and Walter, 2011). Second, specific ovalbumin-based peptides bound to the IRE1α LD in a manner that required hydrophobic amino acid side-chains. Third, cellular expression of ER-directed ovalbumin-based peptides demonstrated congruent interaction with, and functional engagement of, cellular IRE1α but not PERK. The lack of marked stimulation of other UPR branches in response to antigen pulsing further supports a direct IRE1α activation mode independent of BiP. Hence, although a nascent protein that is incorrectly folded by the ER and a peptide that lacks 3D structure due to proteolytic cleavage of its parent molecule are distinct, both can be sensed by IRE1α. While any protein-producing cell may harbor some constitutive level of peptide–IRE1α interactions, our data suggest that DCs are uniquely poised for significant IRE1α activation above steady-state upon acute exposure to an external protein antigen. The rise in extracellular protein concentration during our pulsing experiments (up to ∼60% on a molar basis) conceivably reflects surges in antigen exposure that DCs may encounter in the context of dying cells within the tumor microenvironment (TME). For cDC1s, which are highly specialized in antigen cross-presentation, such IRE1α activation can have distinct biological consequences. The recent discovery of pervasive functional peptide translation in cells (Chen et al., 2020) raises an intriguing question of whether additional peptide-based modalities besides antigen processing may similarly engage IRE1α. In future follow-up studies, it would be interesting to interrogate the functional importance of IRE1α in distinct DC subpopulations in vivo by employing genetic strategies to disrupt IRE1α or TAP in specific subsets of DCs.

Earlier work interrogating the involvement of IRE1α in DC regulation relied primarily on *XBP1* KO—a strategy that does not completely disrupt, and in some cases even augments, IRE1α kinase-RNase activity. In CD8^+^ DCs, *XBP1* KO caused RIDD hyper-activation, which led to mRNA depletion of certain components of the cross-presentation machinery, i.e., LAMP-1, TAPBP, and β2M (Osorio et al., 2014). In contrast, BMDC pulsing with melanoma cell lysates activated XBP1 splicing but not RIDD, and XBP1s appeared to promote, rather than disrupt, efficient melanoma antigen cross-presentation (Medel et al., 2018). Direct infection of BMDCs by *Toxoplasma gondii* induced IRE1α activation via MyD88-dependent TLR signaling, with decreased cross-presentation on *XBP1* KO with partial IRE1α disruption (Poncet et al., 2021). However, this partial disruption of IRE1α signaling left the biological consequence of IRE1α engagement during antigen cross-presentation incompletely understood.

To impede enzymatic activity more fully, we identified the highly selective and potent kinase-based IRE1α inhibitor G9668, which blocks both the kinase and RNase activities of IRE1α. Indeed, G9668 fully disrupted antigen-induced IRE1α activation in BMDCs, preventing IRE1α auto-phosphorylation, as well as consequent RNase-dependent XBP1 splicing and RIDD. While G9668 did not alter DC-surface presentation of ovalbumin-derived SIINFEKL peptide to OT-I CD8^+^ T cells, it augmented cross-presentation of pulsed full-length ovalbumin, confirming the requirement of intracellular events for IRE1α activation. Of note, G9668 did neither alter MHC-II–restricted antigen presentation to OT-II CD4^+^ T cells nor did it affect co-stimulation of naive CD8^+^ or CD4^+^ T cells. Enhancement by G9668 was not limited to the OT-I model, as it also applied to cross-presentation of CT26-derived antigens to splenic CD8^+^ T cells from CT26 tumor-bearing mice. Thus, the engagement of IRE1α during antigen processing within DCs selectively curtails MHC-I–restricted cross-presentation, providing negative feedback to modulate T cell activation.

Our investigation of how IRE1α curtails cross-presentation underscores RIDD as an important mechanism that downregulates mRNAs encoding MHC-I heavy chains. This agrees in principle with the earlier observation of RIDD-mediated depletion of other cross-presentation components (Osorio et al., 2014; Tavernier et al., 2017), although the specific RIDD targets differ. Distinct mRNAs may be affected by more subtle RIDD activation via antigen as compared with hyper-activation by *XBP1* KO. Our analysis revealed the presence of consensus stem-loop endomotifs within each of the three mouse MHC-I heavy-chain mRNAs, i.e., H-2K, H-2D, and H-2L. We verified the cleavage of all three transcripts by the phosphorylated kinase-RNase module of IRE1α in vitro, as well as of H-2K mRNA in antigen-pulsed BMDCs. Moreover, IRE1α inhibition prevented DEC-OVA–induced depletion of H-2K mRNA in the splenic cDC1 subpopulation, demonstrating that IRE1α suppresses MHC-I expression during antigen pulsing in vivo. Furthermore, as indicated by scRNAseq data, IRE1α inhibition elevated H-2K and H-2D transcript levels in tumor-associated DCs, providing validation of this mechanism in the TME. IRE1α can also perform more promiscuous RNase activity termed RIDDLE (Le Thomas et al., 2021), which might account for some divergence of RIDD-targeted mRNAs in different settings.

In three syngeneic tumor models, systemic treatment with the IRE1α inhibitor attenuated tumor growth more strongly than did *IRE1α* disruption selectively in the malignant cells, indicating that IRE1α activity in the TME supports tumor growth. Flow cytometry and scRNAseq analyses demonstrated that systemic IRE1α inhibition increased MHC-I heavy-chain transcript and surface-protein levels in tumor-infiltrating DCs, mirroring the in vitro BMDC experiments. These changes occurred in conjunction with enhanced tumor infiltration and activation of CD8^+^ T cells. Elevated MHC-I tetramer staining further strengthened the possibility of functional linkage between the enhancement of highly specialized cDC1-mediated cross-presentation and CD8^+^ T cell engagement. Finally, combined treatment with anti–PD-L1 antibody and G9668 in the EMT6 TNBC model, which was only partially responsive to anti–PD-L1, led to clear tumor regression, suggesting a non-redundant complementarity of these two modalities. While improved cross-presentation likely contributes to the therapeutic benefit of IRE1α inhibition against tumors, additional effects on the TME may participate as well.

In conclusion, our cell-biological studies in DCs indicate that antigen-derived peptides can directly engage IRE1α, helping to explain DC activation of this ER-stress sensor in the absence of classical ER stress. Furthermore, by fully blocking IRE1α’s enzymatic function, we have discovered that IRE1α controls a negative feedback loop, depleting MHC-I heavy-chain mRNAs via RIDD, to dampen cross-presentation and curtail consequent CD8^+^ T cell activation. Excitingly, small-molecule IRE1α inhibition disrupts this negative feedback, which may be leveraged to augment cancer immunotherapy—particularly in combination with anti–PD-L1. Our findings conceptually advance earlier work implicating XBP1s in functional regulation of immune cells in the TME (Cubillos-Ruiz et al., 2015) and previous studies on tumoral IRE1α disruption (Harnoss et al., 2020; Harnoss et al., 2019; Healy et al., 2009; Logue et al., 2018).

## Materials & methods

### Cell cultures, BMDC differentiation, and experimental reagents

CT26, 4T1, HEK293, and EMT6 cells were originally acquired from American Type Culture Collection, authenticated by analysis of short tandem repeats, and tested to ensure no presence of mycoplasma within 3 mo of use. U20S cells were kindly provided by the Walter Lab of the University of California, San Francisco. Cells were grown in RPMI1640 media supplemented with 10% FBS (Sigma-Aldrich), 2 mM glutaMAX (Gibco), 100 U/ml penicillin (Gibco), and 100 μg/ml streptomycin (Gibco). MEFs were obtained by dissecting 12-d-old embryos after removal of liver and head section. Remaining tissues were minced and digested in 0.1% trypsin/EDTA in PBS for 25 min in 37°C. Cells were washed twice in growth media and grown for 1 d. Experiments were performed with MEFs passaged less than three times.

For purification and differentiation of BMDCs, the femur and tibia bones of C57BL/6 mice were flushed with sterile PBS, and bone marrow cells were then cultured in RPMI1640 media as described above and further supplemented with 50 mM β2-mercaptoethanol (Sigma-Aldrich), 20 ng/ml GM-CSF (BioLegend), and 10 ng/ml IL-4 (BioLegend) for 9 d, with media being replenished every 3 d. Where indicated, Flt3L-BMDCs were similarly generated using media supplemented with 200 ng/ml recombinant mouse Flt3L (PeproTech; Brasel et al., 2000). After 9 d of culture, BMDCs were routinely verified to be >90% CD11c^+^ MHC-II^high^ by flow cytometry analysis.

Thapsigargin (Sigma-Aldrich) was used at 100 nM, tunicamycin (Sigma-Aldrich) was used at 5 μg/ml, MG132 (Sigma-Aldrich) was used at 5 μM, Amiloride (Sigma-Aldrich) was used at 10 μM, actinomycin D (Sigma-Aldrich) was used at 4 μg/ml, LPS (Sigma-Aldrich) was used at 10 μg/ml, and poly-I:C (Sigma-Aldrich) was used at 25 μg/ml. Ovalbumin (Sigma-Aldrich), EF-OVA (EndoFit; InvivoGen), SIINFEKL peptide (Sigma-Aldrich), and human CD4-Fc fusion protein (generated in-house at Genentech) were dissolved in PBS prior to pulsing and used at indicated concentrations.

The kinase-based IRE1α inhibitor G9668 (Harnoss et al., 2020; Harnoss et al., 2019; Harrington et al., 2015) was used as indicated.

For antigen uptake experiments, ovalbumin and CD4-Fc fusion proteins were labeled with allophycocyanin (APC) with the Lightning-Link Labeling Kit (Abcam).

For pulsing with tumor cell lysates, indicated cell lines were grown to confluence, suspended in sterile PBS, and subjected to five freeze–thaw cycles with liquid nitrogen and heating at 37°C. Cell lysates were then normalized by bicinchoninic acid protein concentration measurement (Thermo Fisher Scientific).

### In vitro characterization of small-molecule IRE1α inhibitor G9668

Potency of G9668 was analyzed in two assays of IRE1α activity, with dilutions covering a range of concentrations from 0.2 nM to 10 μM to determine half-maximal inhibitory concentration (IC_50_) values. Inhibition of RNase activity was assessed by the incubation of G9668 with IRE1α (Q470-L977) and 5′FAM-CAUGUCC**GC**AGC**G**CAUG-3′BHQ substrate. Substrate cleavage was monitored kinetically as an increase in fluorescence. Cellular activity was evaluated with the XBP1s-luciferase reporter assay in HEK293 cells stably transfected with the XBP1s-luciferse reporter construct. Briefly, cells were preincubated with G9668 for 2 h and subsequently stimulated with Tg (100 nM) for 6 h. IRE1α-mediated cleavage of the reporter led to luciferase expression, which was detected with the addition of luciferin substrate. Kinase selectivity of G9668 against a panel of 220 kinases was measured at a concentration of 1 μM with KinomeScan (DiscoverX). Fold selectivity was determined by IC_50_ measurement of competition by G9668 for binding of ATP to each specific kinase that showed significant inhibition by G9668 via KinomeScan.

### Generation of IRE1α KO syngeneic tumor cell lines

Individual IRE1α-specific single-guide RNAs (sgRNAs) were designed using a standard guide scaffold and CRISPR3. The gRNAs were cloned into pLKO_AIO_CMV_Cas9_mCherry, enabling co-expression of each sgRNA, Cas9, and an mCherry-based selection marker following transient transfection into target cells.

sgRNA target sequences used in this study are as follows: IRE1α gRNA1: 5′-TGT​TTG​TCT​CGA​CCC​TGG​A-3′; IRE1α gRNA2: 5′-GAG​GAC​GGG​CTC​CAT​CAA​G-3′; IRE1α gRNA3: 5′-GGA​GGC​CTG​AAC​CAA​TTC​T-3′; IRE1α gRNA4: 5′-ATG​TTA​TCG​ACC​TCC​TGA​C-3′.

Transfection was carried out with Lipofectamine 3000 (Thermo Fisher Scientific) according to the manufacturer’s protocol. At 24 h after transfection, cells were washed once in PBS and resuspended in PBS media containing 3% BSA Fraction V. The cell suspension was then filtered through a 35-mm membrane followed by immediate FACS sorting using the mCherry selection marker. Single-cell clones (*n* = 96) were plated and grown. Clones producing colonies were tested for proper IRE1α disruption by immunoblot.

### Immunoblot analysis

Cells were lysed in PBS solution supplemented with 1X radioimmunoprecipitation assay buffer (Millipore) and 2X Halt protease-phosphatase inhibitor cocktail (Thermo Fisher Scientific). Upon clearance, samples were analyzed by SDS-PAGE, electrotransferred to nitrocellulose membranes (Invitrogen), and blocked by 5% powdered milk in PBSt (PBS supplemented with 0.1% tween) solution. Development was conducted with ECL reagent (Thermo Fisher Scientific) and imaging was performed with the ChemiDoc ZRS+ imager (Bio-Rad). Antibodies used for Western blot analysis include IRE1α (3294, rabbit; Cell Signaling Technology), β-actin (5125, rabbit; Cell Signaling), GAPDH (97166, mouse; Cell Signaling), ATF6 (66563-1-Ig, mouse; ProteinTech), TAP1 (12341, rabbit; Cell Signaling), CHOP (2895, mouse; Cell Signaling), ATF4 (11815, rabbit; Cell Signaling), ovalbumin (P1-196, rabbit; Thermo Fisher Scientific), human Fc (ab977225, rabbit; Abcam), Myc tag (2272, rabbit; Cell Signaling), and biotin (5597, rabbit; Cell Signaling). IRE1α lumenal domain (mouse), XBP1s (rabbit), and pIRE1α (rabbit) antibodies were generated at Genentech. Secondary antibodies used were for mouse (715-035-150; The Jackson Laboratory) and rabbit (711-035-152; The Jackson Laboratory).

### Generation of TAP1 KO BMDCs

Bone marrow cells were purified from Cas9-expressing C57BL/6 mice as described above, subjected to red blood cell lysis with ACK lysis buffer, and electroporated with P3 Primary Cell 4D-Nucleaofactor X-kit (V4XP-3032; Lonza), as previously described (Freund et al., 2020). Once re-suspended in P3 buffer, cells were added to a Cas9–RNP complex (IDT) containing non-targeting or TAP1-targeting sgRNAs (IDT). The sequences of TAP1-targeting sgRNA included: sgRNA A: 5′-GCG​GCA​CCT​CGG​GAA​CCA​AC-3′, sgRNA B: 5′-TAA​CTG​ATA​GCG​AAG​GCA​TC-3′, and sgRNA C: 5′-ACG​GCC​GTG​CAT​GTG​TCC​CA-3′. These sgRNAs were used in combination. Bone marrow cells were then transfected with the appropriate program and matured for 9 d similar to all other BMDC cultures.

### In vitro IRE1α LD binding assays

For experiments testing ovalbumin binding, a human LD-Fc was used at indicated concentrations. LD-Fc is comprised of amino acids M1-D443 of IRE1α fused C-terminally to a linker (GRAQVTDKAARSTL) followed by the human IgG1 hinge and Fc portion. Ovalbumin (Sigma-Aldrich) was dissolved in sterile PBS and used in native state or denatured as indicated by incubation at 95°C buffer for 10 min.

For plate-based binding experiments, native or heat-denatured ovalbumin at indicated concentrations was bound to a flat-bottom 96-well plate (Corning Inc.) with coating buffer (BioLegend), washed with PBSt, blocked with 1% BSA in PBS, and subsequently incubated with IRE1α LD-Fc (10 μg/ml) in binding buffer composed of PBS with 20 mM Hepes, 100 mM KOAc, and 0.2% tween-20 for 2 h at room temperature. Plates were washed again with PBSt and incubated with an anti-human Fc HRP-conjugated antibody (ab977225; Abcam). Development was performed with 3,3′,5,5′-Tetramethylbenzidine solution and terminated with Stop solution (BioLegend). Readings were taken with a SpectraMax M2 spectrometer (Molecular Devices).

For co-immunoprecipitation experiments, IRE1α LD-Fc (10 μg/ml) and ovalbumin (indicated concentrations) were co-incubated in binding buffer (as described above) for 2 h and then immunoprecipitated with anti-IRE1α LD antibody (made in-house at Genentech), conjugated to sepharose beads, overnight at 4°C. Beads were subsequently washed four times with lysis buffer and boiled in SDS sample buffer for 10 min. Samples were then analyzed by SDS-PAGE followed by immunoblot.

Peptide arrays were produced by the Massachusetts Institute of Technology Biopolymers Laboratory. The tiling arrays were composed of 18-mer peptides spanning the ovalbumin or GP70 sequences and overlapped by three amino acids. The arrays were incubated in methanol for 10 min and then in binding buffer (50 mM Tris, pH 7, 250 mM NaCl, 10% glycerol, 2 mM DTT) for three 10-min wash cycles. The arrays were then incubated for 1 h at room temperature with 500 nM IRE1α LD-Fc and washed again for three 10-min cycles in binding buffer to remove any unbound LD-Fc. Using a semi-dry transfer apparatus, bound IRE1α LD-Fc was transferred after washing to a polyvinylidene fluoride membrane and detection was carried out with an anti-human Fc antibody (ab977225; Abcam), ECL solution (Thermo Fisher Scientific), and the ChemiDoc ZRS+ imager (Bio-Rad). To measure binding of IRE1α LD-Fc to each peptide, images containing developed membranes were quantified with ImageJ software (version 2.0.0). Pixel intensity was determined for all spots containing peptides, with background subtracted for spots containing no peptides. Peptides were considered to bind IRE1α LD-Fc if spot intensity was above the average of all array peptides.

For binding assays of biotin-tagged peptides, we generated a FLAG-tagged IRE1α LD (LD-FLAG) comprised of amino acids M1-D443 of IRE1α fused C-terminally to a linker (GNS) followed by a Flag tag (DYKDDDDK). LD-FLAG was incubated with synthetic N-terminal biotin-tagged peptides derived from ovalbumin in binding buffer (as described above) for 1.5 h, cross-linked by 25 μM disuccinimidyl suberate (Thermo Fisher Scientific) for 1 h and subsequently incubated with 50 μM Tris (pH 7.5) for 15 min to quench cross-linking. Samples were then analyzed by SDS-PAGE followed by anti-biotin immunoblot.

### Transfection of ovalbumin-derived peptides and co-immunoprecipitation of Myc-tagged peptides with IRE1α

U20S or HEK293 cells were transfected utilizing the TransIT-XL reagent (Mirus Bio) and PRK-TK-Neo plasmids encoding for ovalbumin-derived peptides with a signal sequence (MGGTAARLGAVILFVVIVGLHGVRG, based on the signal sequence of Human Herpes Virus 1 Glycoprotein D, with an added lysine residue to allow signal sequence processing upon translation), a flexible linker (DLGSSG) prior to the peptide sequence, and an N-terminal Myc tag (EQKLISEE). For immunoprecipitation or RT-qPCR analysis experiments, cells were harvested 48 h after transfection, washed twice with cold PBS, and harvested in cold PBS with protease inhibitor (Roche, Basel, Switzerland). Cells were lysed for 20 min on ice in lysis buffer (30 mM Tris, pH 7.5, 150 mM NaCl, 1% Triton X-100). The lysates were cleared by centrifugation at 14,000 rpm for 10 min and then incubated with anti-Myc (Thermo Fisher Scientific) antibody-conjugated sepharose beads overnight at 4°C. Beads were subsequently washed four times with lysis buffer and boiled in SDS sample buffer for 10 min. Samples were then analyzed by SDS-PAGE followed by immunoblot.

### RT-qPCR assay of transcript abundance

For RT-qPCR analysis, RNA was purified from sorted splenic DCs, BMDCs, HEK293, or U20S cells with the RNeasy Mini-kit (Qiagen) and quantified with a NanoDrop 8000 spectrophotometer (Thermo Fisher Scientific). Similar amounts of RNA were reverse transcribed and amplified using the Taqman RNA-to-CT 1-Step kit (Applied Biosystems). The following Taqman probes were used for HEK293 or U20S-derived RNA: XBP1s (Hs03929085), XBP1u (Hs028565596), CD59 (Hs00174141), DGAT2 (Hs01045913), CHOP (Hs00358796), and GAPDH (Hs02758991; Thermo Fisher Scientific). The following Taqman probes were used for splenic DC- or BMDC-derived RNA: H-2K (Mm01612247), CD59 (Mm00483149), DGAT2 (Mm0049536), BLOC1S1 (Mm00497168), RNF213 (Mm01248886), IRF7 (Mm00516793), β−2M (Mm00437762), TAP1 (Mm00443188), TAPBP (Mm00493417), ERAP1 (Mm00472842), and GAPDH (Mm99999915; Thermo Fisher Scientific). Assays were performed with the ViiA 7 (Applied Biosystems) system.

### In vitro degradation of MHC-I heavy-chain transcripts

To search for IRE1α cleavage sites within MHC-I heavy-chain mRNAs, sequences were loaded onto A Plasmid Editor software and subjected to a search function for consensus GCAG locations. The location most likely to provide a stable stem-loop structure within each transcript was then chosen.

To determine cleavage by IRE1α, T7 RNA transcripts were synthesized based on cDNA templates of H-2K (#OMu17935; GenScript), H-2D (#MC208623; Origene), and H-2L (#MC227254; Origene). Amplification of cDNA was conducted using T7 forward primers, and cDNA-based RNA was generated using HiScribe T7 Quick High-Yield RNA Synthesis kit (New England Biolabs). T7 RNA (1 μg) was digested at room temperature by IRE1α recombinant kinase-RNase protein (1 μg) for 15 min in RNA cleavage buffer (Hepes, pH 7.5, 20 mM, KOAc 50 mM, MGAc 1 mM, Tritox X-100 0.05%). The digestion was terminated by addition of formamide (97%), and digestion products were then exposed to 70°C temperature to linearize the RNA. Immediately after linearization, samples were placed on ice for 5 min and then run on a 3% agarose gel. Gels were visualized by a ChemiDoc ZRS+ imager (Bio-Rad).

### Ex vivo T cell activation and cross-presentation experiments

For ex vivo T cell activation experiments, mice were euthanized and spleens were removed and mechanically disrupted with a GentleMacs tissue dissociator (Miltenyi Biotec, Inc.). Total spleen cells were washed with sterile PBS, counted, and CD8^+^ or CD4^+^ T cells were magnetically separated with CD8^+^ or CD4^+^ separation kits (19853 and 19852, respectively; Stemcell Technologies).

For CD3/CD28-mediated activation, ultra low-endotoxin, azide-free plate-bound anti-mouse CD3 (100223; BioLegend) was used at 2 μg/ml, and soluble anti-mouse CD28 (102116; BioLegend) was used at 8 μg/ml. T cells were incubated for 72 h prior to flow cytometry analysis or Cell Titer Glo (G7570; Promega) analysis.

For antigen cross-presentation assays, 2 × 10^4^ BMDCs were plated, activated with LPS (10 μg/ml) for 2 h, and pulsed with SIINFEKL (100 nM), ovalbumin, EF-OVA, or CT26 lysate (all at 250 μg/ml) overnight. BMDCs were then washed with media, and 2 × 10^5^ CD8^+^ or CD4^+^ T cells pre-stained with Celltrace Violet Cell Proliferation reagent (C34557; Thermo Fisher Scientific) were added and co-incubated for 72 h. Proliferation was then determined by loss of Celltrace Violet signal in propidium iodide (Sigma-Aldrich)–negative, viable CD8^+^, or CD4^+^ T cells after co-incubation.

### Mouse strains and in vivo tumor growth studies

All animal procedures were approved and conformed to guidelines established by the Institutional Animal Care and Use Committee of Genentech and were carried out in facilities accredited by the Association for the Assessment and Accreditation of Laboratory Animal Care. In all in vivo studies, tumor size and body weight were measured twice per week. Subcutaneous and mammary fat pad tumor volumes were measured in two dimensions (length and width) using Ultra Cal-IV calipers (model 54 − 10 − 111; Fred V. Fowler Co.). The tumor volume was calculated using the following formula: tumor size (mm^3^) = (longer measurement × shorter measurement^2^) × 0.5.

TGI, as a percentage of vehicle, was calculated as the percent difference between the daily average area under the tumor volume–time curve (AUC) of treatment and control group fits on the original untransformed scale over the same time period using the following formula: %TGI = (1 − [(AUC/d) treatment (AUC/d) vehicle]) × 100.

C57BL/6 and Balb/C mice were acquired from The Jackson Laboratory or Charles River laboratories, and MyD88 KO mice were acquired from Charles River Laboratories. For TAP1 KO experiments, in house-generated Cas9-expressing C57BL/6 mice were used.

For CT26 and 4T1 tumor studies, mice were inoculated subcutaneously (s.c.) on the right flank. 1 × 10^5^ CT26 or 4T1 cells were counted and suspended in HBSS (Gibco) and admixed with 50% Matrigel (BD) to a final volume of 100 μl. For EMT6 tumor studies, an identical number of cells was prepared similarly and inoculated into the mammary fat pad.

For in vivo studies, 7 d after tumor-cell inoculation, animals were randomized into groups receiving vehicle control (50% PEG400, 40% water, 10% DMSO) or G9668 (250 mg/kg) compound bidaily (BID) by oral gavage (p.o.). For the EMT6 combination studies, mice were randomized into four groups, with groups similarly receiving vehicle or G9668 in combination with anti-GP120 control antibody or 6E11 anti–PD-L1 antibody (both with LALAPG Fc alterations, dissolved in PBS, at 10 mg/kg i.v. for the first dose and 5 mg/kg i.p. biweekly [BIW] thereafter).

### Flow cytometry and cell sorting

For in vivo experiments, tumors were excised after euthanasia, mechanically disrupted by a GentleMacs tissue dissociator (Miltenyi Biotec, Inc.), and enzymatically digested by dispase (80 μg/ml; Life Technologies), Collagenase P (20 μg/ml; Roche), and DNAse I (10 μg/ml; Roche). For cytokine staining assays, cells were re-suspended in RPMI1640 growth media supplemented with T cell stimulation cocktail (4975-03; eBioscience Inc.) and brefeldin A (BioLegend) and incubated at 37°C for 4 h.

For flow cytometry analysis assays, samples were re-suspended in FACS buffer (0.5% BSA, 0.05% azide), blocked with anti-CD16/32 blocking antibodies (101302; BioLegend) for 20 min in 4°C, and then incubated with fluorescently labeled antibodies for a further 20 min in 4°C. The following dyes were used: propidium iodide (Sigma-Aldrich), Celltrace Violet (C34557; Invitrogen), and Live/Dead Staining Kit (L10119; Invitrogen). The following antibodies were used: Perforin (PE; 12-9392-82; eBiosciences), ki67 (BV421, 652411), CD8 (PE-Cy7, 100722), CD4 (APC-Cy7, 100526), CD3 (APC, 100236), F4/80 (APC-Cy7, 123118), MHC-I (FITC, 125508), MHC-II (BV605, 107639), CD11c (BV711, 117349), CD11b (APC, 101212), XCR1 (BV421, 148216), CD103 (AF488, 121408), granzyme B (FITC, 515403), CD59 (PE, 143103), PD-1 (BV605, 135220), CD44 (BV711, 103057), CD69 (PE-Cy7, 104512), IFN-γ (APC, 505810; all from BioLegend). The Foxp3 Fix/Perm Kit was used for intracellular staining (421403; BioLegend). GP70 tetramers were generated at Genentech, as described (Vormehr et al., 2020).

Samples were read in a BD Symphony cell analyzer (BD), and data were analyzed in FlowJo Software (FlowJo 10.2; FlowJo LLC). For cell sorting, a BD FACSAria II (BD) was utilized.

For splenic DC experiments, C57BL/6 mice were injected once with Flt3L-Fc (produced at Genentech, i.v., 10 mg/kg; Tu et al., 2014). After 8 d, mice were treated with G9668 for 24 h per os gavage (250 mg/kg, BID) and injected i.v. with DEC-OVA (produced at Genentech, 2.5 mg/kg) for 2.5 h prior to take-down (Boscardin et al., 2006; Hawiger et al., 2001). Spleens were disrupted mechanically with GentleMacs tissue dissociator (Miltenyi Biotec, Inc.), as described, for T cell separation, counted, and stained as described for flow cytometry analysis. Cell sorting was then performed with BD FACSAria (BD). Splenic DCs were sorted as viable CD11c^+^ MHC-II^high^ F4/80^−^ and separated into the cDC1 (CD103^+^ XCR1^+^ CD11b^−^) and the cDC2 (CD103^−^ XCR1^−^ CD11b^+^) subpopulations.

### scRNAseq

For scRNAseq, libraries were generated using Chromium Single Cell 5′ Library & Gel Bead kit (1000006; 10× Genomics) from 2.5 × 10^4^ viable CD45^+^ cells sorted from 4T1 tumors.

### Statistical analysis

All values were represented as arithmetic mean ± SD. Statistical analysis was performed by unpaired, two-tailed *t* test, or one-way ANOVA. A resulting P < 0.05 was considered significant. All analyses were performed with the GraphPad Prism 7 software (GraphPad Software, Inc.).

### Online supplemental material

Fig. S1 contains supporting information regarding ovalbumin-induced IRE1α activation in BMDCs as well as structure and specificity characteristics of G9668. Fig. S2 presents supporting data for peptide arrays used in the study and sequences of peptides used in in vitro and in vivo IRE1α binding assays. Fig. S3 presents supporting data from antigen cross-presentation and CD8^+^ T cell proliferation assays, in vitro MHC-I heavy-chain mRNA degradation experiments, and ex vivo and in vivo RT-qPCR experiments with antigen-pulsed BMDCs and splenic DCs. Fig. S4 contains data from in vivo tumor experiments with the CT26 model, displaying tumor growth and IRE1α activation in parental and *IRE1α* KO tumors. Fig. S5 contains data from in vivo tumor experiments with the 4T1 model, displaying tumor growth and IRE1α activation in parental and *IRE1α* KO tumors, along with further data from scRNAseq experiments. Fig. S6 displays data from in vivo experiments with the EMT6 model, including tumor growth and IRE1α activation in parental and *IRE1α* KO tumors, as well as in mice bearing parental EMT6 tumors treated with G9668, anti–PD-L1, or the combination of both agents.

## Supplementary Material

SourceData F1 contains original blots for Fig. 1.

SourceData F2 contains original blots for Fig. 2.

SourceData F4 contains original blots for Fig. 4.

SourceData FS1 contains original blots for Fig. S1.

SourceData FS2 contains original blots for Fig. S2.

SourceData FS4 contains original blots for Fig. S4.

SourceData FS5 contains original blots for Fig. S5.

SourceData FS6 contains original blots for Fig. S6.
