# Supplementary material for: Antigen-derived peptides engage the ER stress sensor IRE1α to curb dendritic cell cross-presentation
Source: J Cell Biol. 2022 Apr 21;221(6):e202111068. doi: 10.1083/jcb.202111068 (PMC9036094; doi:10.1083/jcb.202111068)
Supplement: SourceData F1 — contains original blots for Fig. 1. [file JCB_202111068_SourceDataF1.pdf]

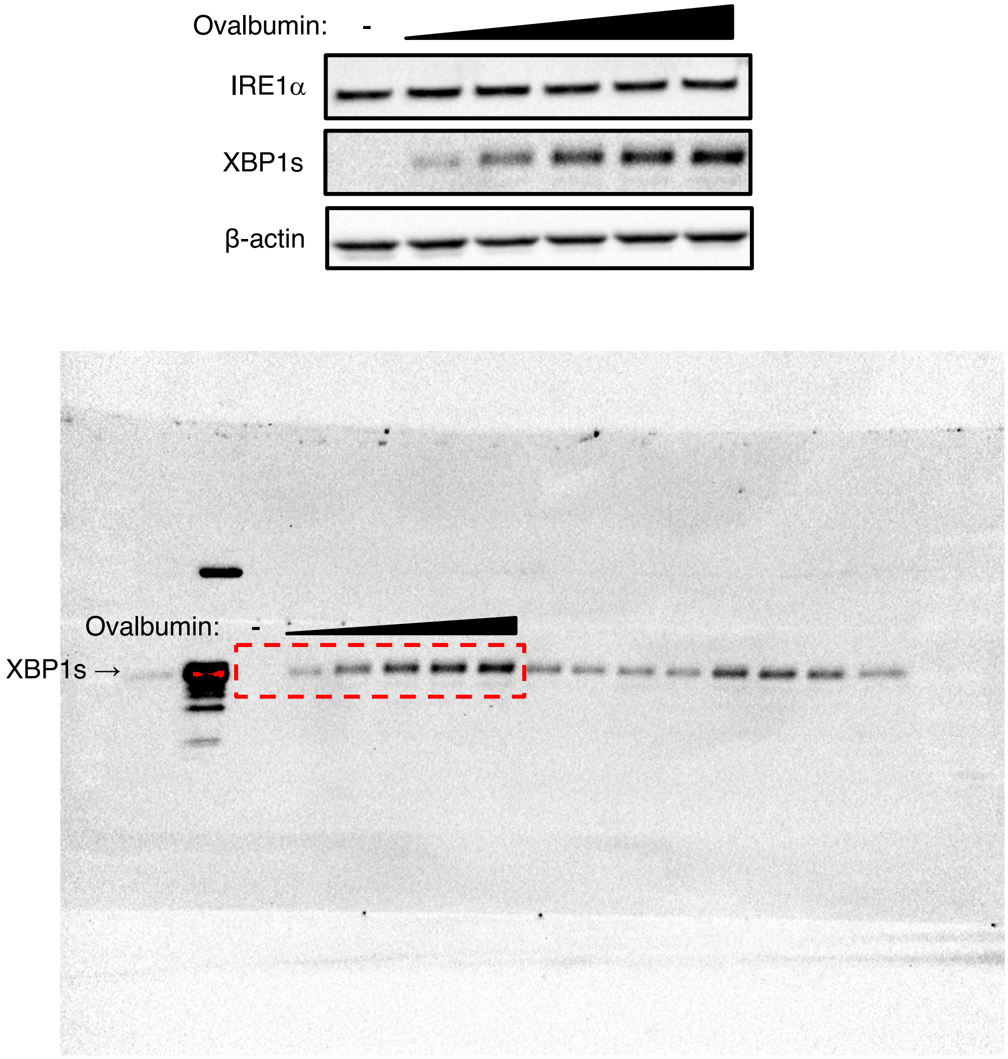

Figure 1. Antigen pulsing of BMDCs activates IRE1α. (**A)** BMDCs were pulsed with ovalbumin (starting at 62.5 µg/ml and sequentially doubled) and analyzed by immunoblot (IB) for the indicated markers.

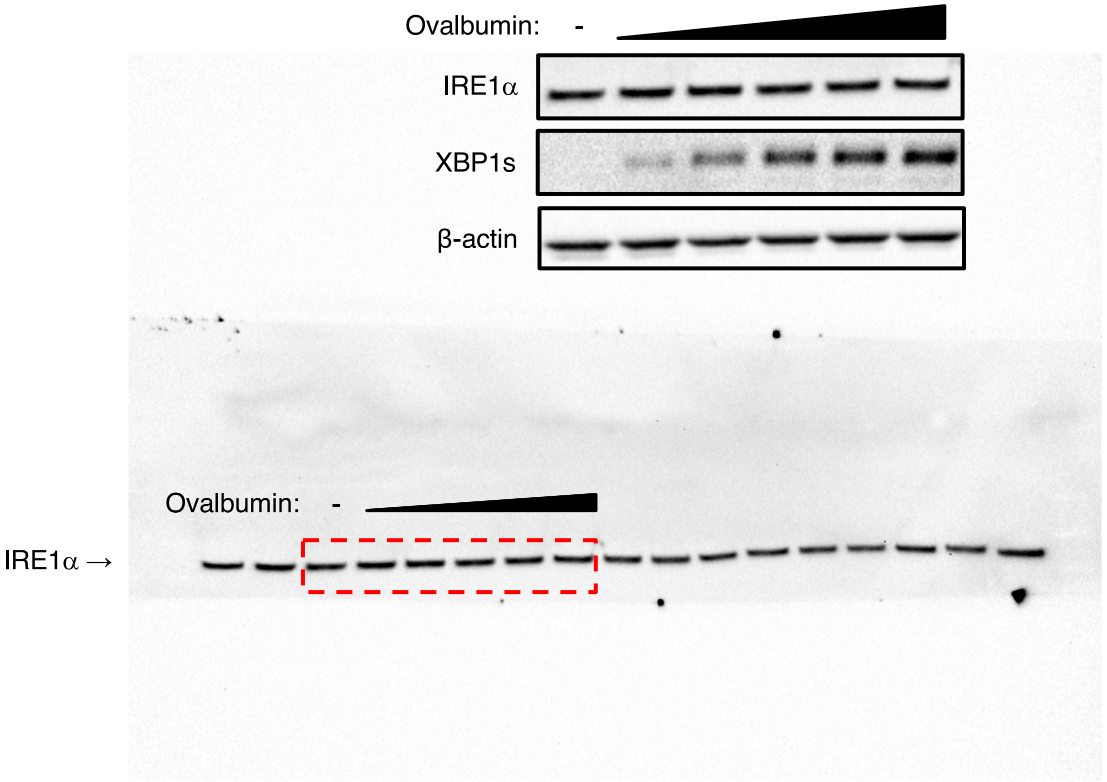

Figure 1. Antigen pulsing of BMDCs activates IRE1α. (**A)** BMDCs were pulsed with ovalbumin (starting at 62.5 µg/ml and sequentially doubled) and analyzed by immunoblot (IB) for the indicated markers.

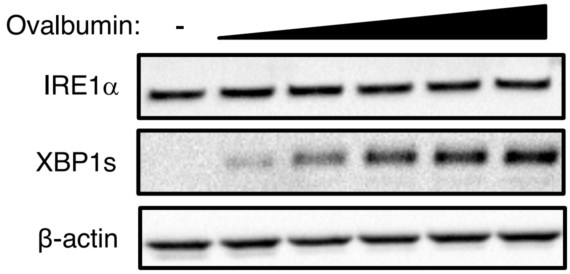

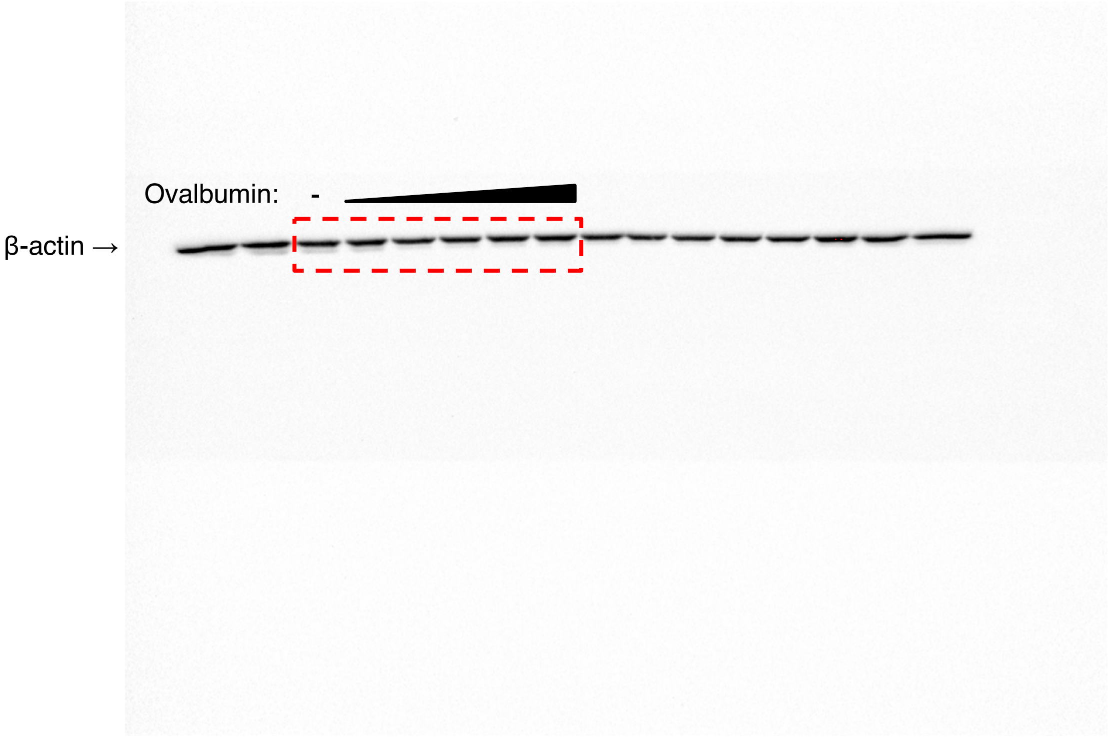

Figure 1. Antigen pulsing of BMDCs activates IRE1α. (**A)** BMDCs were pulsed with ovalbumin (starting at 62.5 µg/ml and sequentially doubled) and analyzed by immunoblot (IB) for the indicated markers.

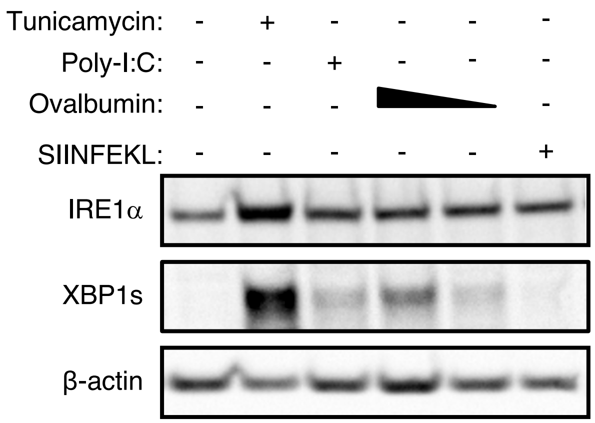

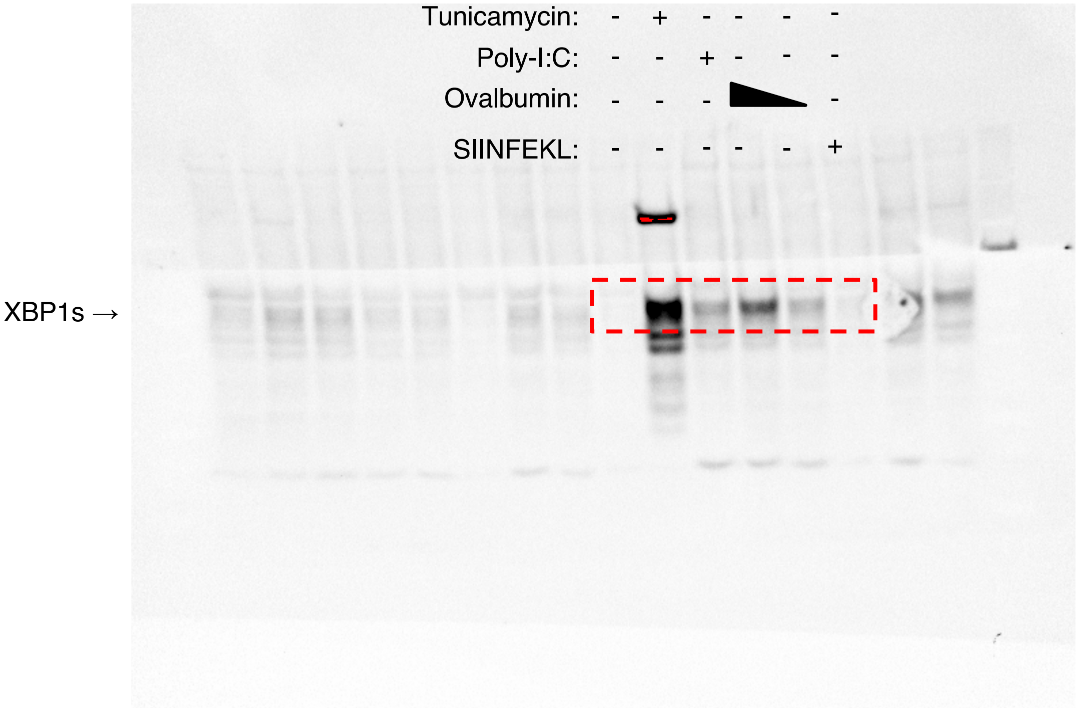

Figure 1. Antigen pulsing of BMDCs activates IRE1α. (B) BMDCs were pulsed with ovalbumin (starting at 500 µg/ml and sequentially halved) or SIINFEKL (1 µM), or stimulated with tunicamycin (1 µg/ml) or poly-I:C (25 µg/ml) for 4 hr, and analyzed by IB.

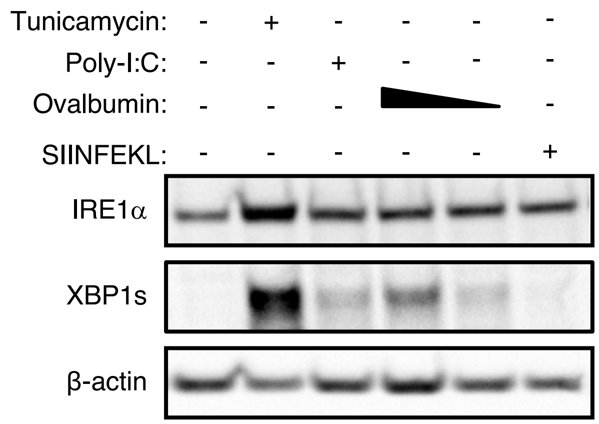

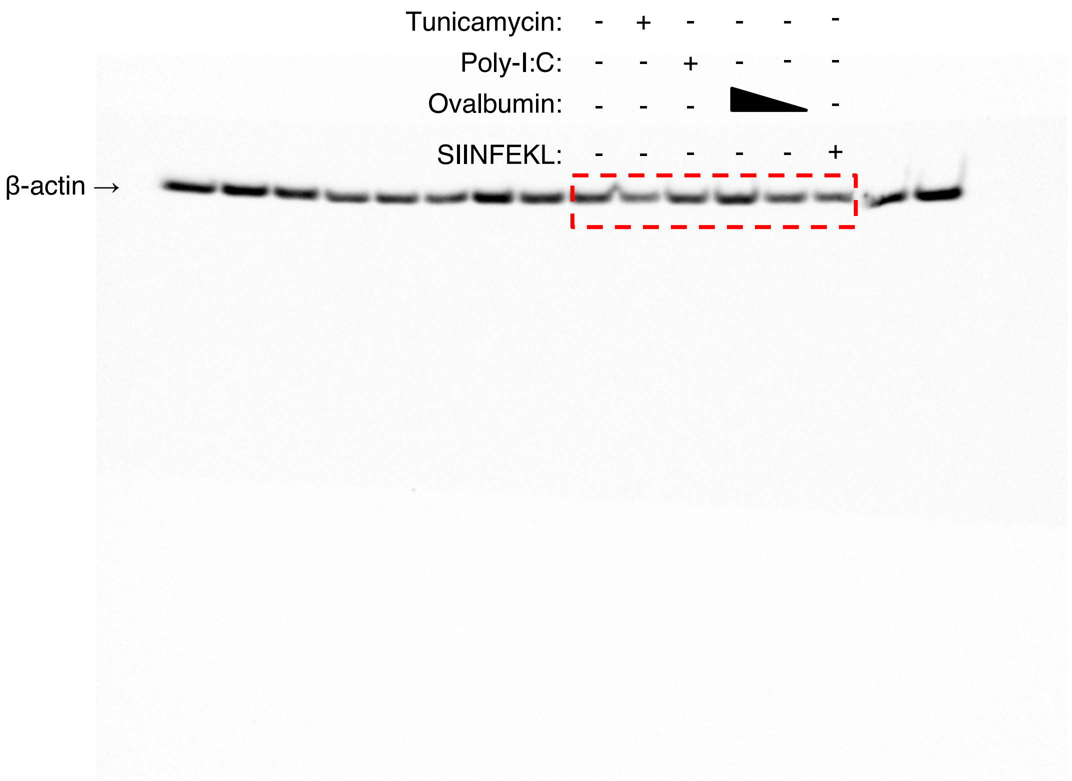

Figure 1. Antigen pulsing of BMDCs activates IRE1α. (B) BMDCs were pulsed with ovalbumin (starting at 500 μg/ml and sequentially halved) or SIINFEKL (1 μM), or stimulated with tunicamycin (1 μg/ml) or poly-I:C (25 μg/ml) for 4 hr, and analyzed by IB.

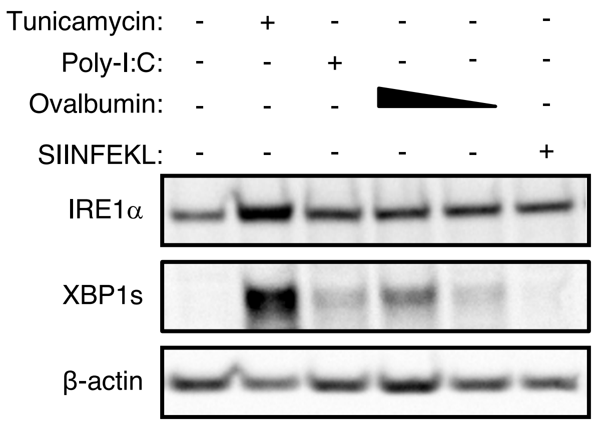

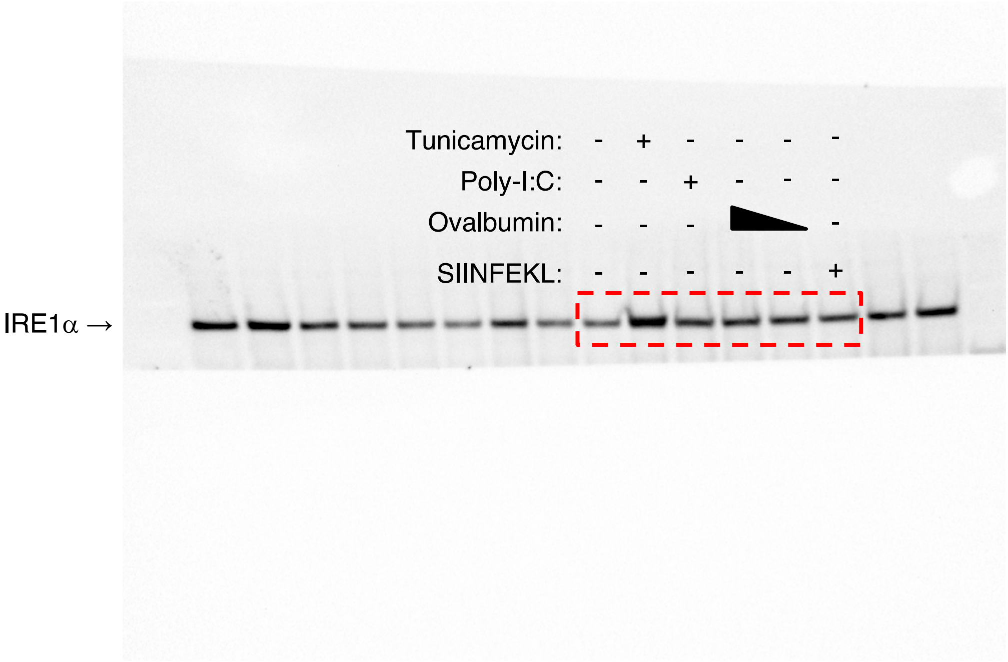

Figure 1. Antigen pulsing of BMDCs activates IRE1α. (B) BMDCs were pulsed with ovalbumin (starting at 500 µg/ml and sequentially halved) or SIINFEKL (1 µM), or stimulated with tunicamycin (1 µg/ml) or poly-I:C (25 µg/ml) for 4 hr, and analyzed by IB.

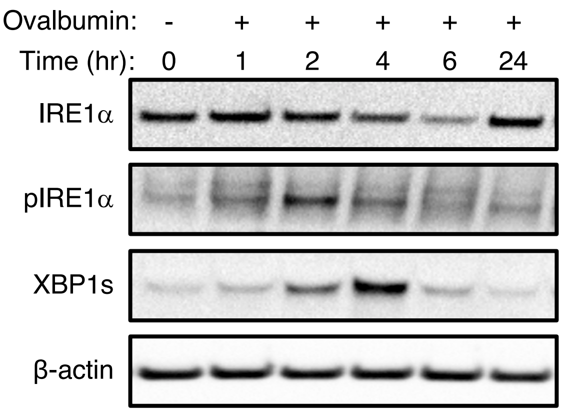

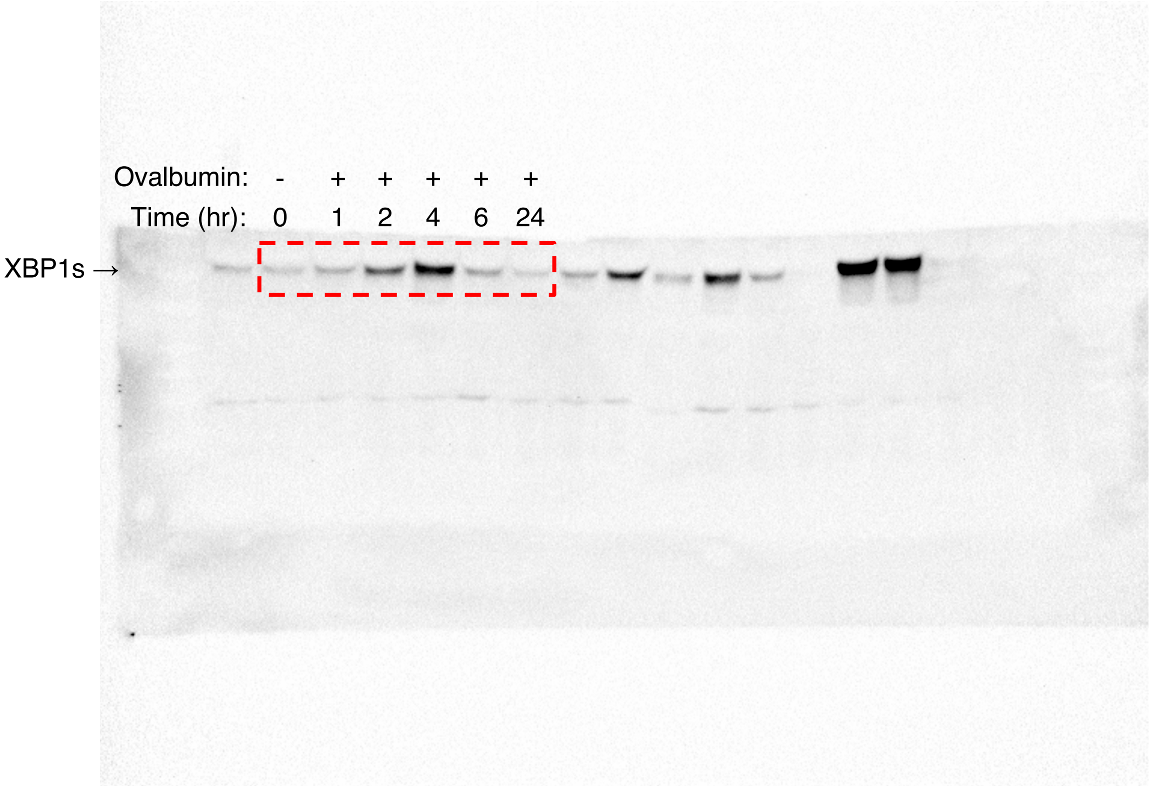

Figure 1. Antigen pulsing of BMDCs activates IRE1α. (C) BMDCs were pulsed with ovalbumin (500 µg/ml) for the indicated time and analyzed by IB.

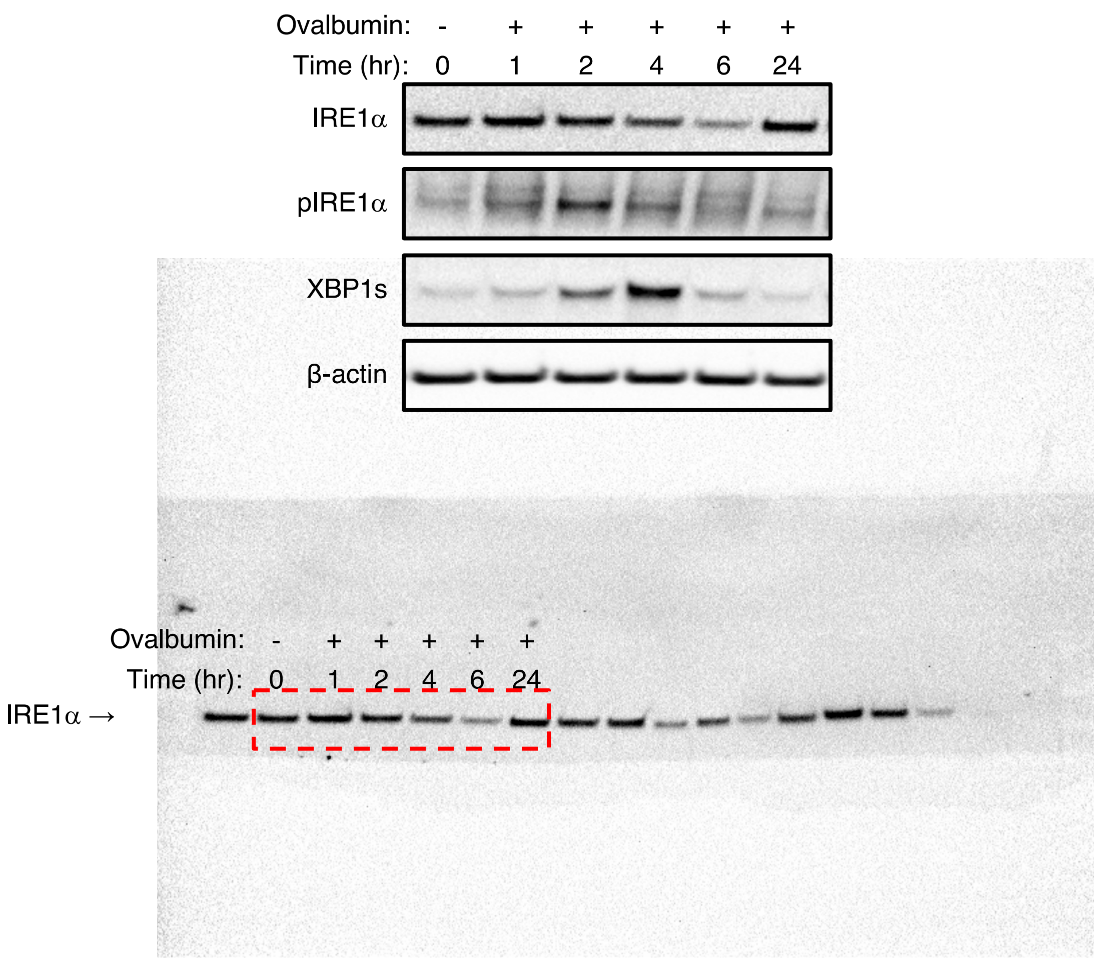

Figure 1. Antigen pulsing of BMDCs activates IRE1α. (C) BMDCs were pulsed with ovalbumin (500 µg/ml) for the indicated time and analyzed by IB.

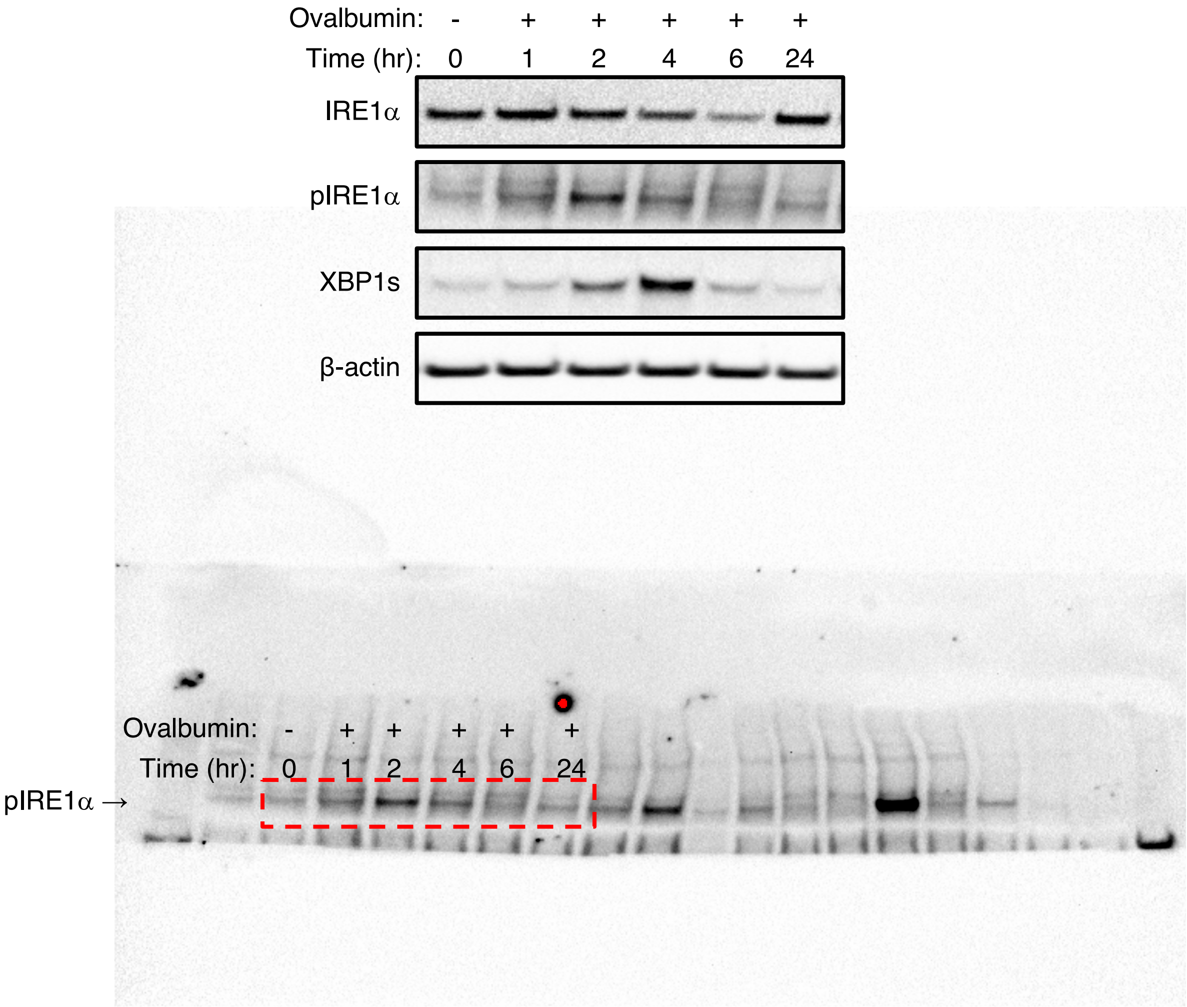

Figure 1. Antigen pulsing of BMDCs activates IRE1α. (C) BMDCs were pulsed with ovalbumin (500 µg/ml) for the indicated time and analyzed by IB.

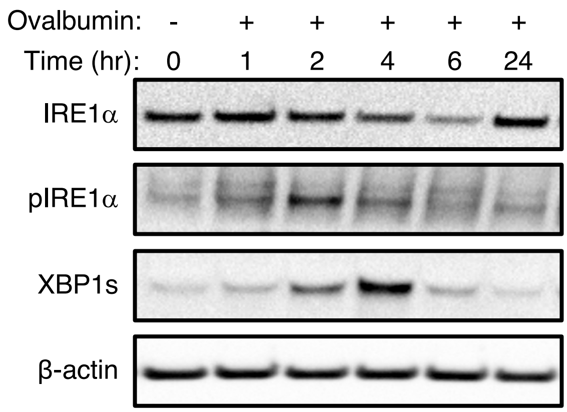

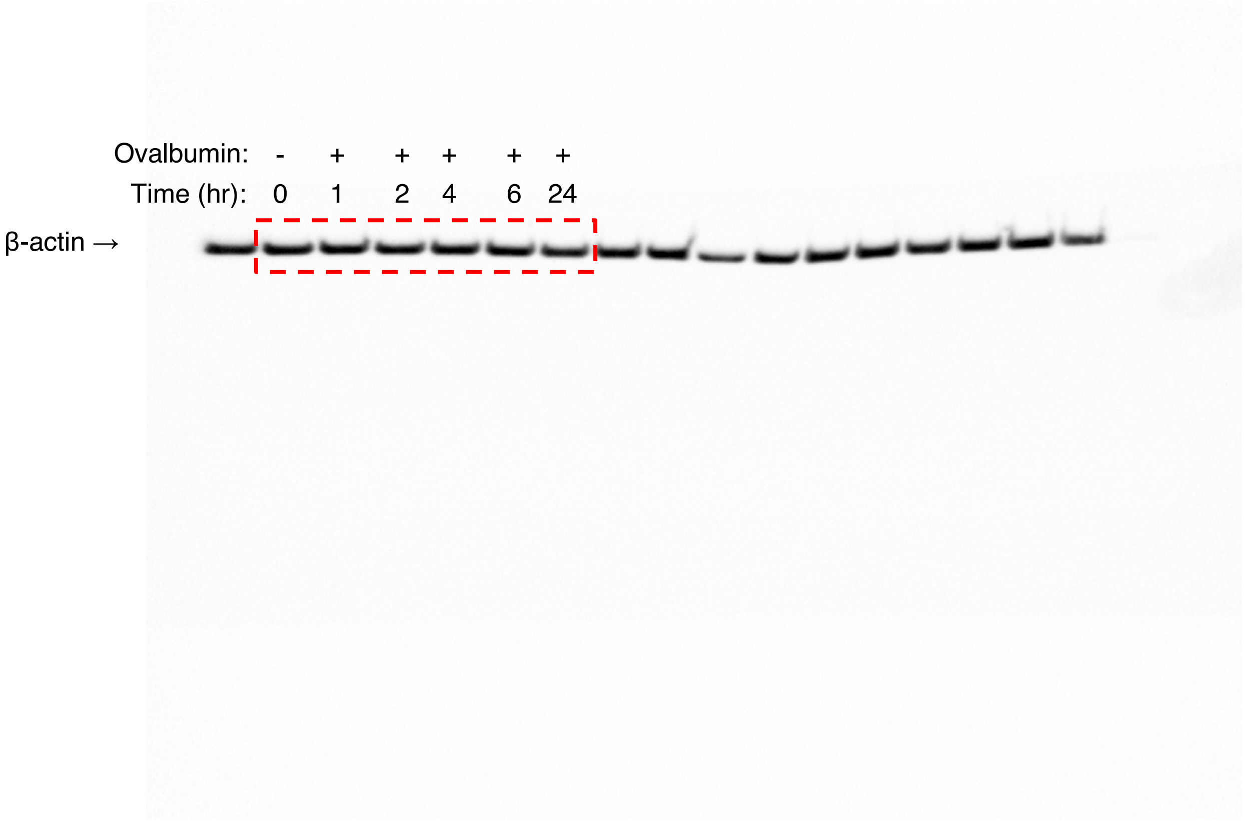

Figure 1. Antigen pulsing of BMDCs activates IRE1α. (C) BMDCs were pulsed with ovalbumin (500 µg/ml) for the indicated time and analyzed by IB.

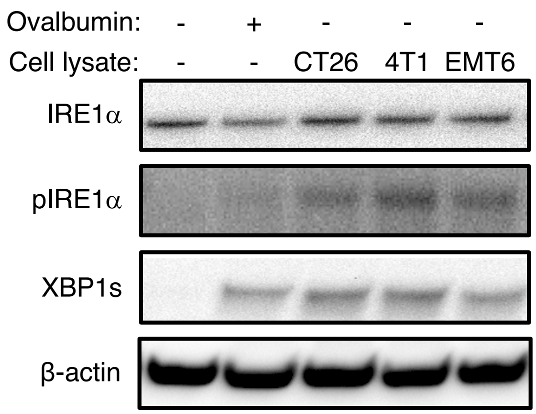

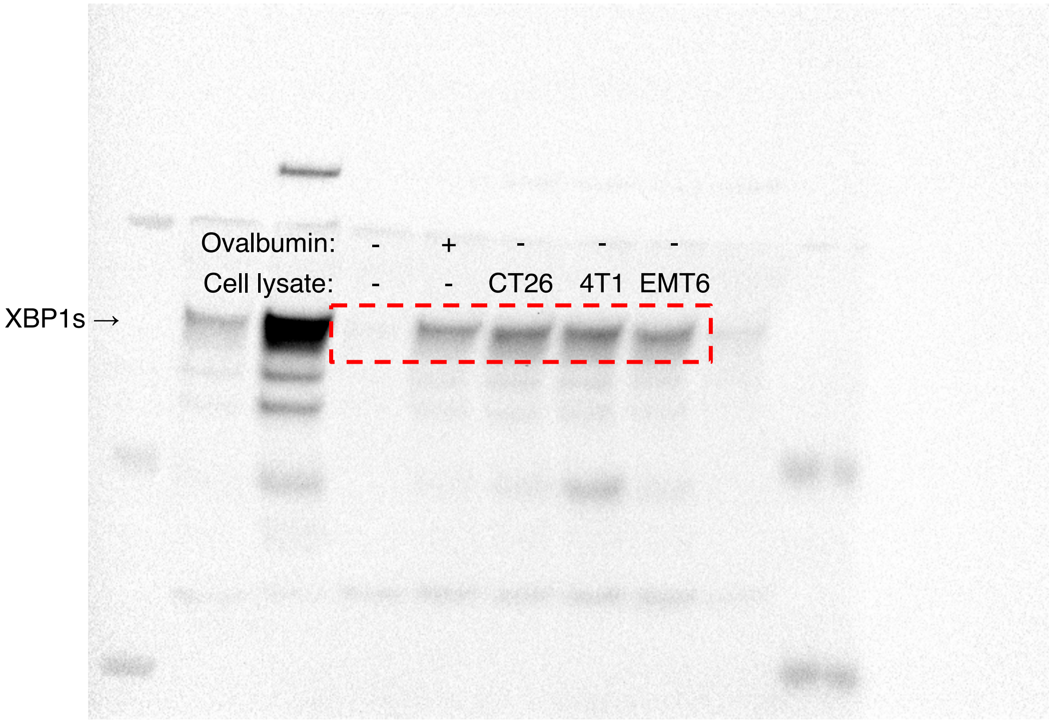

Figure 1. Antigen pulsing of BMDCs activates IRE1α. (D) BMDCs were pulsed for 4 hr with ovalbumin or lysates derived from the indicated cell lines (500 µg/ml protein) and analyzed by IB.

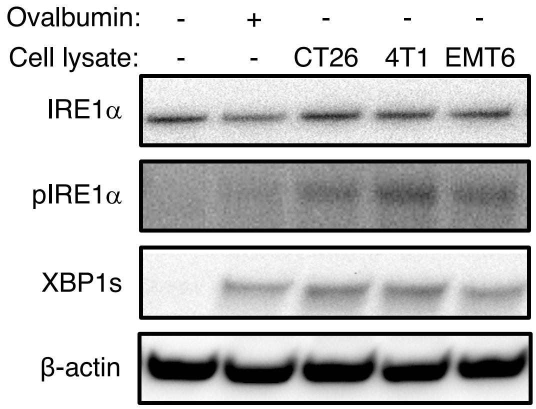

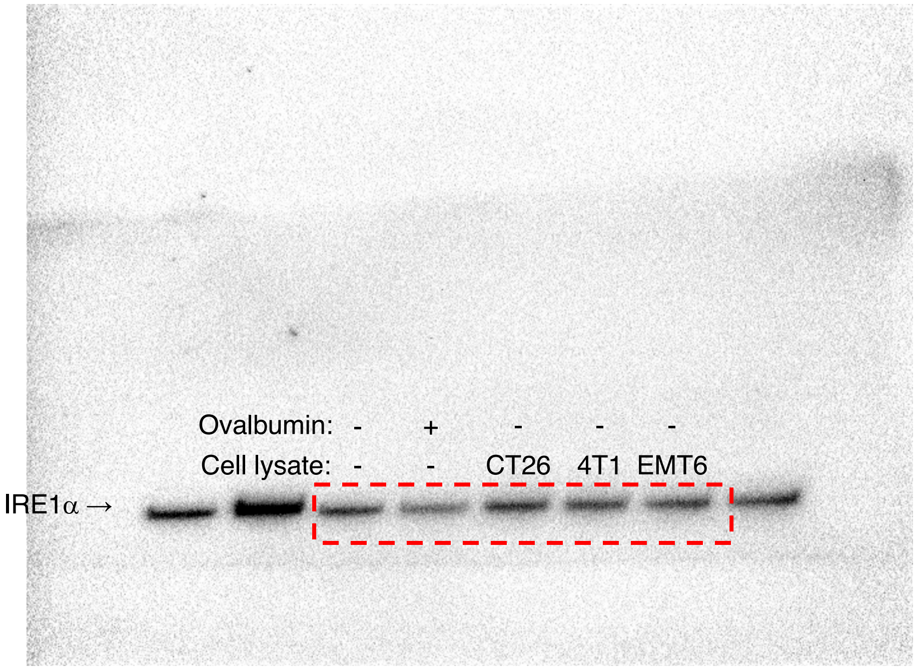

Figure 1. Antigen pulsing of BMDCs activates IRE1α. (D) BMDCs were pulsed for 4 hr with ovalbumin or lysates derived from the indicated cell lines (500 µg/ml protein) and analyzed by IB.

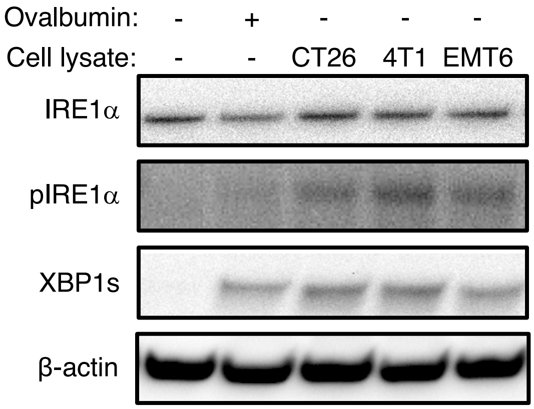

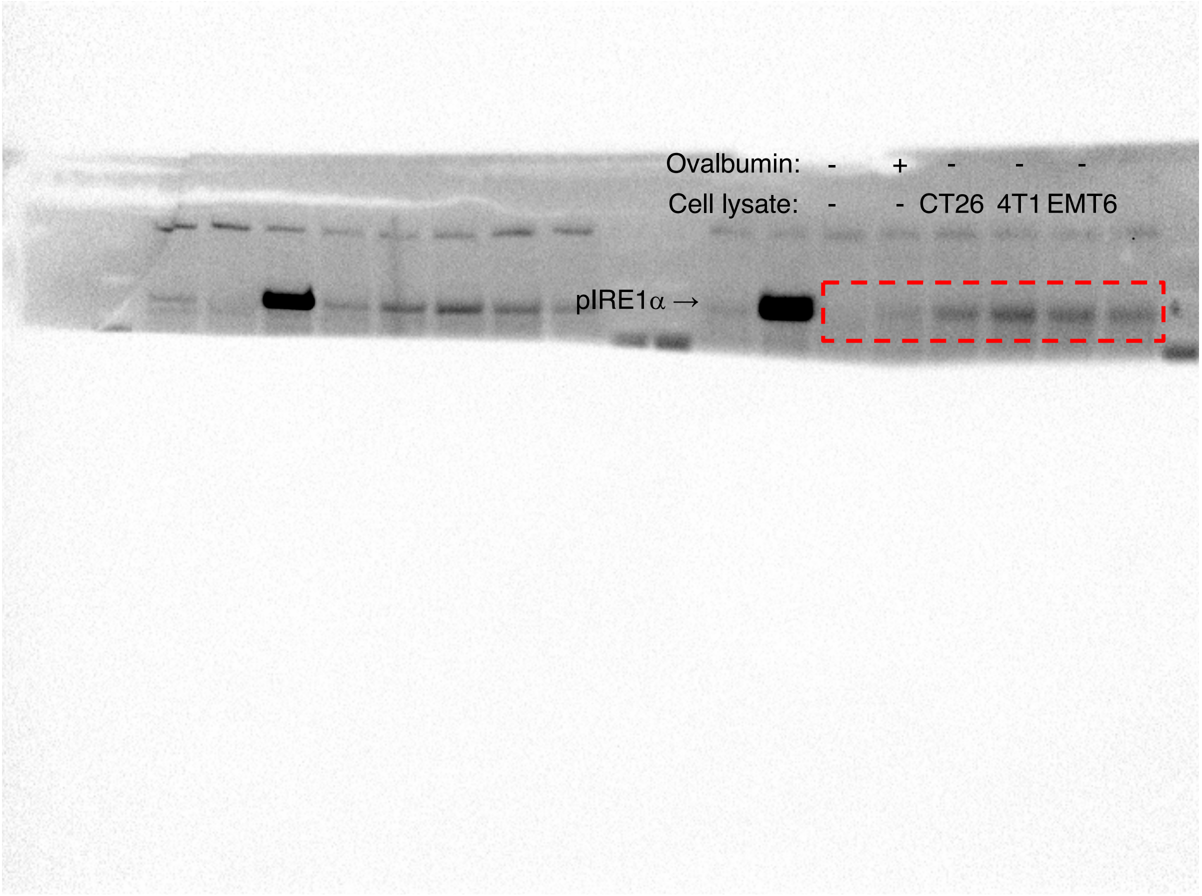

Figure 1. Antigen pulsing of BMDCs activates IRE1α. (D) BMDCs were pulsed for 4 hr with ovalbumin or lysates derived from the indicated cell lines (500 µg/ml protein) and analyzed by IB.

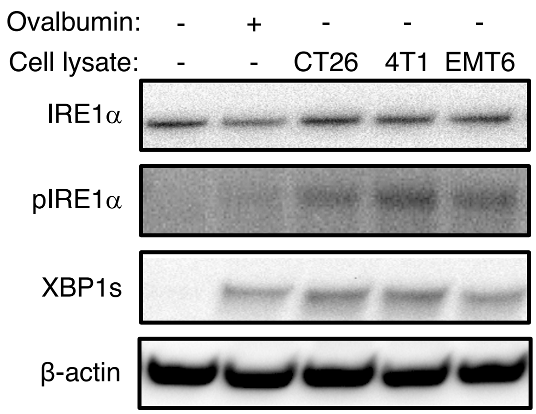

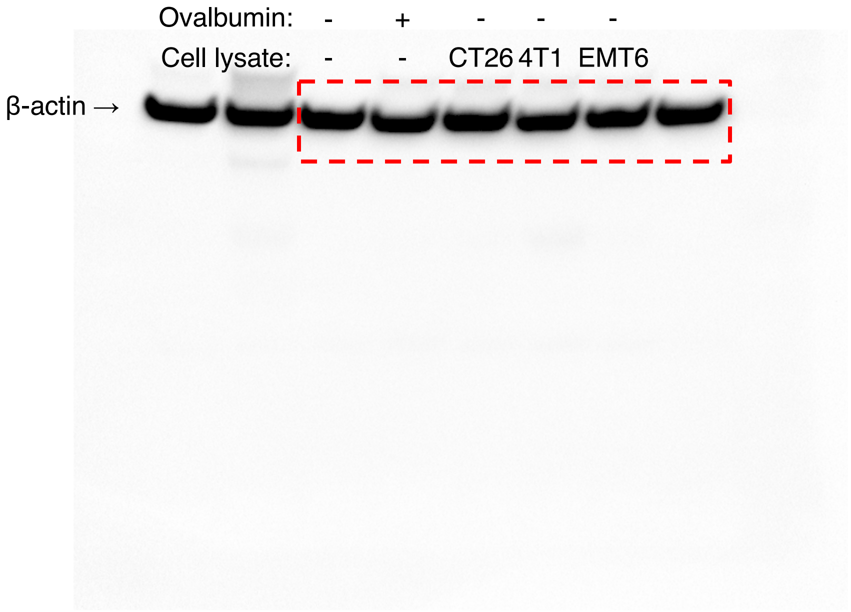

Figure 1. Antigen pulsing of BMDCs activates IRE1α. (D) BMDCs were pulsed for 4 hr with ovalbumin or lysates derived from the indicated cell lines (500 μg/ml protein) and analyzed by IB.

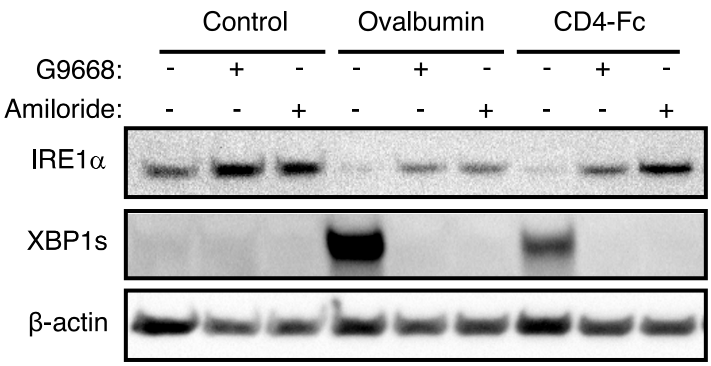

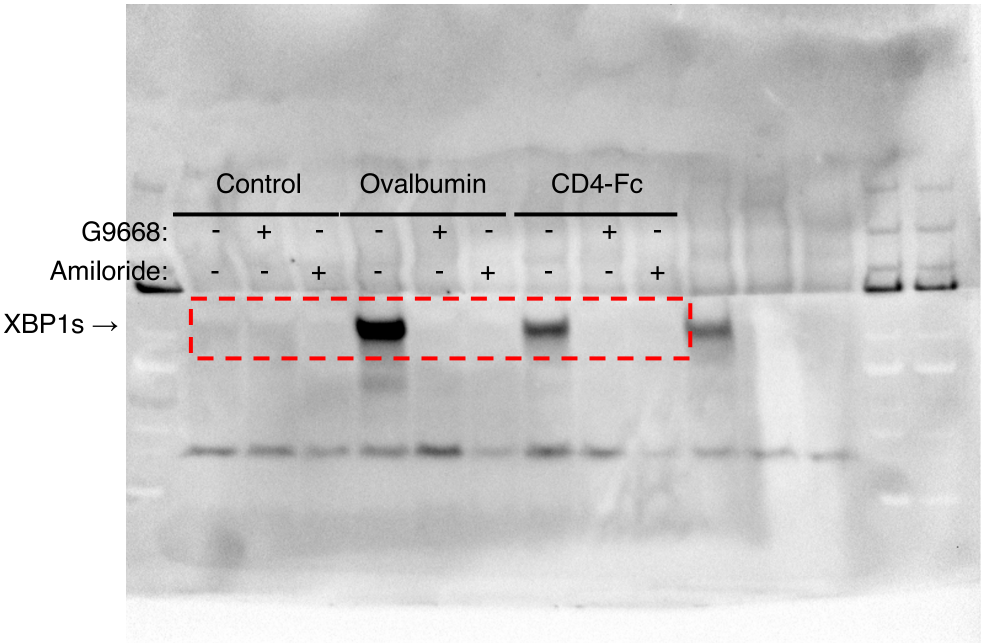

Figure 1. Antigen pulsing of BMDCs activates IRE1α. (E) BMDCs were pulsed for 4 hr with ovalbumin or human soluble CD4-Fc fusion protein (both at 500 µg/ml), combined with DMSO or G9668 (3 µM) or amiloride (10 µM), and analyzed by IB.

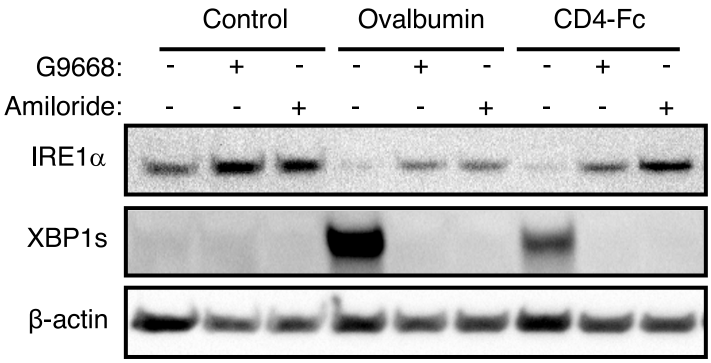

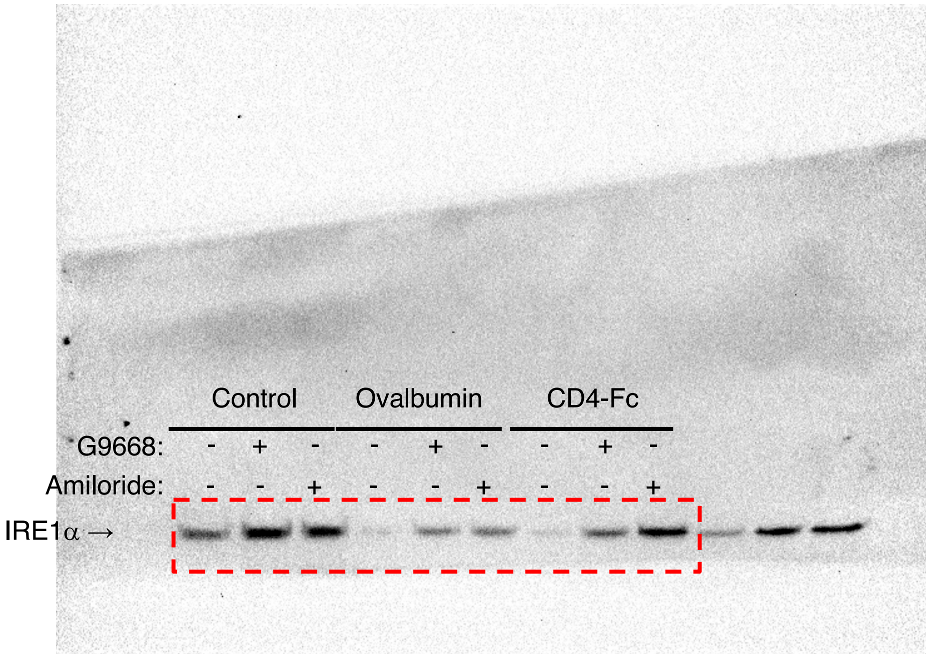

Figure 1. Antigen pulsing of BMDCs activates IRE1α. (E) BMDCs were pulsed for 4 hr with ovalbumin or human soluble CD4-Fc fusion protein (both at 500 µg/ml), combined with DMSO or G9668 (3 µM) or amiloride (10 µM), and analyzed by IB.

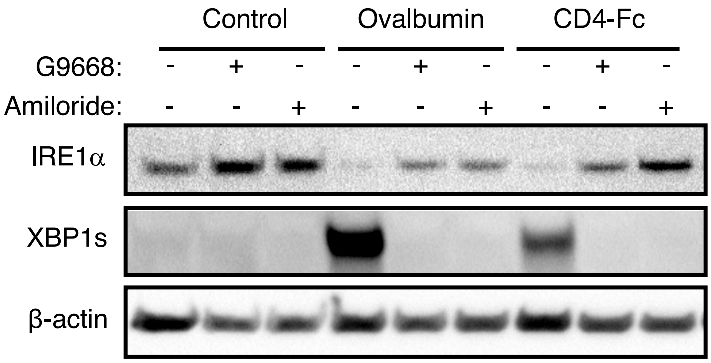

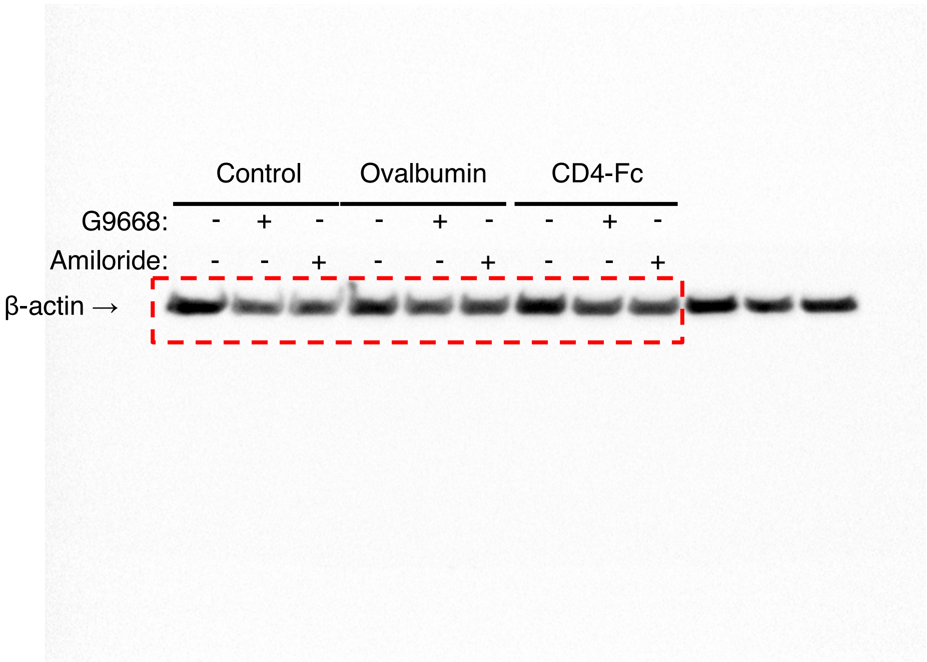

Figure 1. Antigen pulsing of BMDCs activates IRE1α. (E) BMDCs were pulsed for 4 hr with ovalbumin or human soluble CD4-Fc fusion protein (both at 500 µg/ml), combined with DMSO or G9668 (3 µM) or amiloride (10 µM), and analyzed by IB.

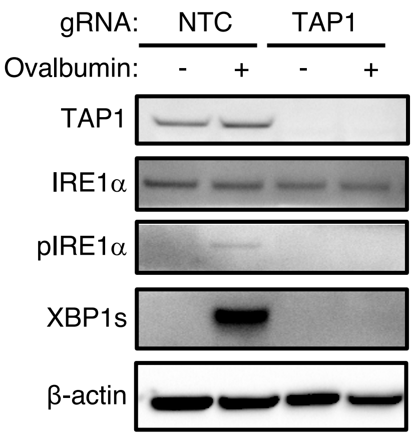

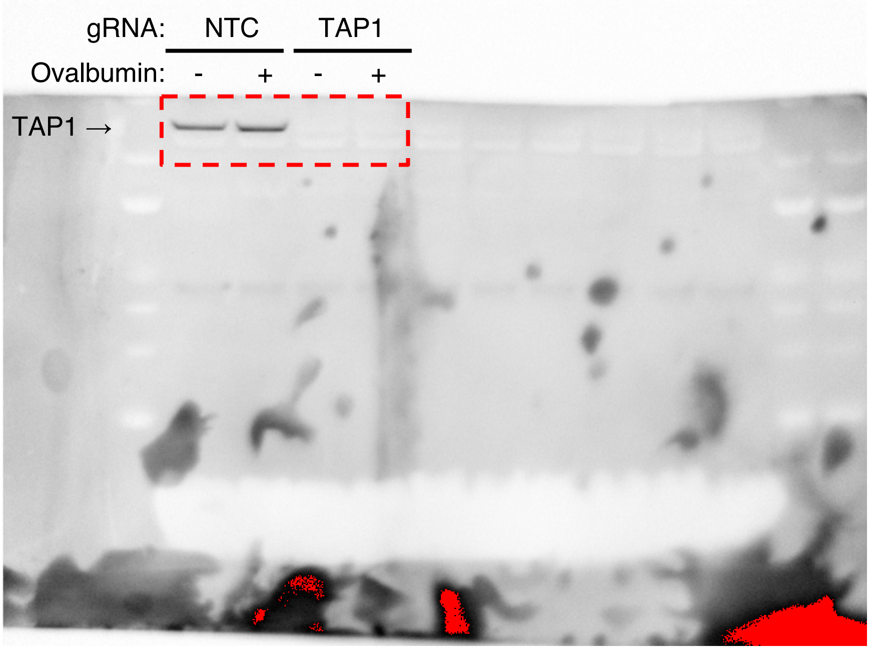

Figure 1. Antigen pulsing of BMDCs activates IRE1α. (F) Upon removal from bone marrow, total bone marrow cells were transfected with non-targeting control (NTC) or TAP1-targeting guide RNAs (gRNA), along with CRISPR/Cas9 delivery constructs. Cells were incubated in BMDC-differentiation media for 9 days, pulsed with ovalbumin (500 µg/ml) for 4 hr, and analyzed by IB. All western blot images are representative of at least two similar experiments.

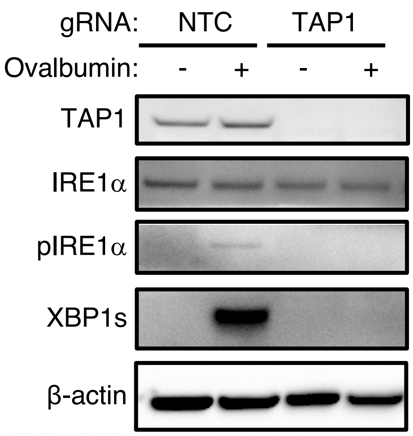

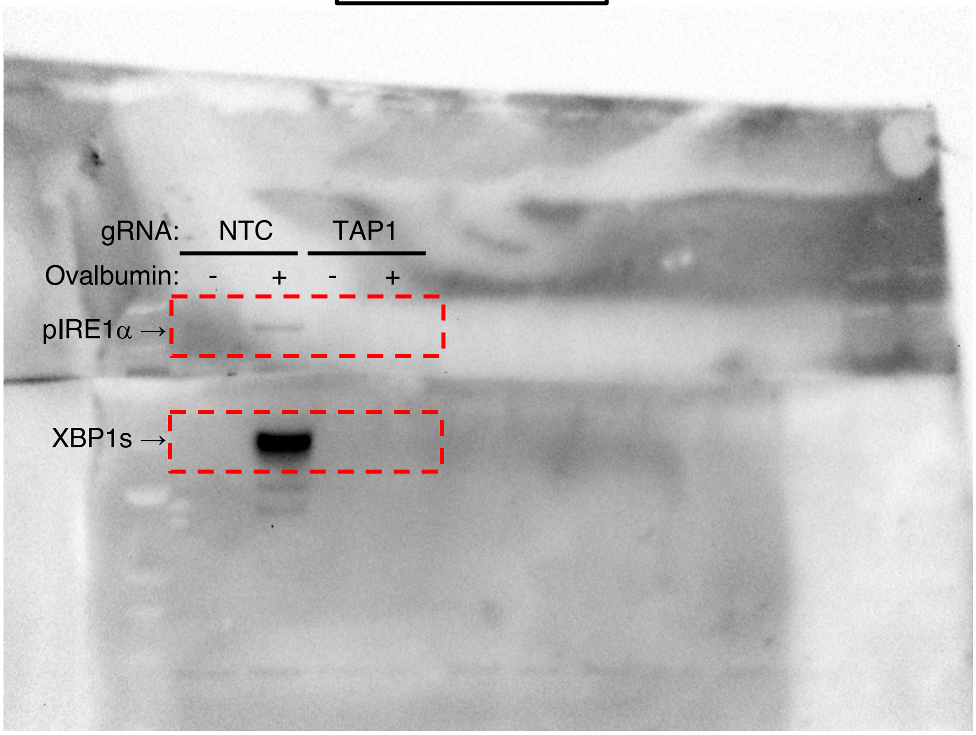

Figure 1. Antigen pulsing of BMDCs activates IRE1α. (F) Upon removal from bone marrow, total bone marrow cells were transfected with non-targeting control (NTC) or TAP1-targeting guide RNAs (gRNA), along with CRISPR/Cas9 delivery constructs. Cells were incubated in BMDC-differentiation media for 9 days, pulsed with ovalbumin (500 µg/ml) for 4 hr, and analyzed by IB. All western blot images are representative of at least two similar experiments.

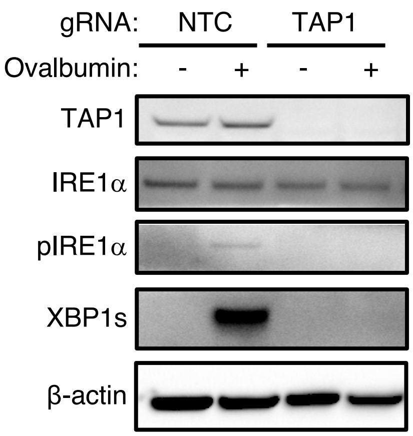

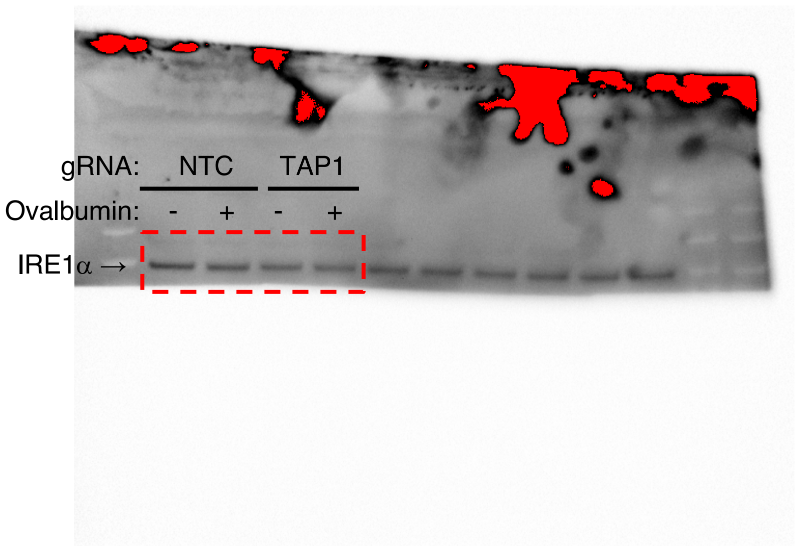

Figure 1. Antigen pulsing of BMDCs activates IRE1α. (F) Upon removal from bone marrow, total bone marrow cells were transfected with non-targeting control (NTC) or TAP1-targeting guide RNAs (gRNA), along with CRISPR/Cas9 delivery constructs. Cells were incubated in BMDC-differentiation media for 9 days, pulsed with ovalbumin (500 µg/ml) for 4 hr, and analyzed by IB. All western blot images are representative of at least two similar experiments.

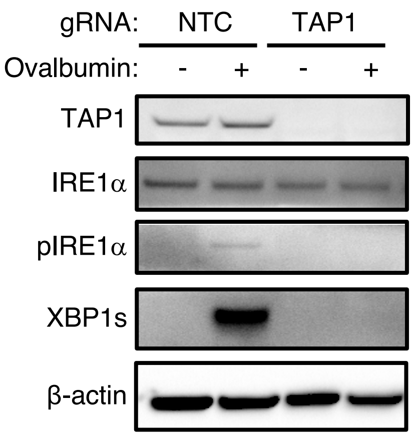

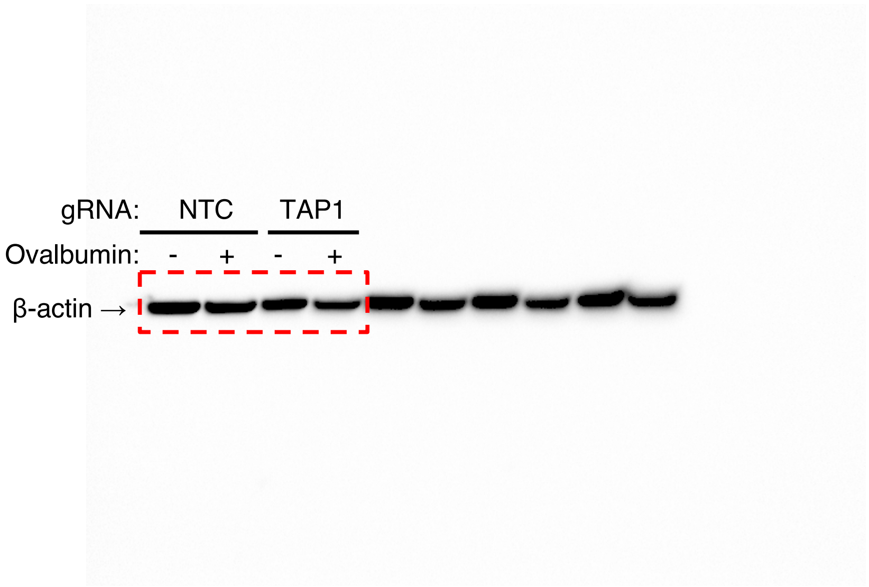

Figure 1. Antigen pulsing of BMDCs activates IRE1α. (F) Upon removal from bone marrow, total bone marrow cells were transfected with non-targeting control (NTC) or TAP1-targeting guide RNAs (gRNA), along with CRISPR/Cas9 delivery constructs. Cells were incubated in BMDC-differentiation media for 9 days, pulsed with ovalbumin (500 µg/ml) for 4 hr, and analyzed by IB. All western blot images are representative of at least two similar experiments.
