# Supplementary material for: Antigen-derived peptides engage the ER stress sensor IRE1α to curb dendritic cell cross-presentation
Source: J Cell Biol. 2022 Apr 21;221(6):e202111068. doi: 10.1083/jcb.202111068 (PMC9036094; doi:10.1083/jcb.202111068)
Supplement: SourceData F2 — contains original blots for Fig. 2. [file JCB_202111068_SourceDataF2.pdf]

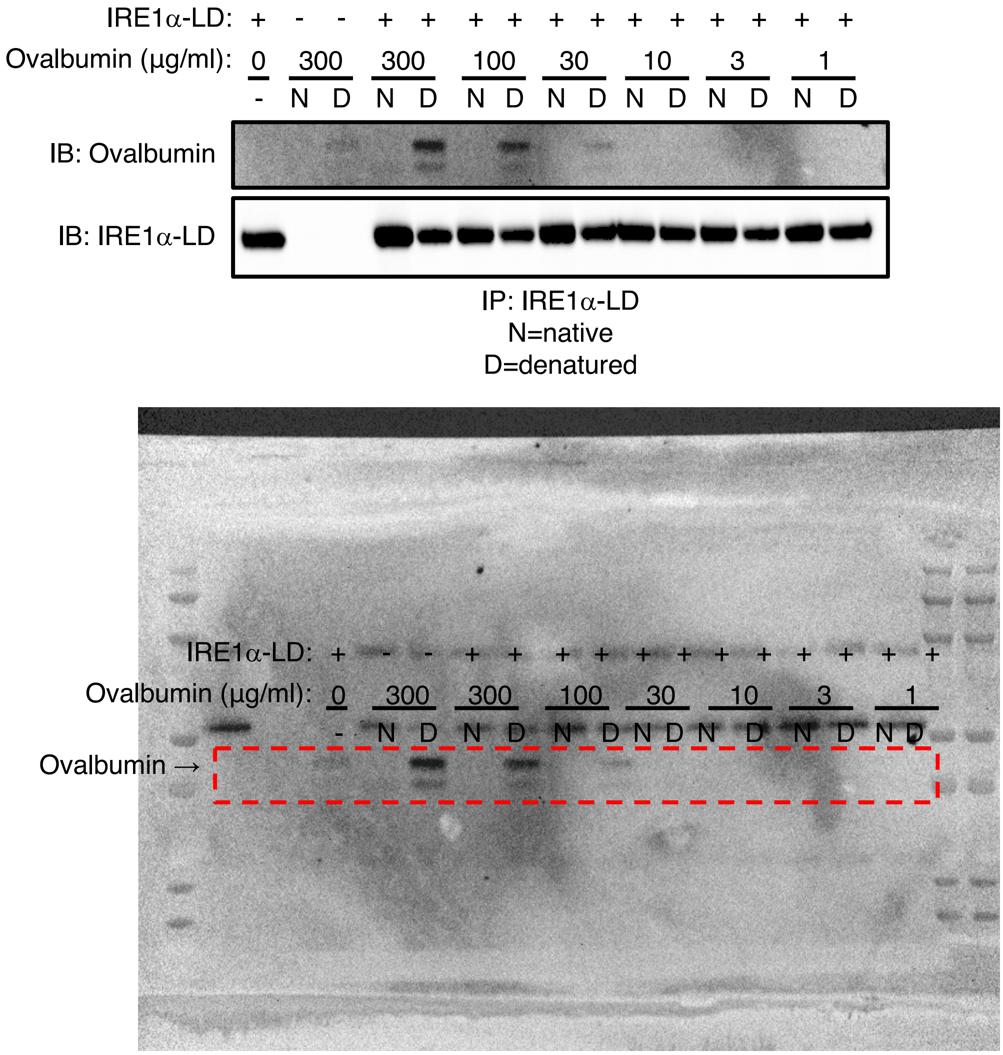

Figure 2. Antigen-derived peptides can directly engage IRE1α. (B) Native or heat-denatured ovalbumin at indicated concentrations was incubated with IRE1α LD-Fc (10 µg/ml), immunoprecipitated via monoclonal anti-IRE1α LD antibody, and analyzed by IB.

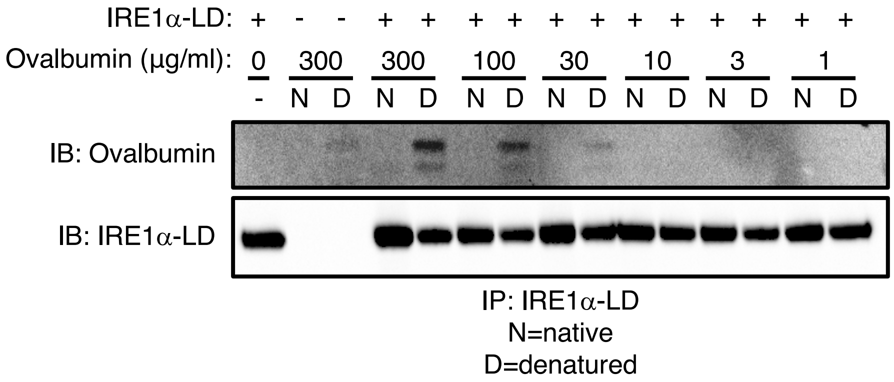

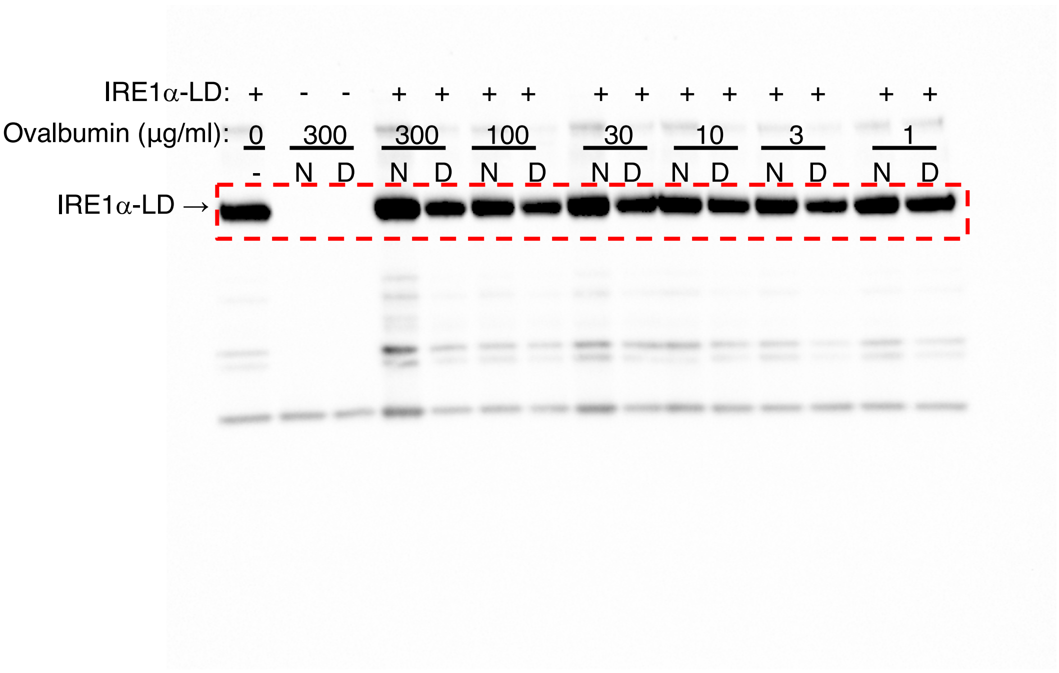

Figure 2. Antigen-derived peptides can directly engage IRE1α. (B) Native or heat-denatured ovalbumin at indicated concentrations was incubated with IRE1α LD-Fc (10 µg/ml), immunoprecipitated via monoclonal anti-IRE1α LD antibody, and analyzed by IB.

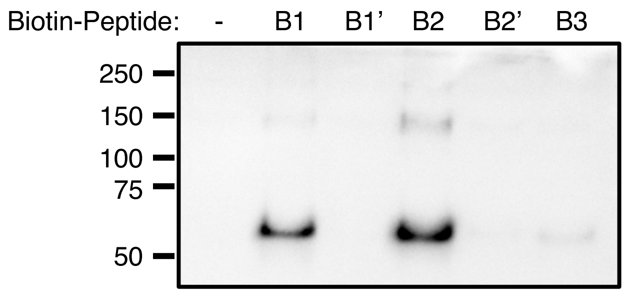

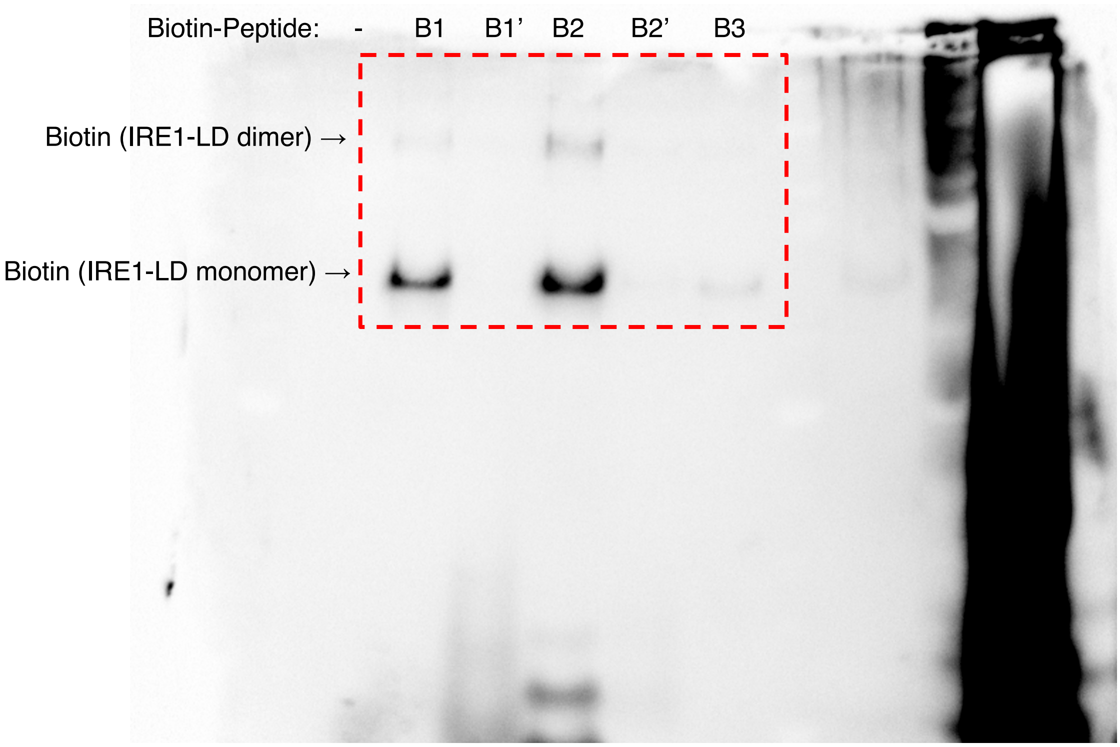

Figure 2. Antigen-derived peptides can directly engage IRE1α. (D) Biotin-tagged ovalbumin-based peptides (100 μM) were incubated with FLAG-tagged IRE1α LD (50 μM), cross-linked with disuccinimidyl suberate (DSS) and analyzed by IB. B1, B2, B3 are WT peptides; B1’, B2’ are mutant peptides in which all hydrophobic residues were replaced by aspartic acid.

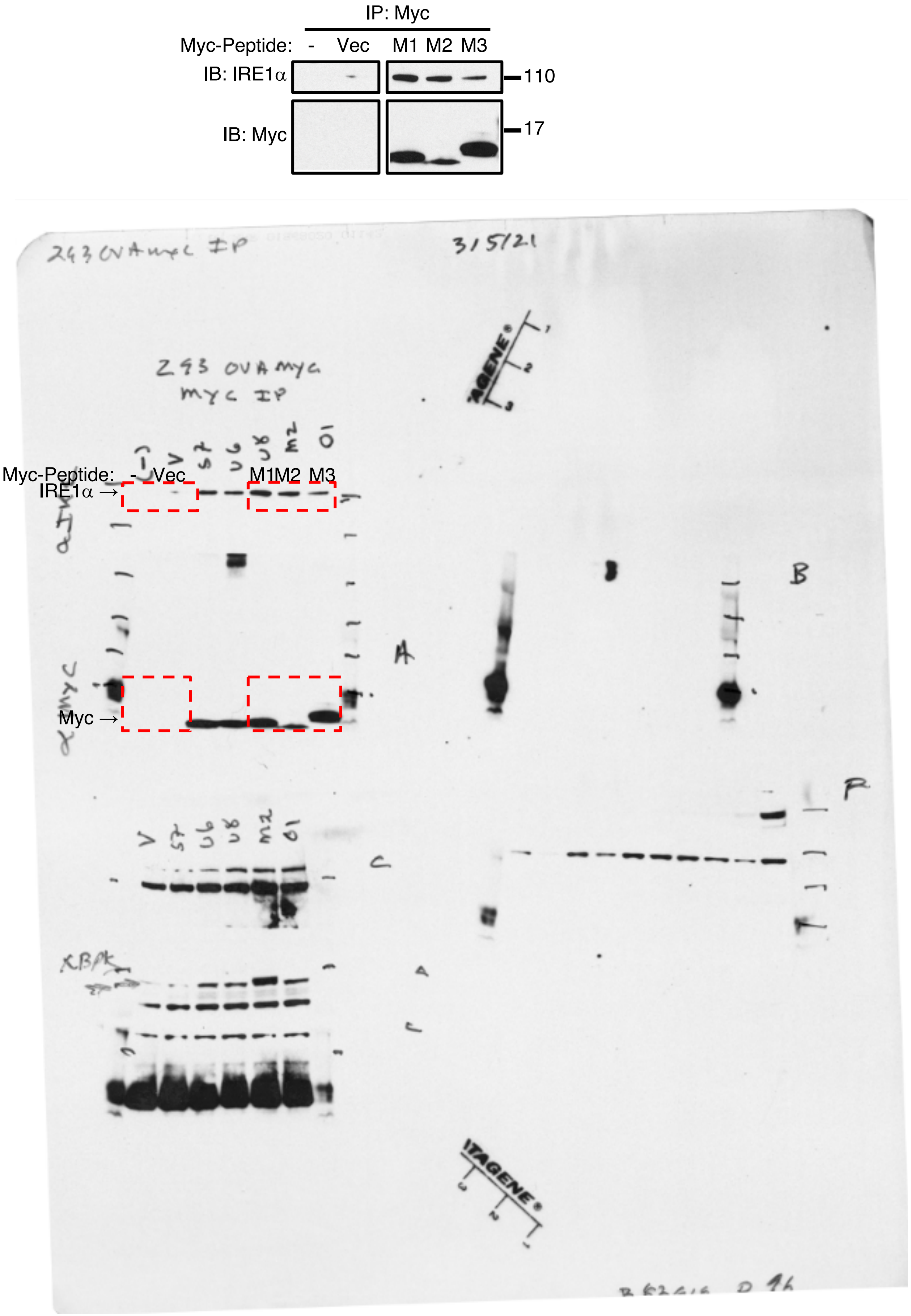

Figure 2. Antigen-derived peptides can directly engage IRE1α. (E) HEK293 cells were transfected with cDNA constructs encoding Myc-tagged peptides (M1, M2, M3) derived from corresponding ovalbumin regions (B1, B2, B3) and containing an ER-directed signal sequence for 48 hr, followed by immunoprecipitation with anti-Myc antibody and IB for IRE1α or Myc. Bar graph indicates signal ratio for IRE1α over Myc.
