# Supplementary material for: Antigen-derived peptides engage the ER stress sensor IRE1α to curb dendritic cell cross-presentation
Source: J Cell Biol. 2022 Apr 21;221(6):e202111068. doi: 10.1083/jcb.202111068 (PMC9036094; doi:10.1083/jcb.202111068)
Supplement: SourceData F4 — contains original blots for Fig. 4. [file JCB_202111068_SourceDataF4.pdf]

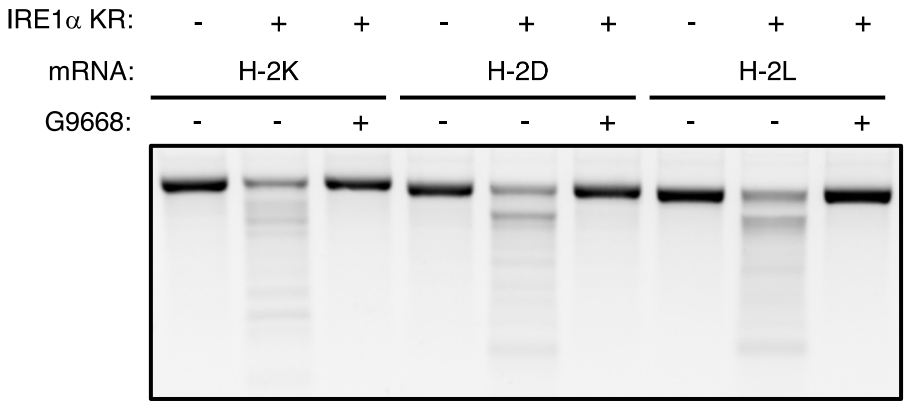

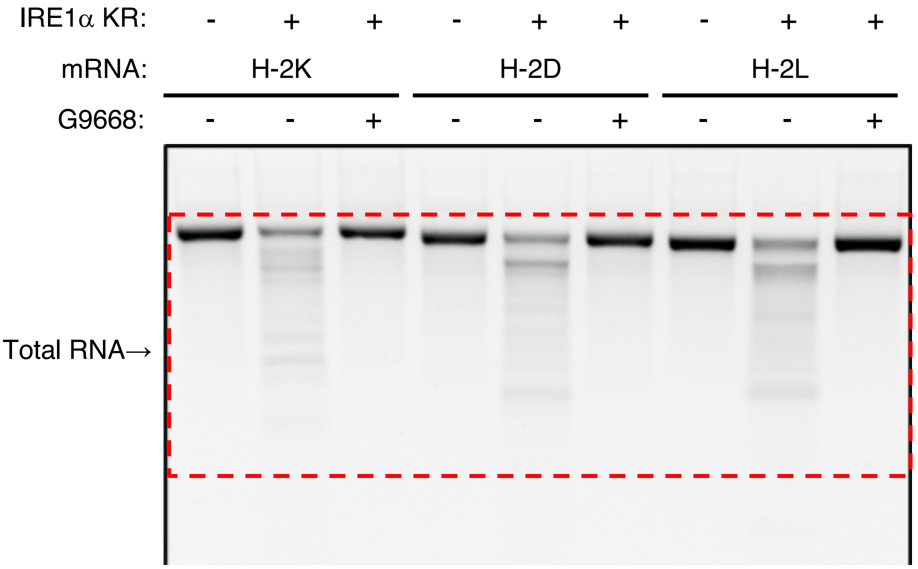

Figure 4. IRE1α activation depletes MHC-I heavy-chain mRNAs via RIDD. (B) Purified recombinant IRE1α kinase-endoribonucleae (KR) protein was incubated with RNA transcripts of H-2K, H-2D and H-2L, in absence or presence of G9668 (10 μM), followed by agarose gel electrophoresis to determine transcript integrity.
