# Supplementary material for: Antigen-derived peptides engage the ER stress sensor IRE1α to curb dendritic cell cross-presentation
Source: J Cell Biol. 2022 Apr 21;221(6):e202111068. doi: 10.1083/jcb.202111068 (PMC9036094; doi:10.1083/jcb.202111068)
Supplement: SourceData FS1 — contains original blots for Fig. S1. [file JCB_202111068_SourceDataFS1.pdf]

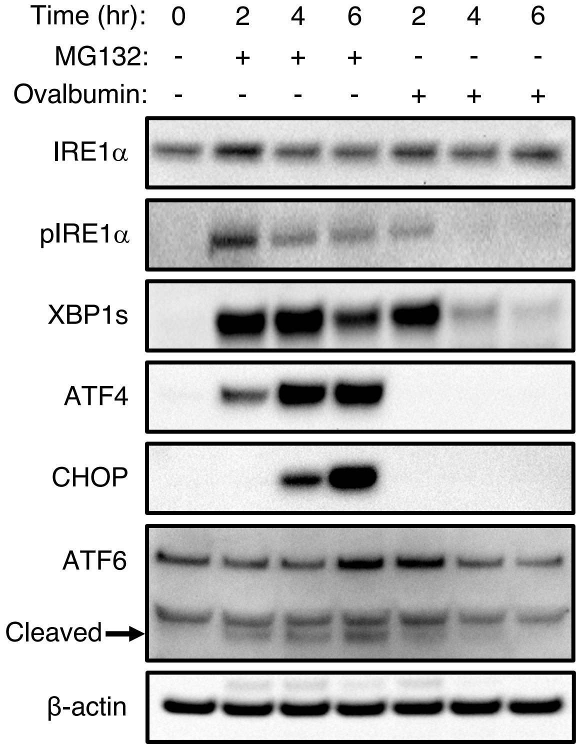

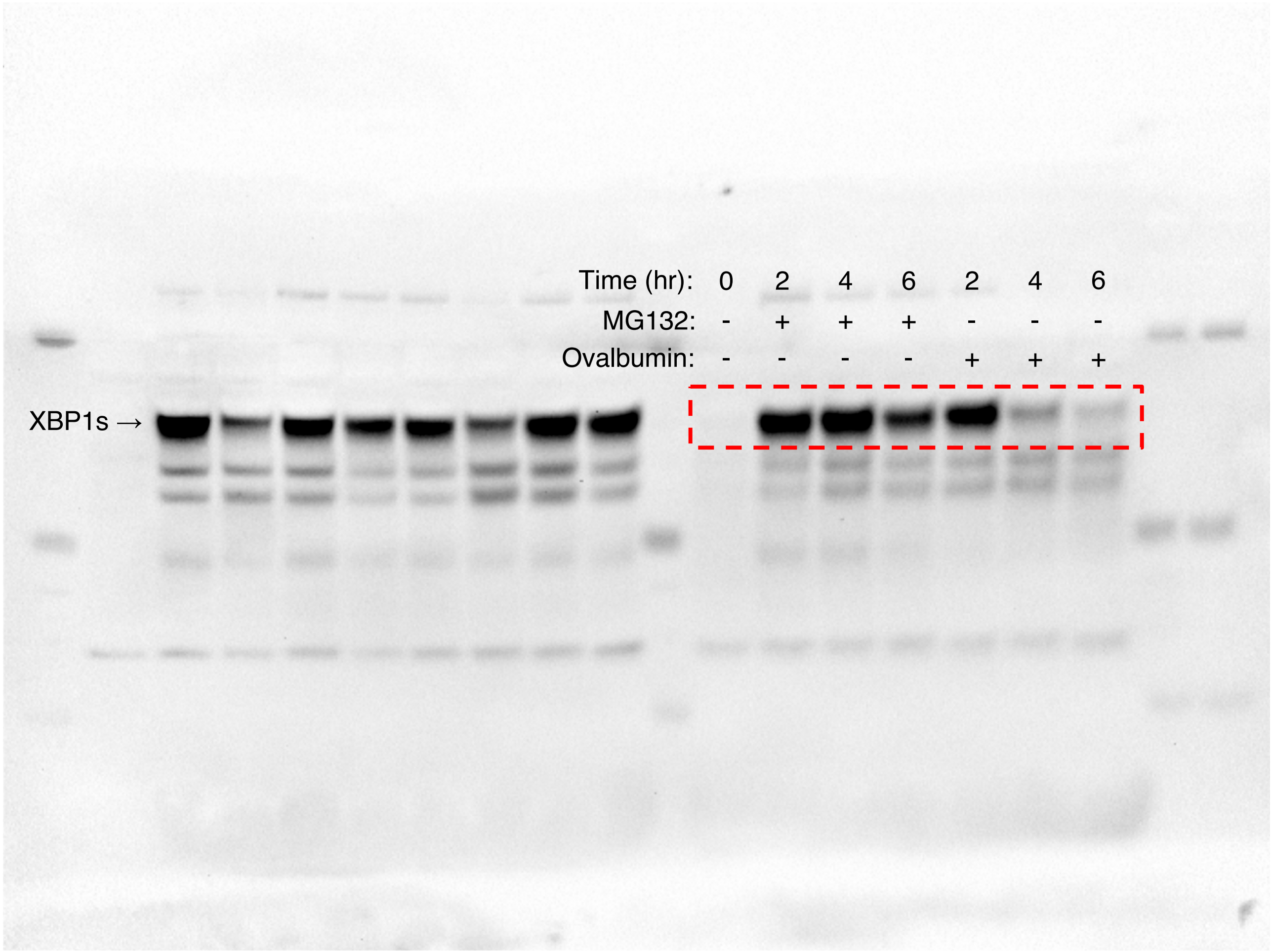

Figure S1. Antigen pulsing of BMDCs activates IRE1α. (A) BMDCs were treated with MG132 (5 μM) or ovalbumin (500 μg/ml) for indicated time periods and analyzed by IB.

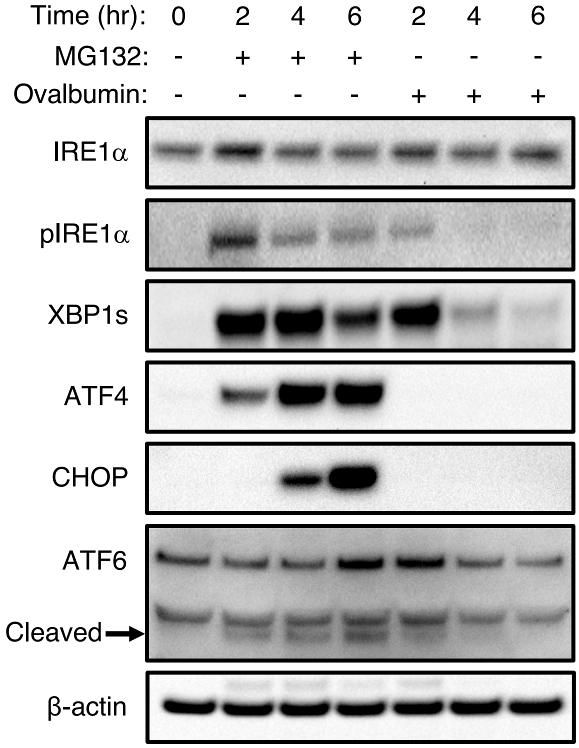

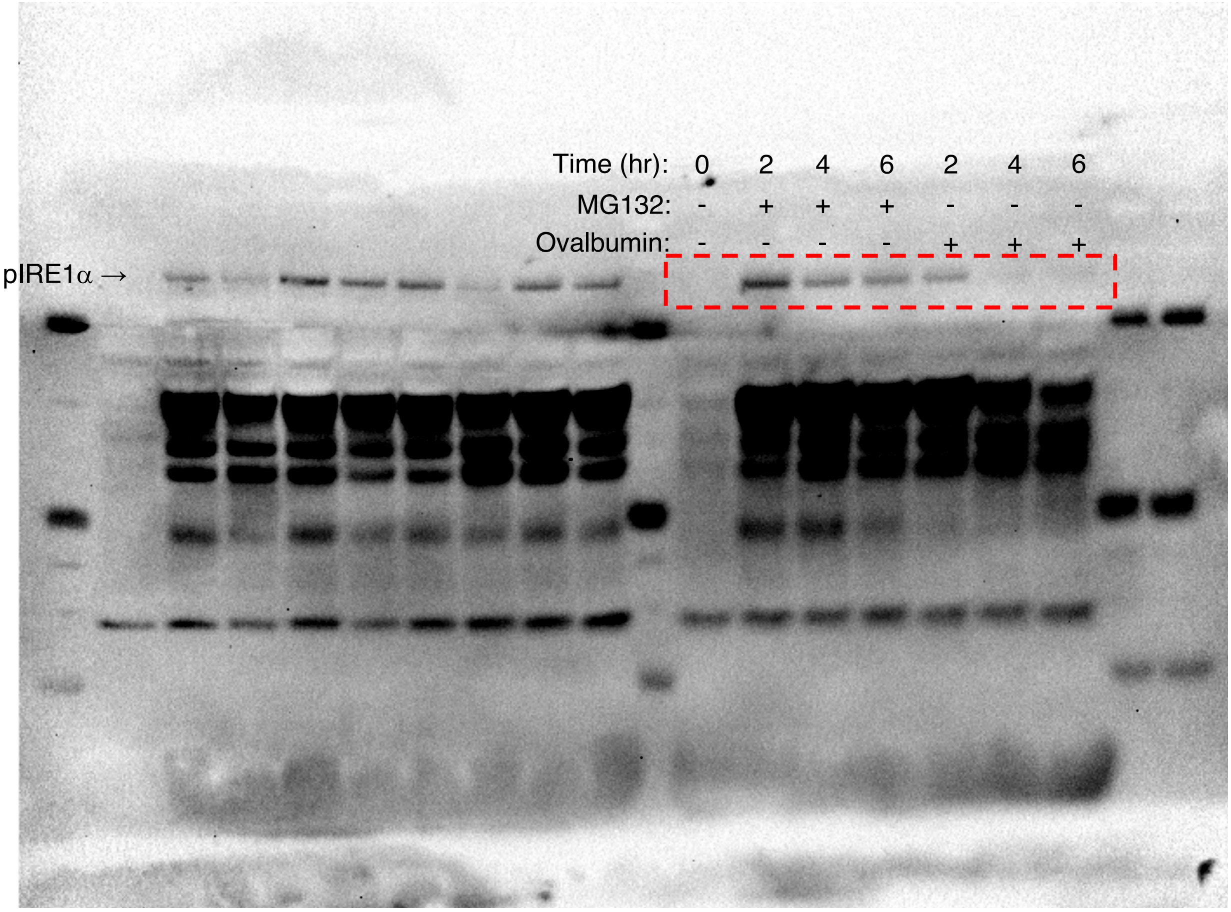

Figure S1. Antigen pulsing of BMDCs activates IRE1α. (A) BMDCs were treated with MG132 (5 μM) or ovalbumin (500 μg/ml) for indicated time periods and analyzed by IB.

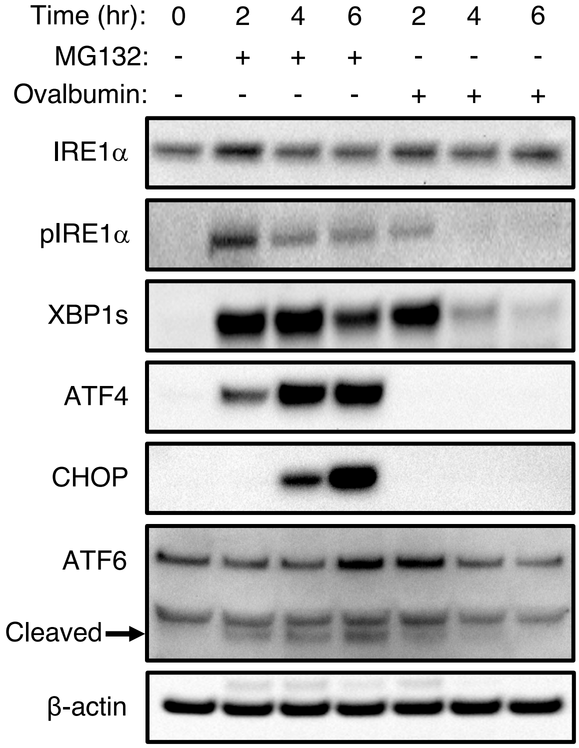

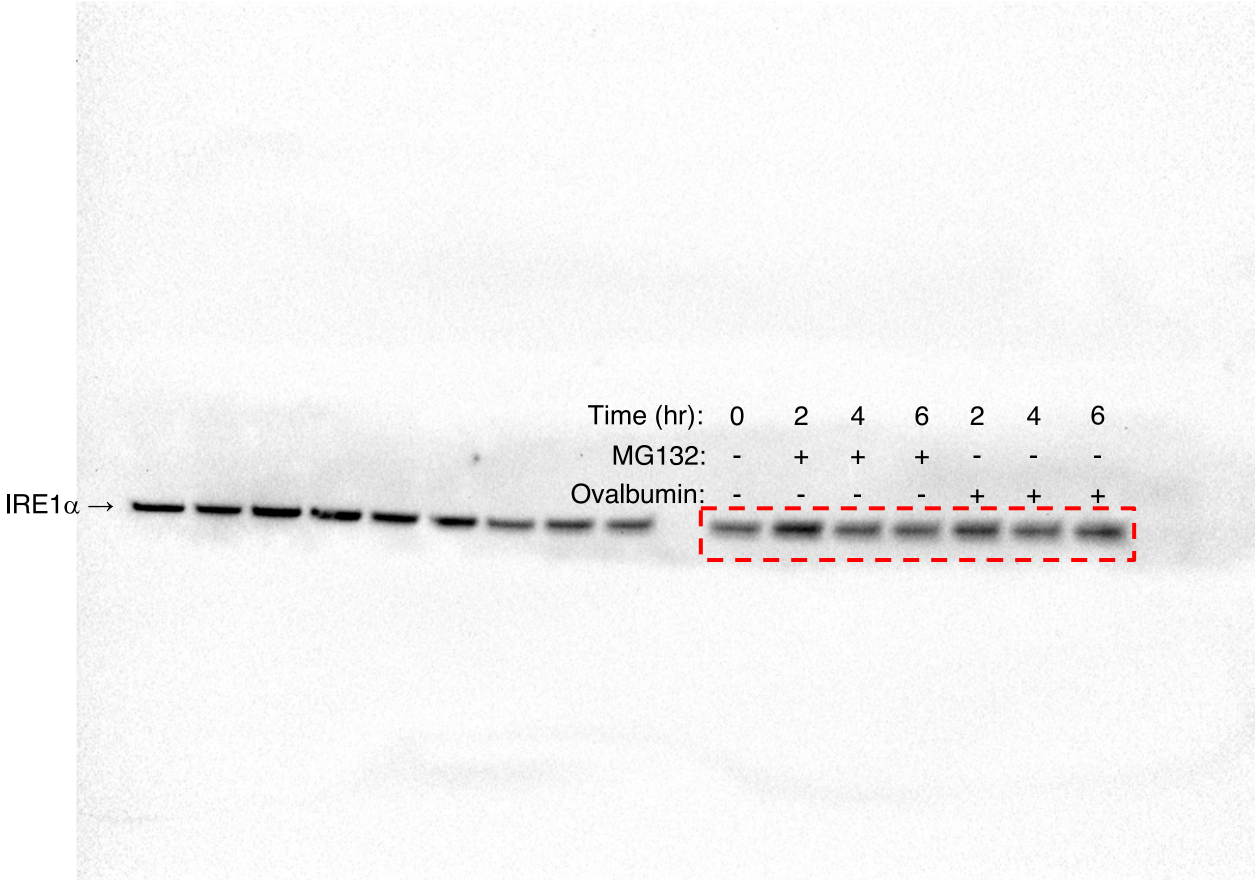

Figure S1. Antigen pulsing of BMDCs activates IRE1α. (A) BMDCs were treated with MG132 (5 µM) or ovalbumin (500 µg/ml) for indicated time periods and analyzed by IB.

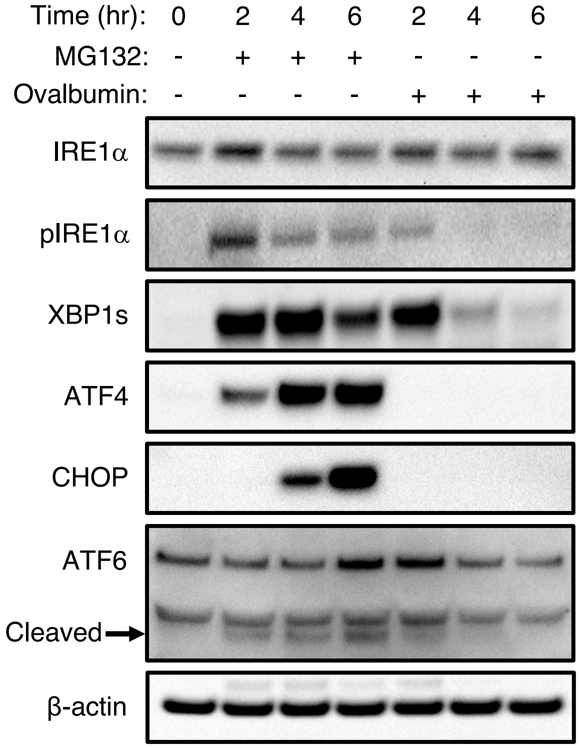

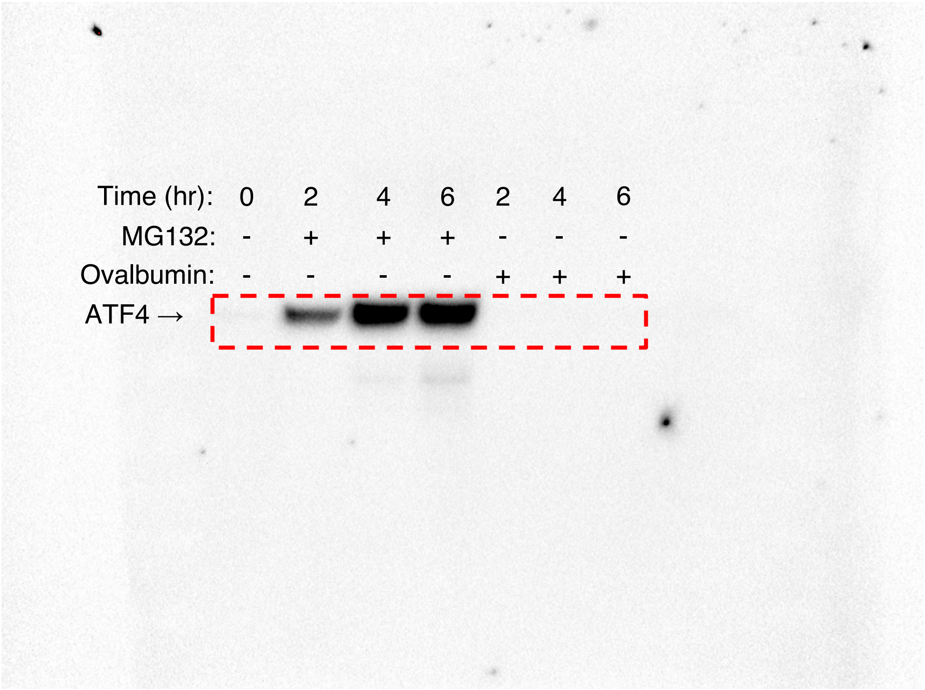

Figure S1. Antigen pulsing of BMDCs activates IRE1α. (A) BMDCs were treated with MG132 (5 μM) or ovalbumin (500 μg/ml) for indicated time periods and analyzed by IB.

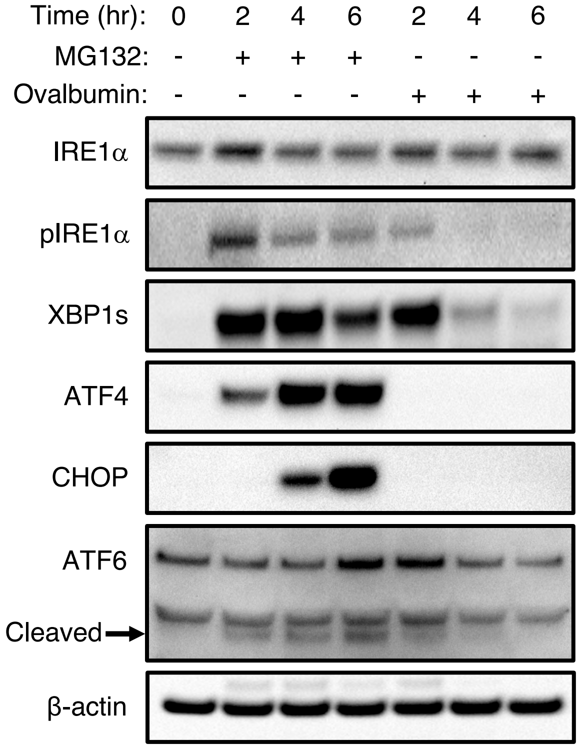

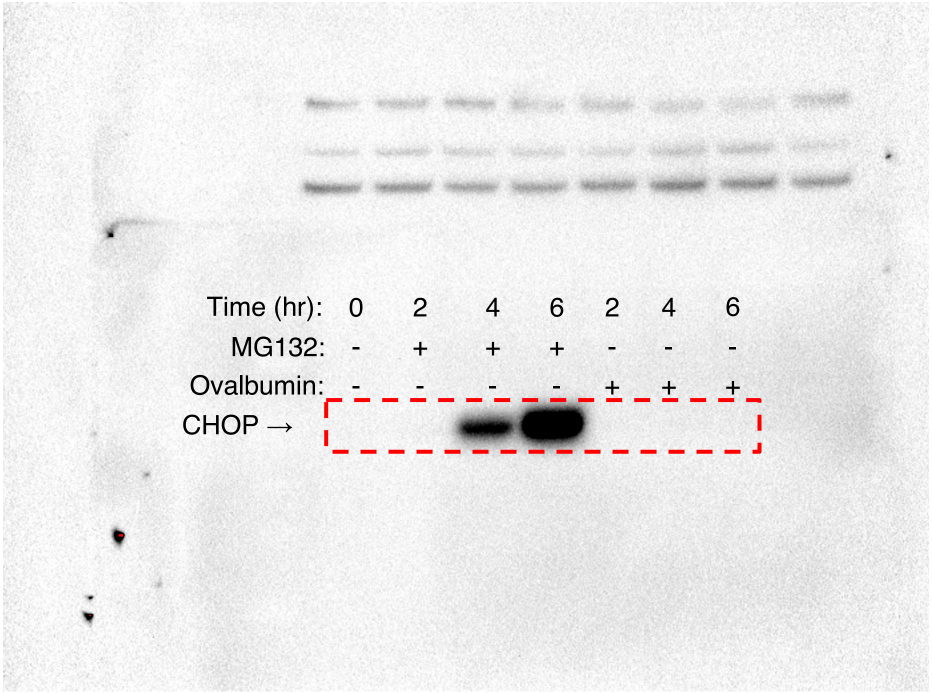

Figure S1. Antigen pulsing of BMDCs activates IRE1α. (A) BMDCs were treated with MG132 (5 µM) or ovalbumin (500 µg/ml) for indicated time periods and analyzed by IB.

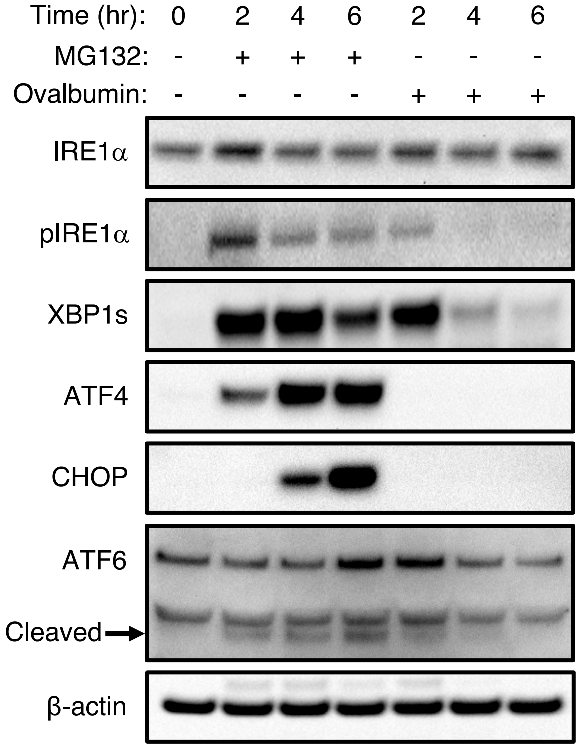

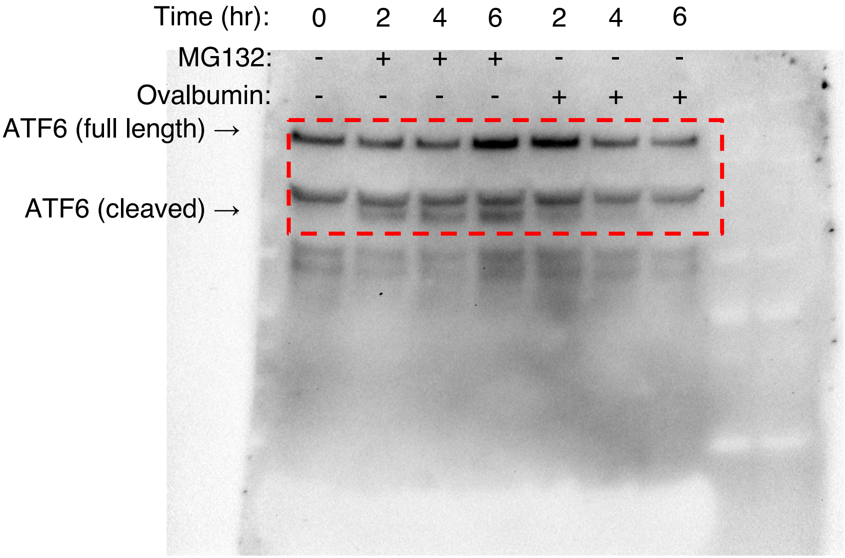

Figure S1. Antigen pulsing of BMDCs activates IRE1α. (A) BMDCs were treated with MG132 (5 µM) or ovalbumin (500 µg/ml) for indicated time periods and analyzed by IB.

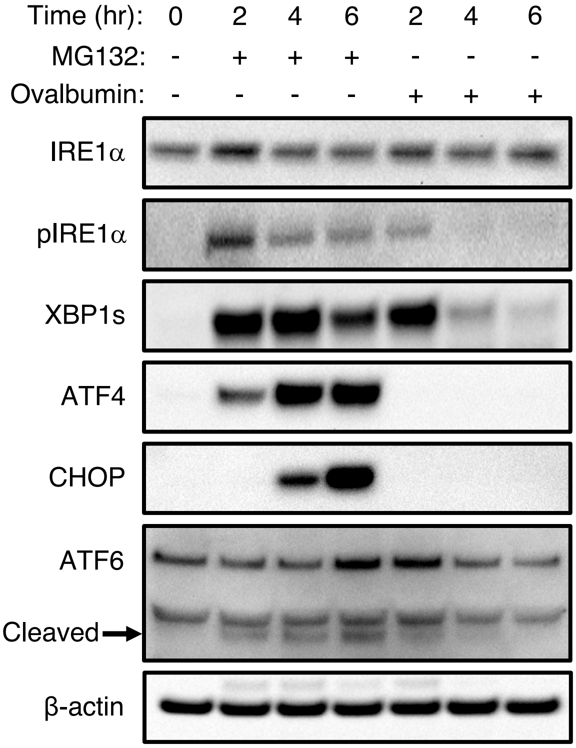

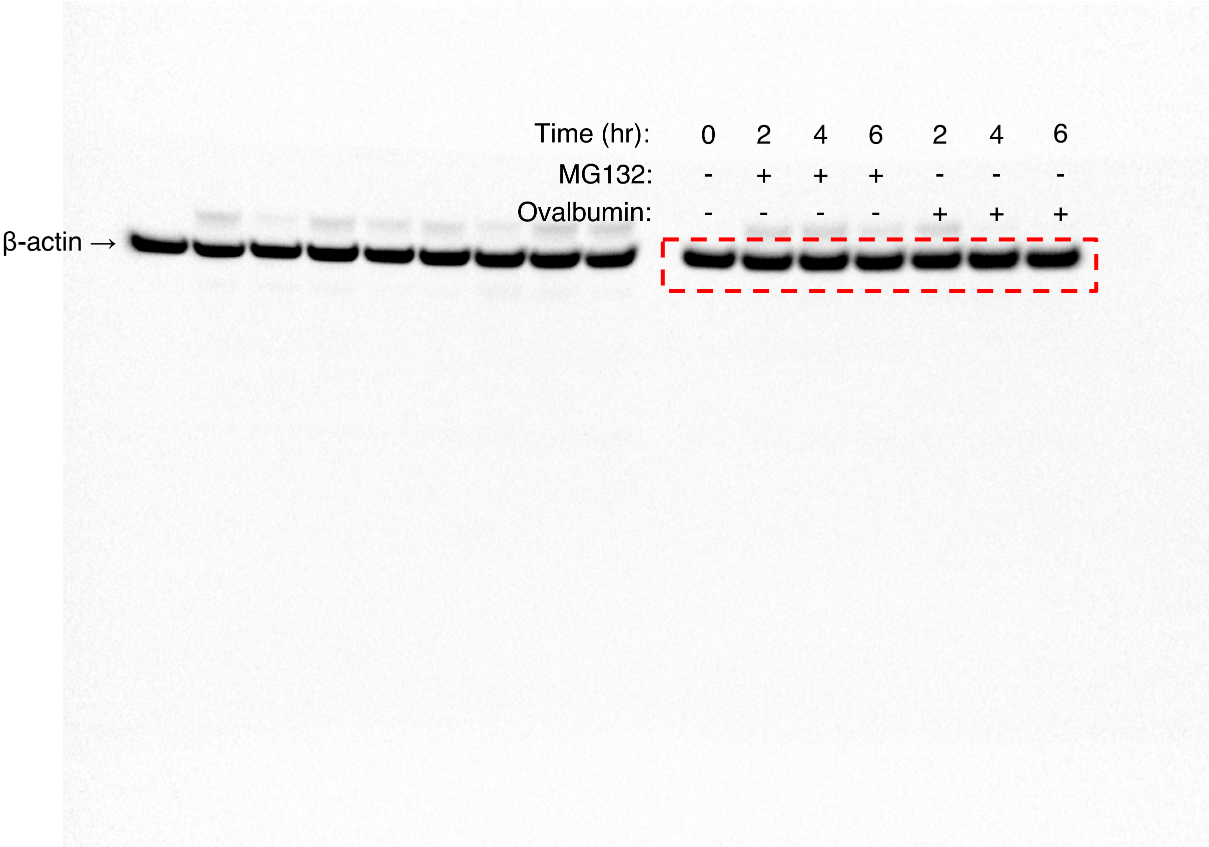

Figure S1. Antigen pulsing of BMDCs activates IRE1α. (A) BMDCs were treated with MG132 (5 µM) or ovalbumin (500 µg/ml) for indicated time periods and analyzed by IB.

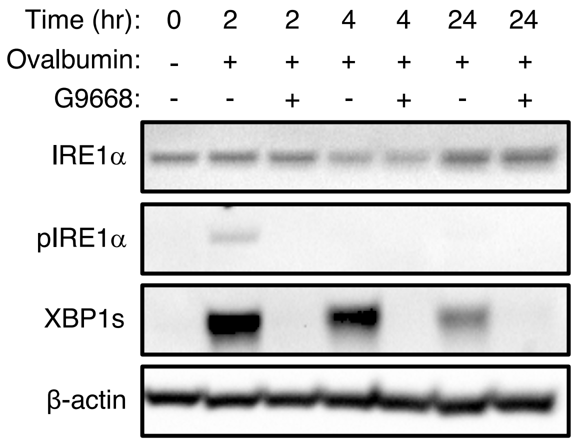

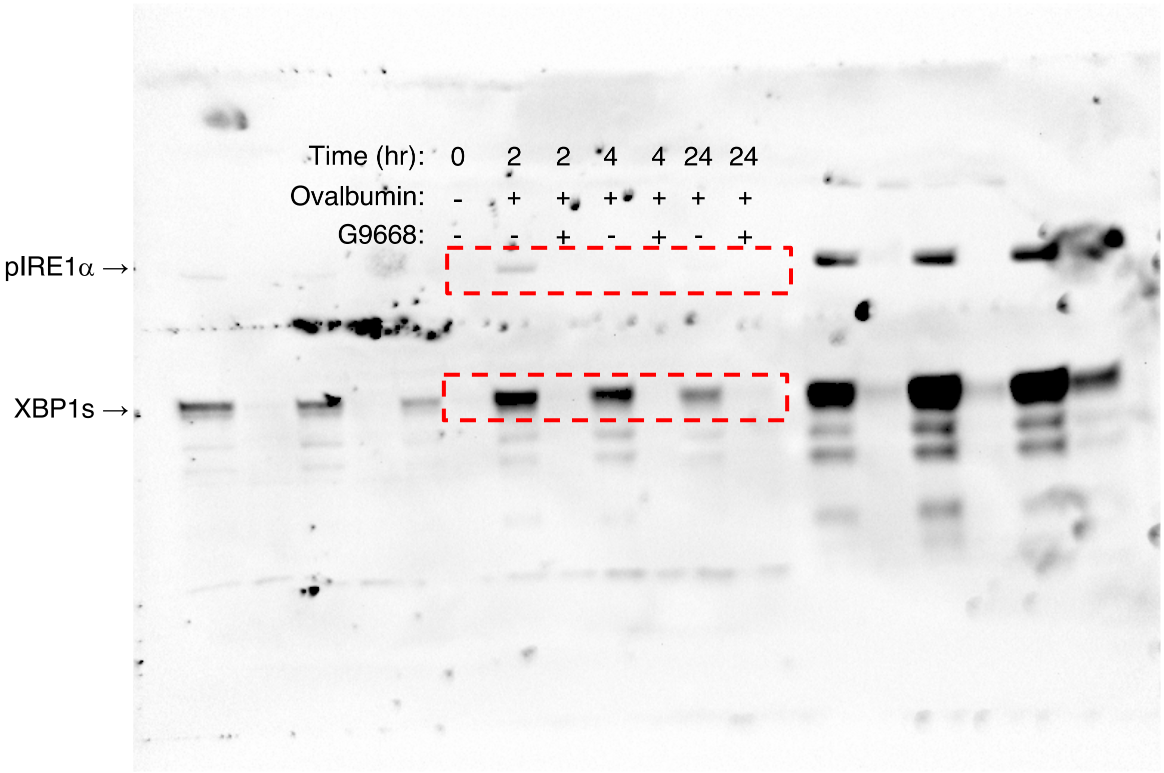

Figure S1. Antigen pulsing of BMDCs activates IRE1α. **(B)** BMDCs were pulsed with ovalbumin (500 µg/ml) and G9668 (3 µM) for indicated time periods and analyzed by IB.

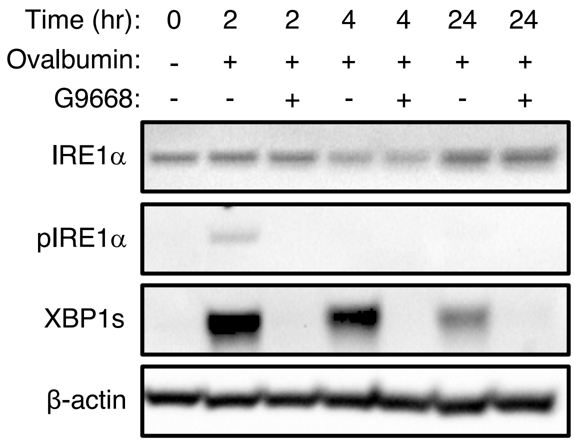

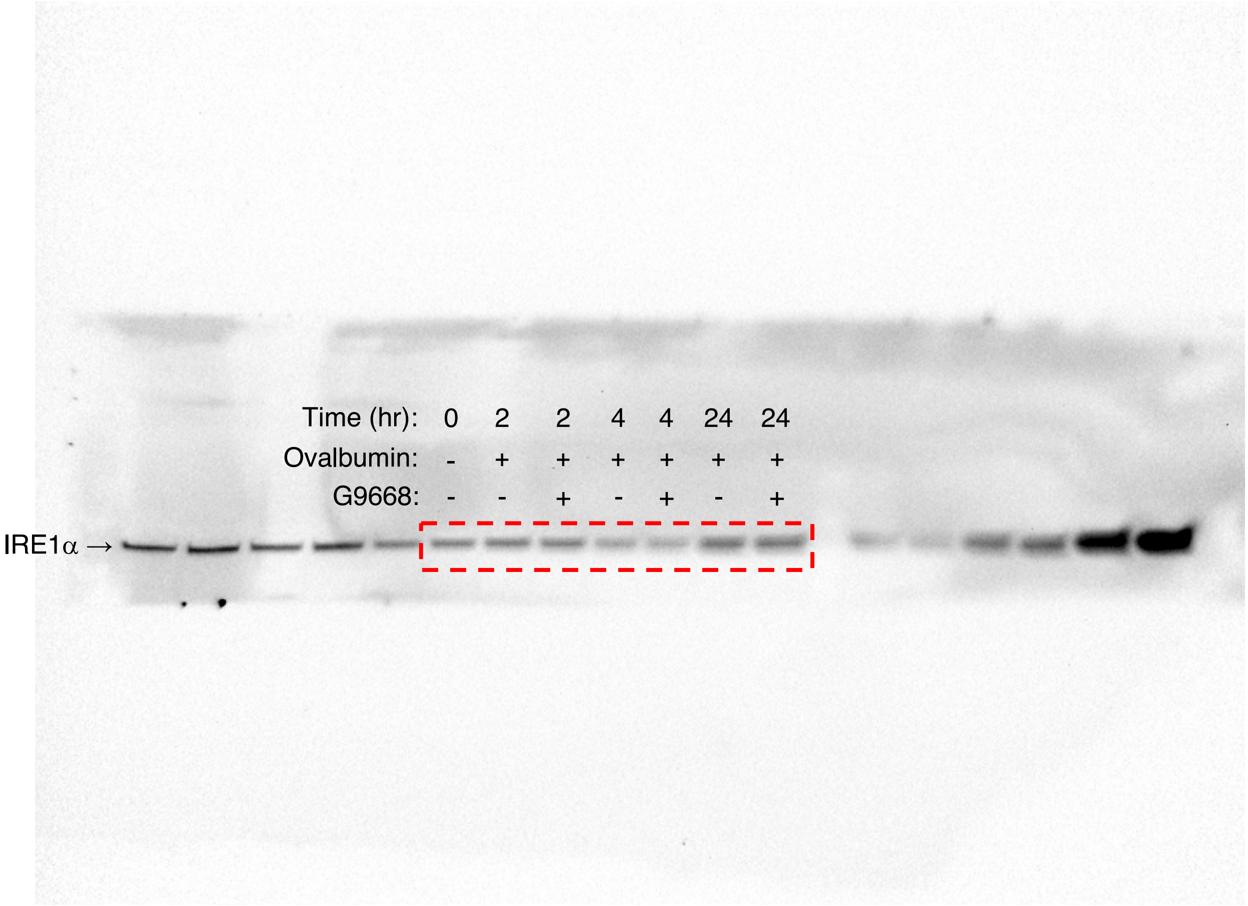

Figure S1. Antigen pulsing of BMDCs activates IRE1α. **(B)** BMDCs were pulsed with ovalbumin (500 µg/ml) and G9668 (3 µM) for indicated time periods and analyzed by IB.

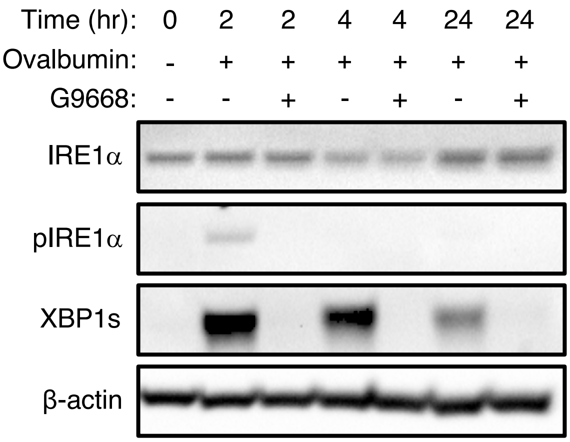

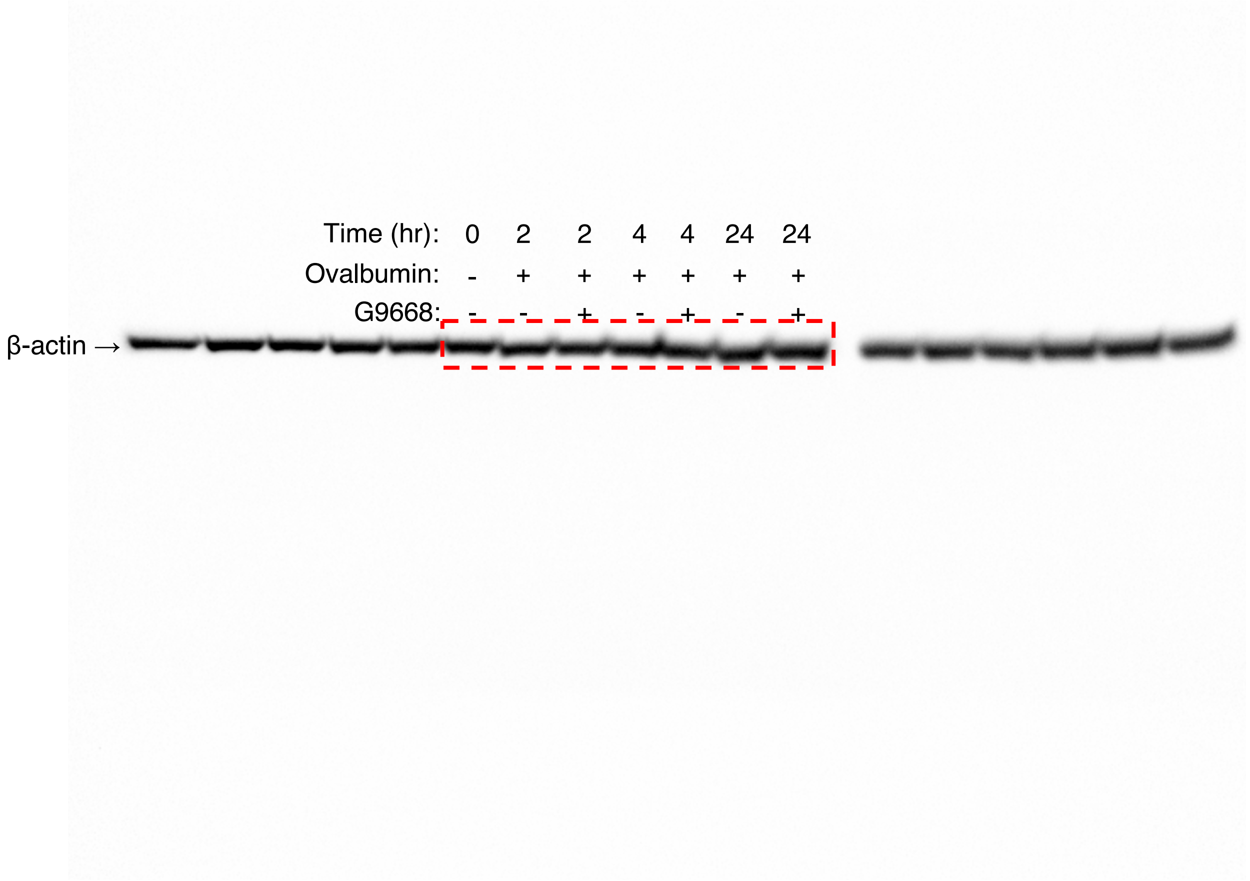

Figure S1. Antigen pulsing of BMDCs activates IRE1α. **(B)** BMDCs were pulsed with ovalbumin (500 µg/ml) and G9668 (3 µM) for indicated time periods and analyzed by IB.

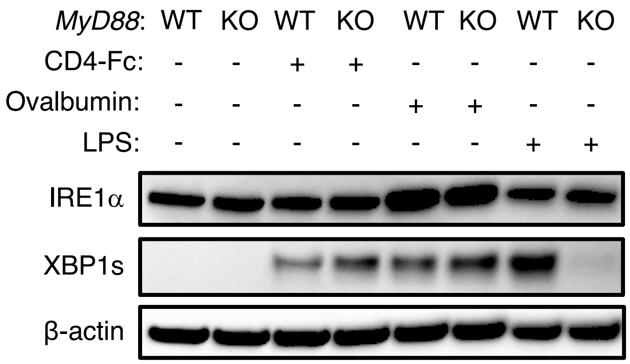

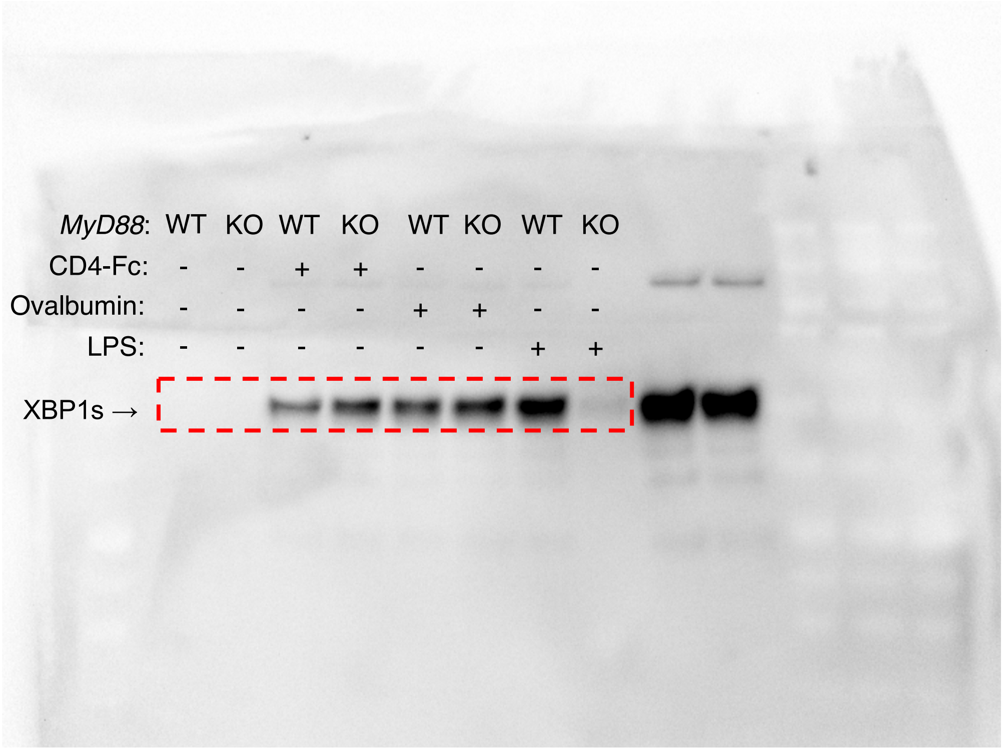

Figure S1. Antigen pulsing of BMDCs activates IRE1α. (F) WT or MyD88 KO Flt3L-derived BMDCs were pulsed with ovalbumin (500 μg/ml) or soluble CD4-Fc protein (500 μg/ml), or treated with LPS (10 μg/ml) for 4 hr and analyzed by IB.

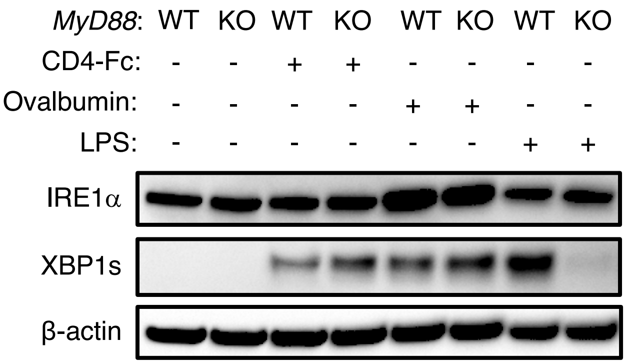

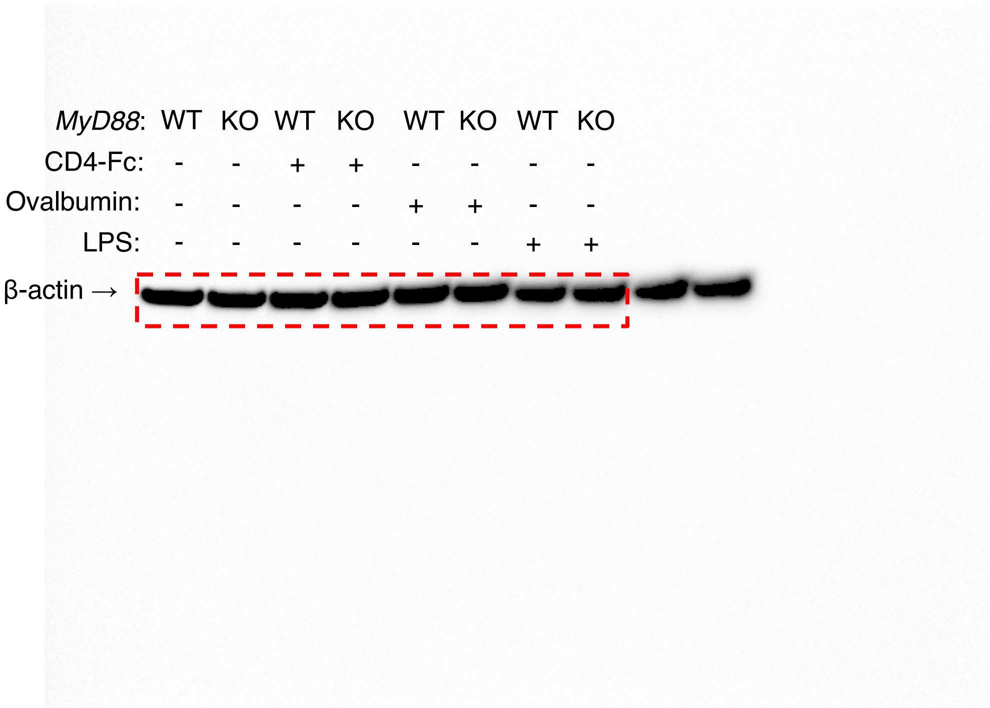

Figure S1. Antigen pulsing of BMDCs activates IRE1α. (F) WT or MyD88 KO Flt3L-derived BMDCs were pulsed with ovalbumin (500 µg/ml) or soluble CD4-Fc protein (500 µg/ml), or treated with LPS (10 µg/ml) for 4 hr and analyzed by IB.

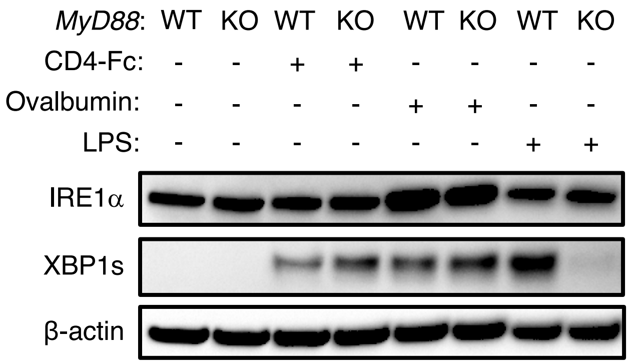

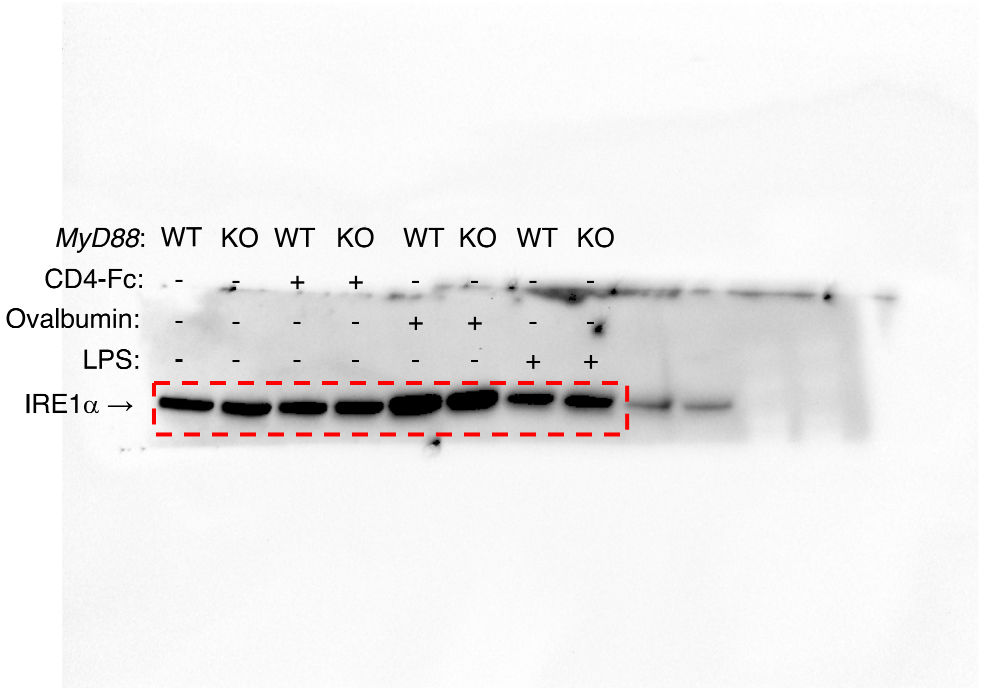

Figure S1. Antigen pulsing of BMDCs activates IRE1α. (F) WT or MyD88 KO Flt3L-derived BMDCs were pulsed with ovalbumin (500 µg/ml) or soluble CD4-Fc protein (500 µg/ml), or treated with LPS (10 µg/ml) for 4 hr and analyzed by IB.

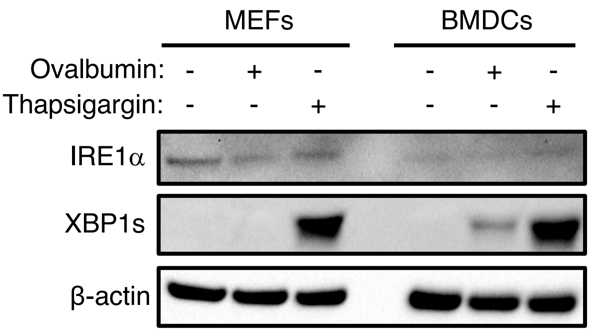

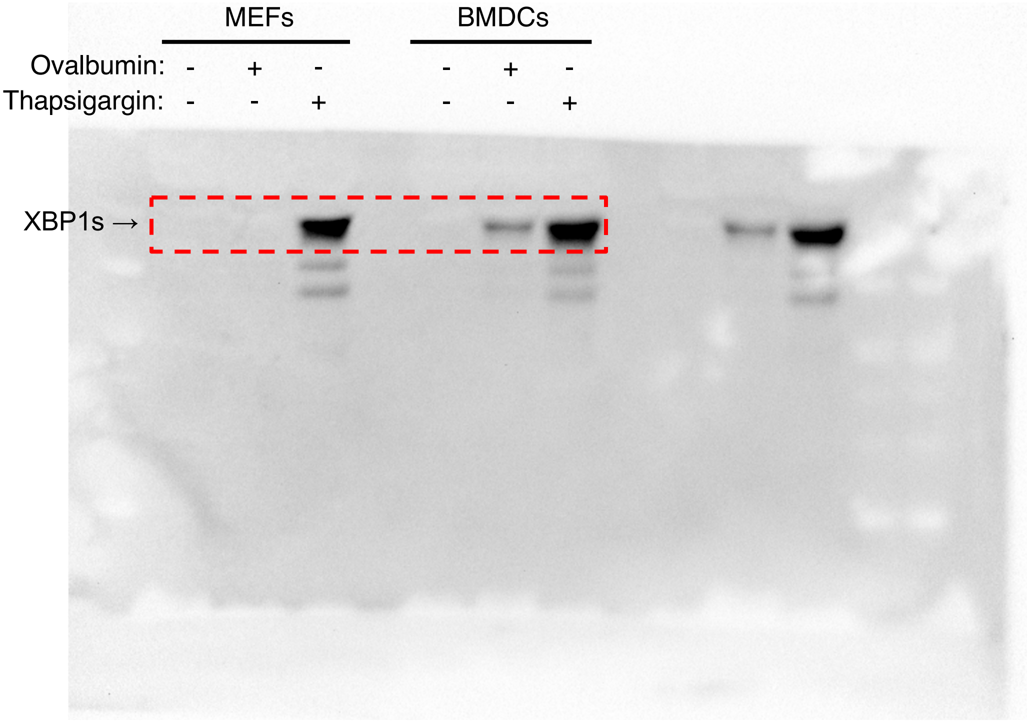

Figure S1. Antigen pulsing of BMDCs activates IRE1α. (G) MEFs and BMDCs were pulsed with ovalbumin or treated with Tg (100 nM) for 4 hr and analyzed by IB.

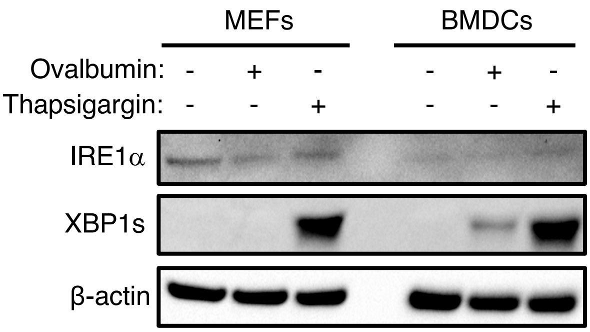

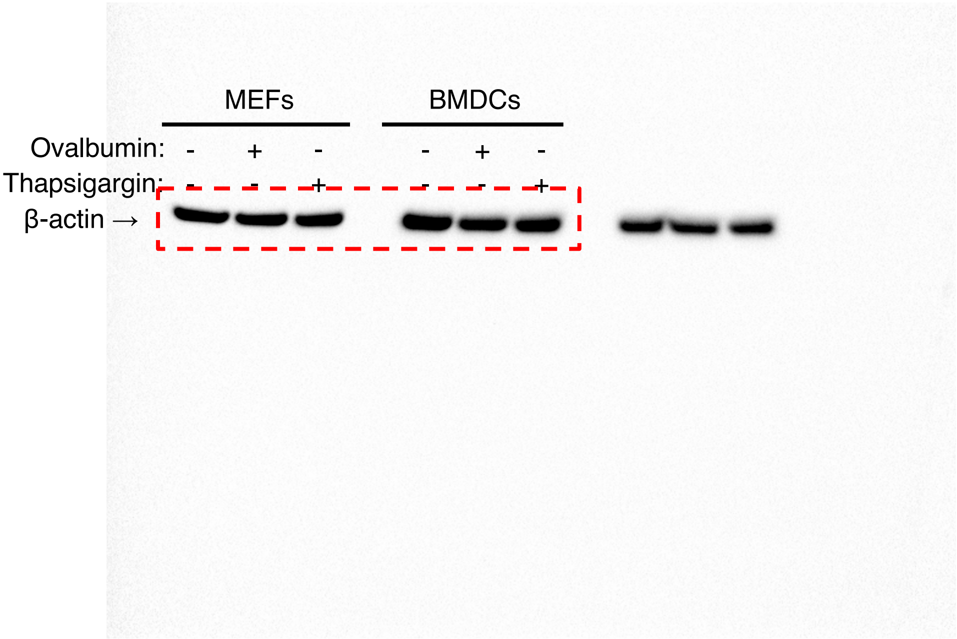

Figure S1. Antigen pulsing of BMDCs activates IRE1α. (G) MEFs and BMDCs were pulsed with ovalbumin or treated with Tg (100 nM) for 4 hr and analyzed by IB.

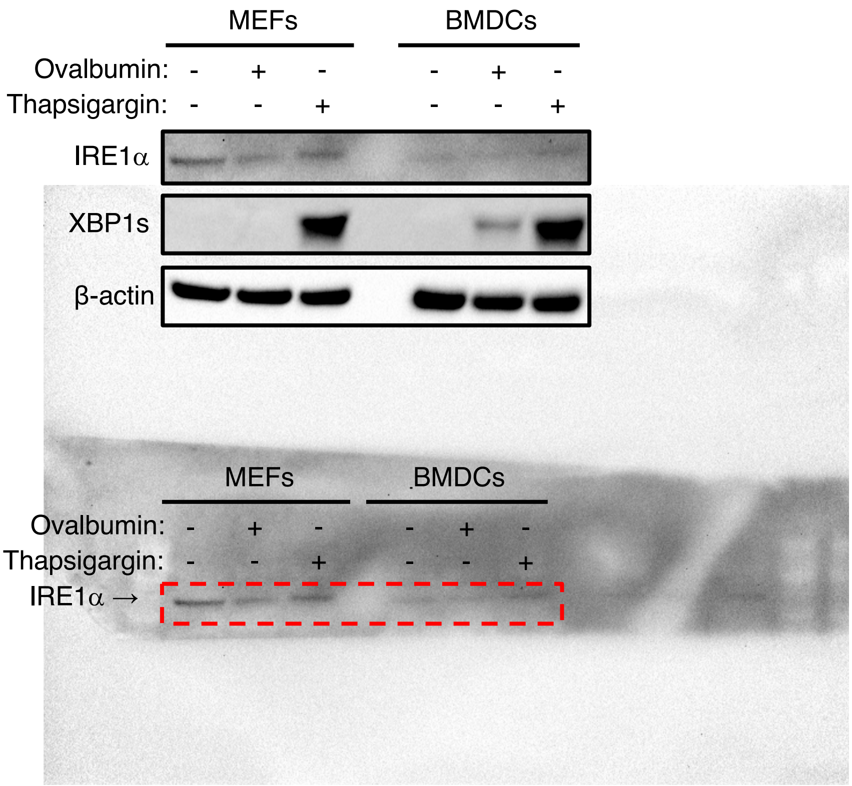

Figure S1. Antigen pulsing of BMDCs activates IRE1α. (G) MEFs and BMDCs were pulsed with ovalbumin or treated with Tg (100 nM) for 4 hr and analyzed by IB.
