# Supplementary material for: Antigen-derived peptides engage the ER stress sensor IRE1α to curb dendritic cell cross-presentation
Source: J Cell Biol. 2022 Apr 21;221(6):e202111068. doi: 10.1083/jcb.202111068 (PMC9036094; doi:10.1083/jcb.202111068)
Supplement: SourceData FS2 — contains original blots for Fig. S2. [file JCB_202111068_SourceDataFS2.pdf]

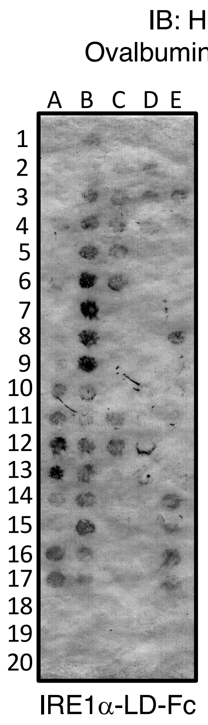

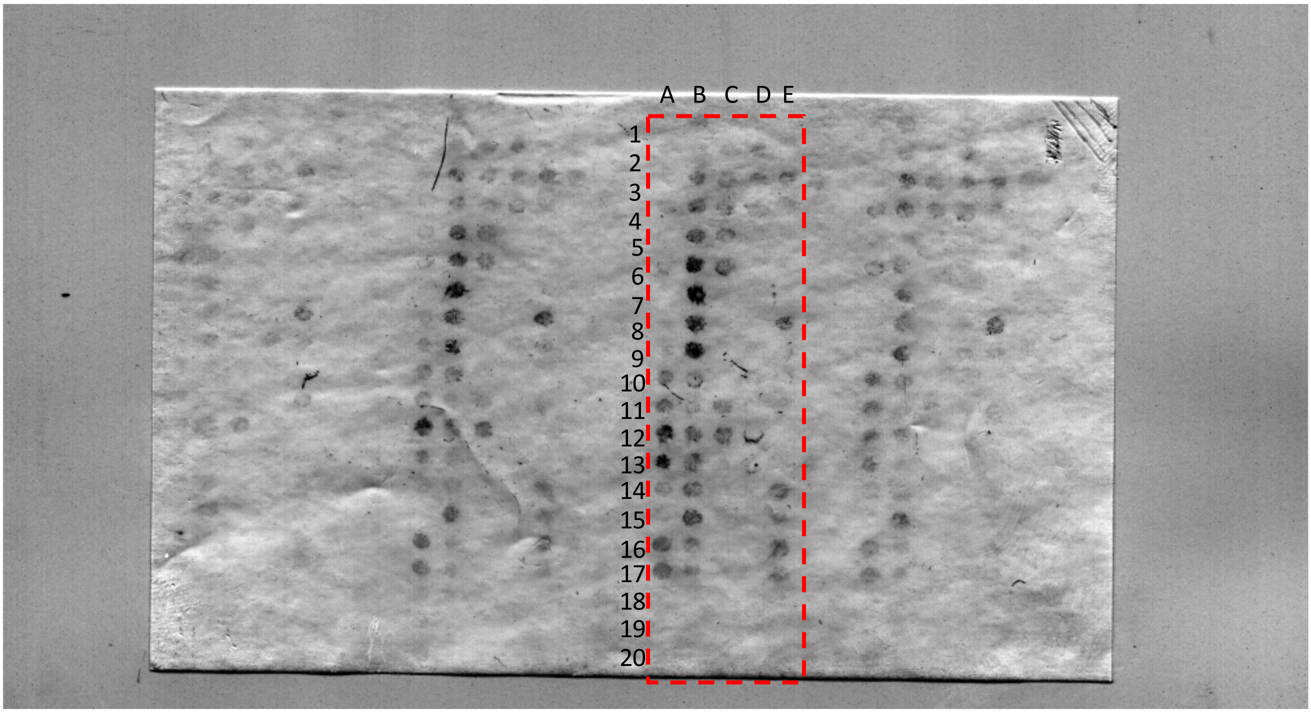

Figure S2. Antigen-derived peptides can directly engage IRE1α. (A) A tiled 18 aa-long peptide array spanning ovalbumin was incubated with IRE1α LD-Fc (500 nM) followed by IB analysis with an HRP-conjugated anti-human Fc antibody.

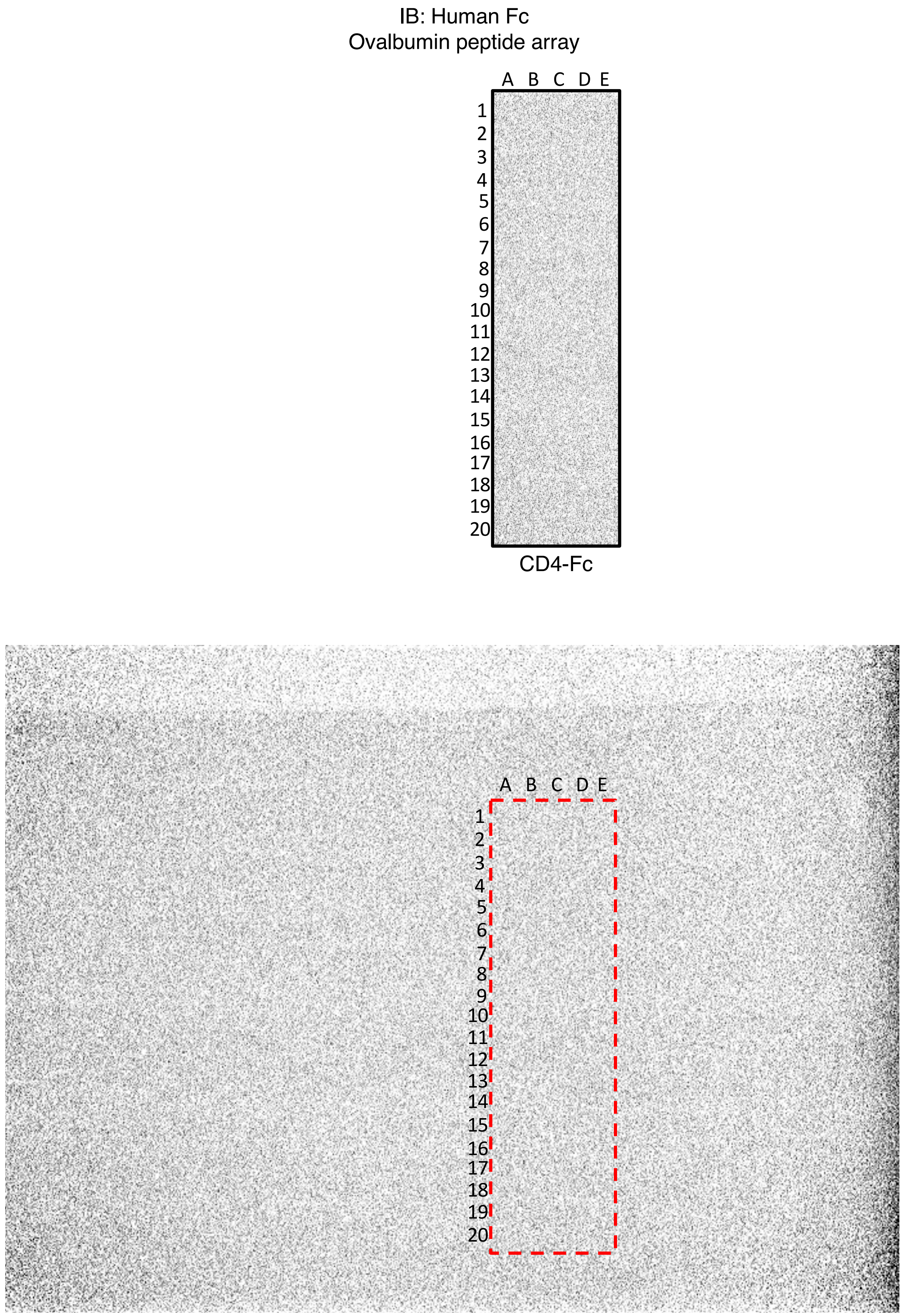

Figure S2. Antigen-derived peptides can directly engage IRE1α. (A) A tiled 18 aa-long peptide array spanning ovalbumin was incubated with IRE1α LD-Fc (500 nM) followed by IB analysis with an HRP-conjugated anti-human Fc antibody.
