# Supplementary material for: Antigen-derived peptides engage the ER stress sensor IRE1α to curb dendritic cell cross-presentation
Source: J Cell Biol. 2022 Apr 21;221(6):e202111068. doi: 10.1083/jcb.202111068 (PMC9036094; doi:10.1083/jcb.202111068)
Supplement: SourceData FS4 — contains original blots for Fig. S4. [file JCB_202111068_SourceDataFS4.pdf]

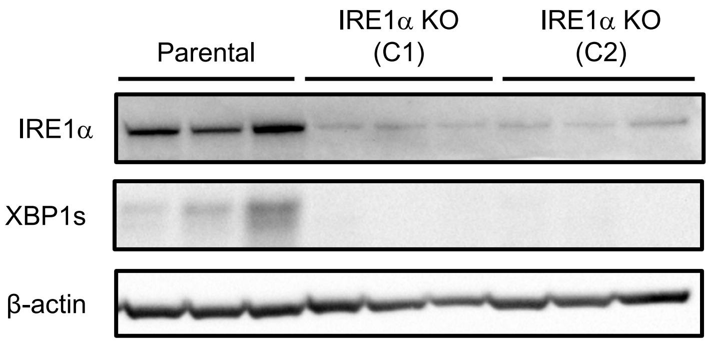

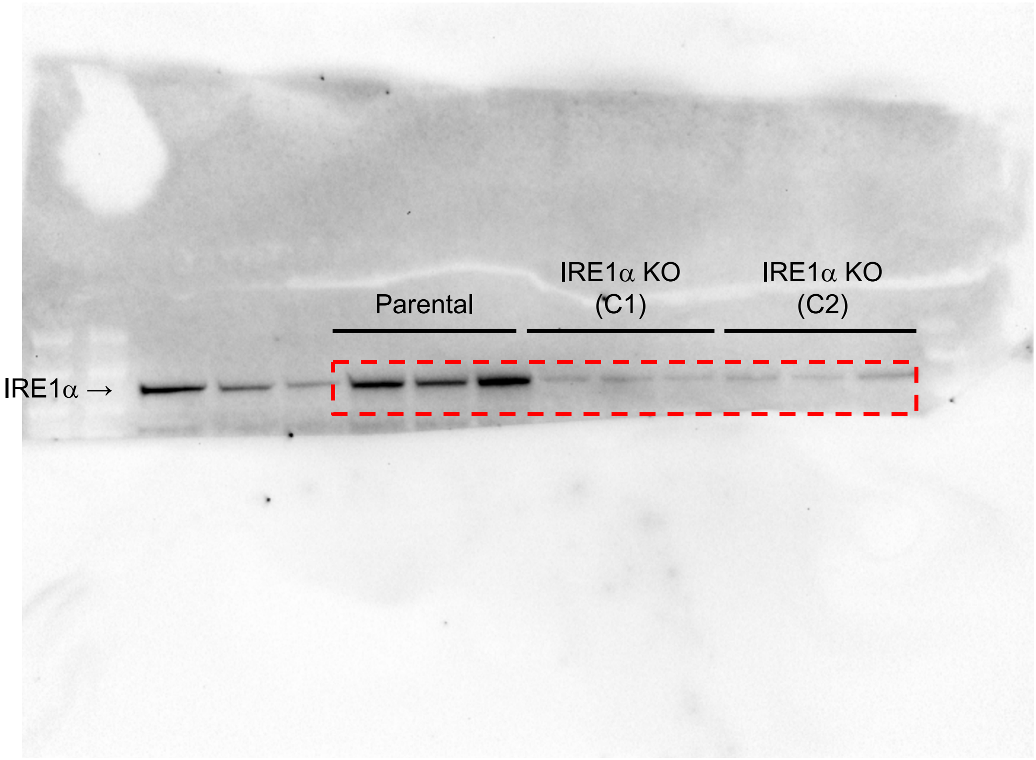

Figure S4. IRE1α inhibition attenuates CT26 tumor growth. (A, B) Animals were inoculated s.c. with parental or IRE1α KO CT26 cells and tumor growth was measured over 27 days. **(B)** IB analysis of IRE1α expression and activation.

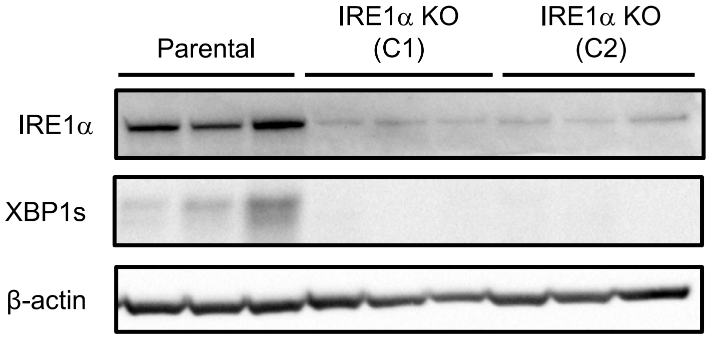

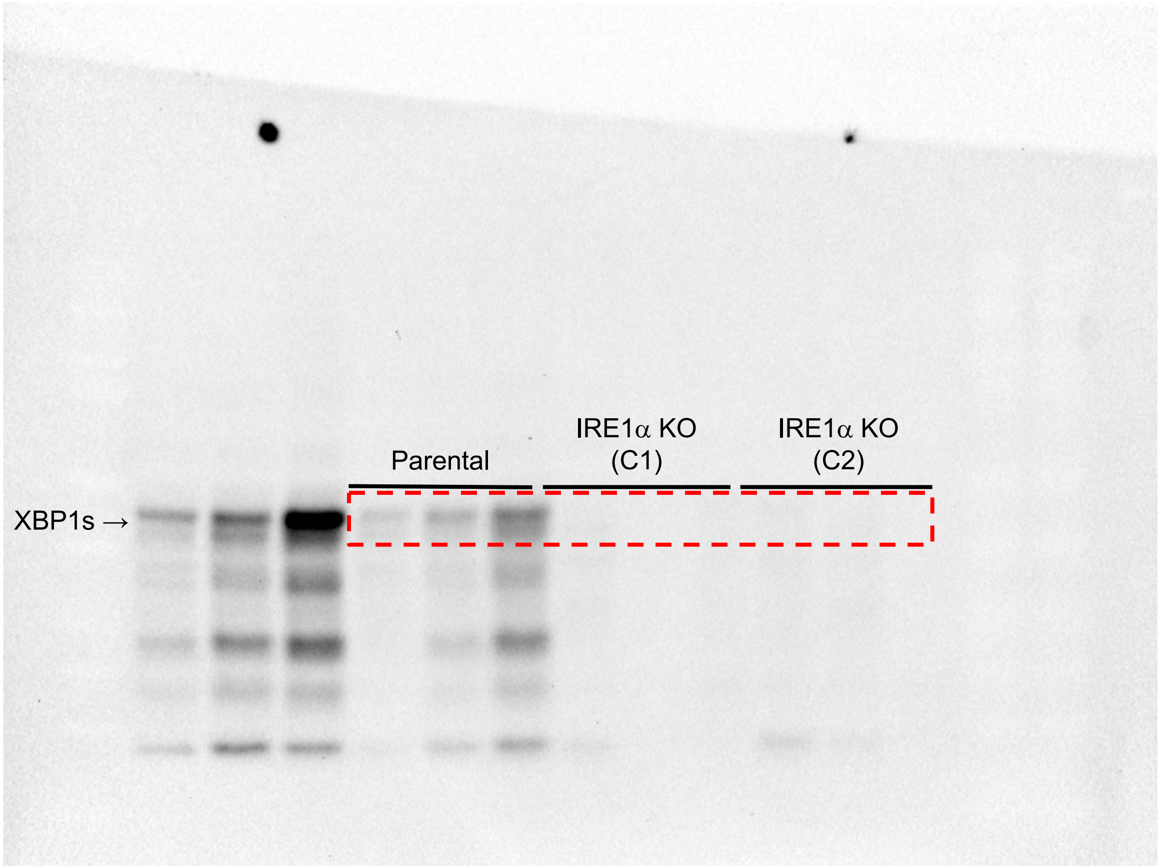

Figure S4. IRE1α inhibition attenuates CT26 tumor growth. (A, B) Animals were inoculated s.c. with parental or IRE1α KO CT26 cells and tumor growth was measured over 27 days. **(B)** IB analysis of IRE1α expression and activation.

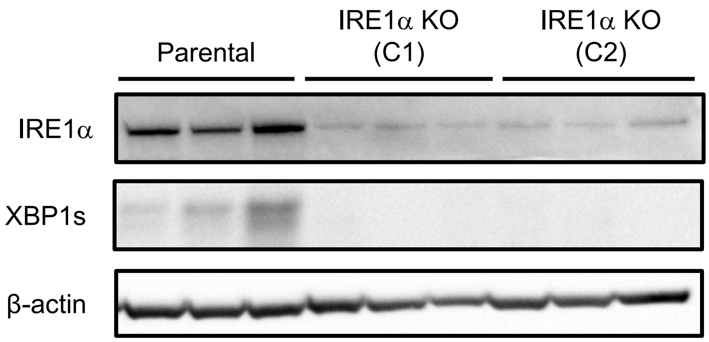

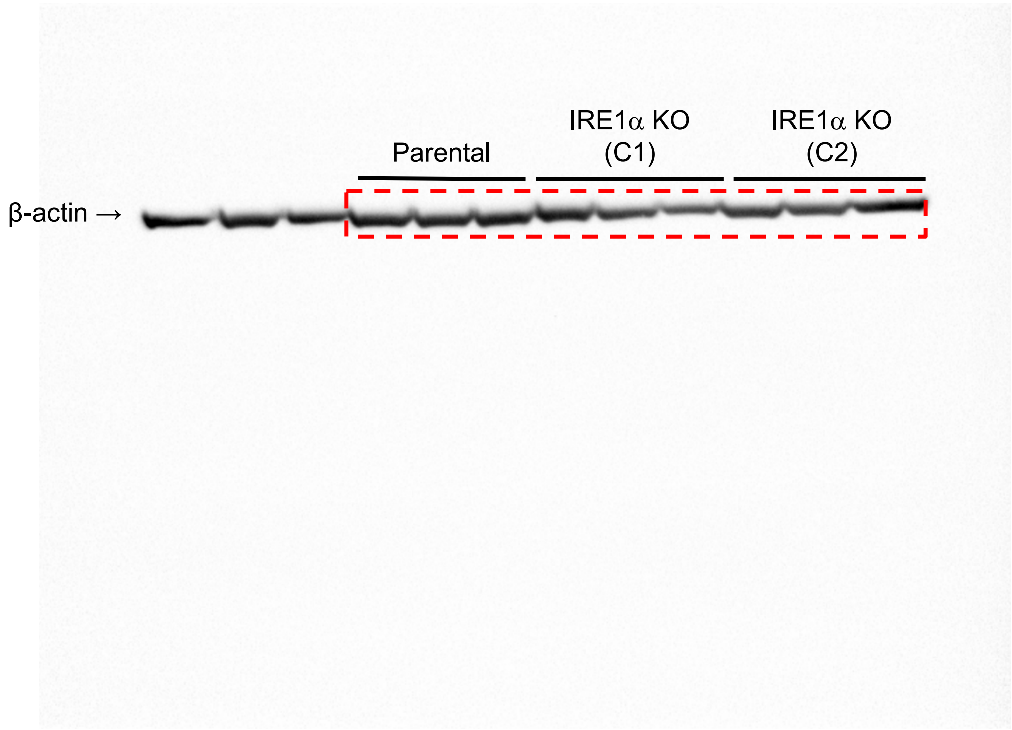

Figure S4. IRE1α inhibition attenuates CT26 tumor growth. (A, B) Animals were inoculated s.c. with parental or IRE1α KO CT26 cells and tumor growth was measured over 27 days. **(B)** IB analysis of IRE1α expression and activation.

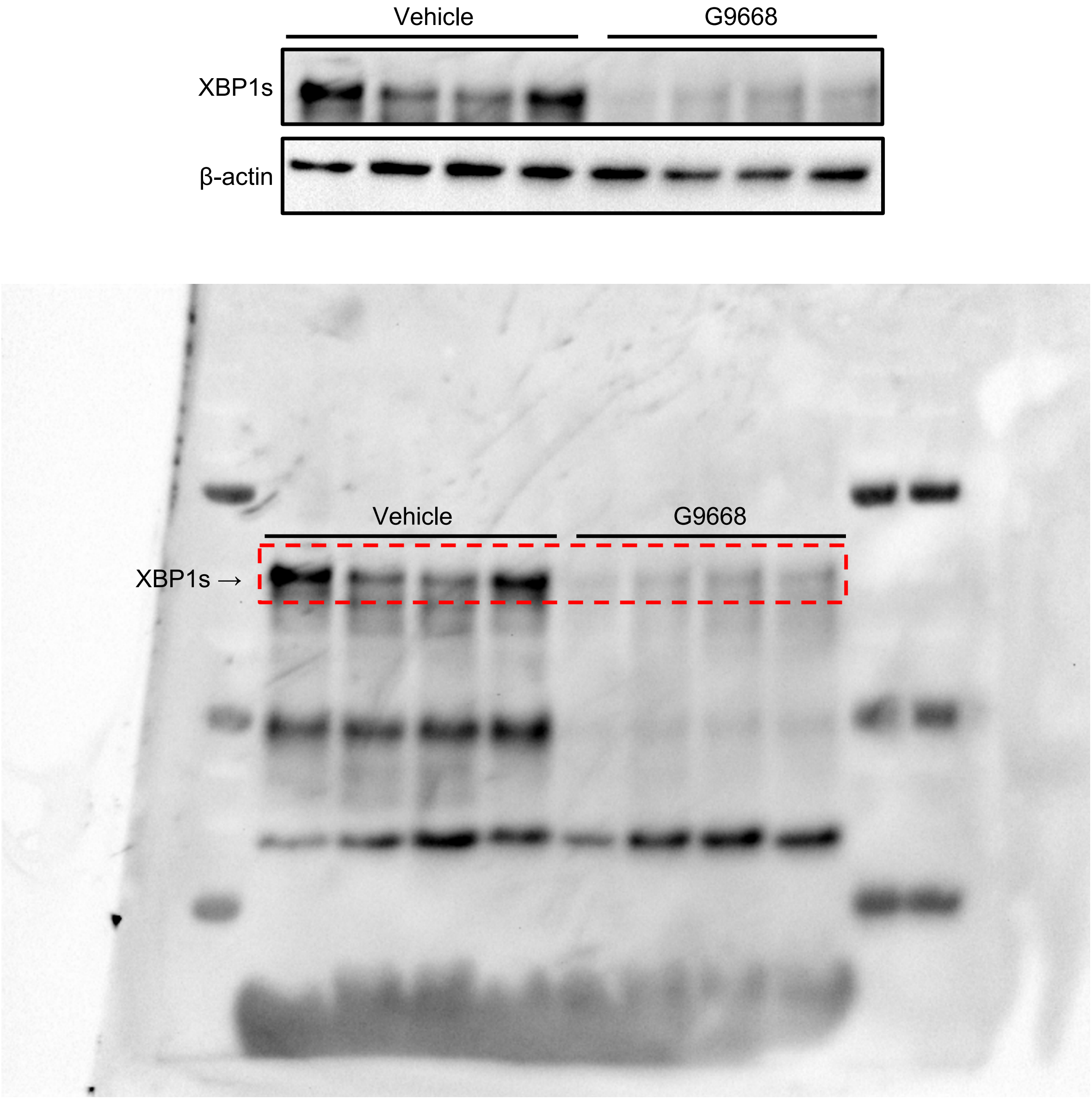

Figure S4. IRE1α inhibition attenuates CT26 tumor growth. (D) Mice were inoculated s.c. with CT26 cells, grouped out 7 days afterwards and treated with vehicle or G9668 (250 mg/kg, BID). IB analysis of total tumor lysates is depicted.

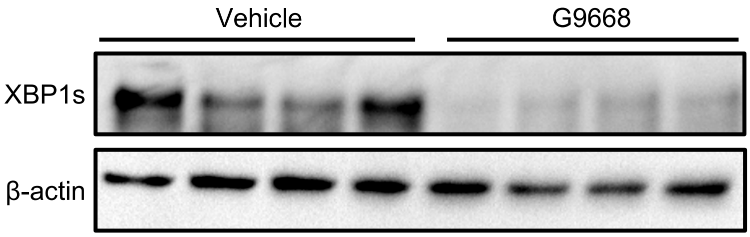

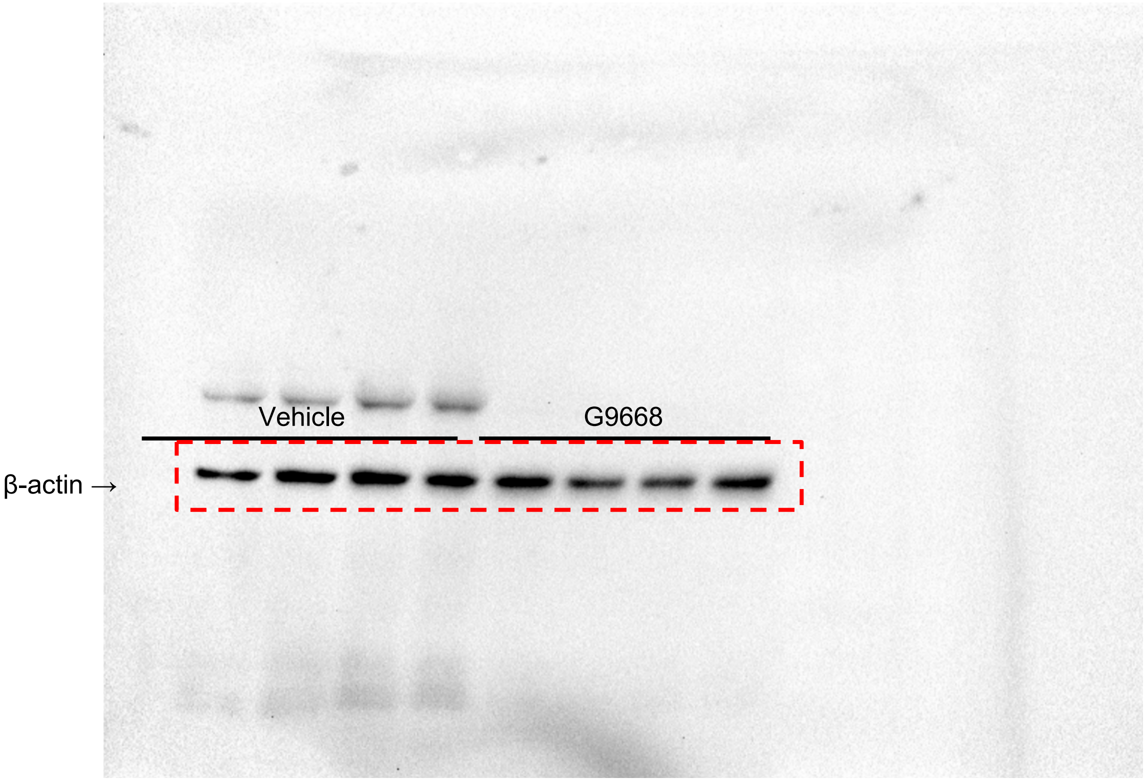

Figure S4. IRE1α inhibition attenuates CT26 tumor growth. (D) Mice were inoculated s.c. with CT26 cells, grouped out 7 days afterwards and treated with vehicle or G9668 (250 mg/kg, BID). IB analysis of total tumor lysates is depicted.
