# Supplementary material for: Antigen-derived peptides engage the ER stress sensor IRE1α to curb dendritic cell cross-presentation
Source: J Cell Biol. 2022 Apr 21;221(6):e202111068. doi: 10.1083/jcb.202111068 (PMC9036094; doi:10.1083/jcb.202111068)
Supplement: SourceData FS5 — contains original blots for Fig. S5. [file JCB_202111068_SourceDataFS5.pdf]

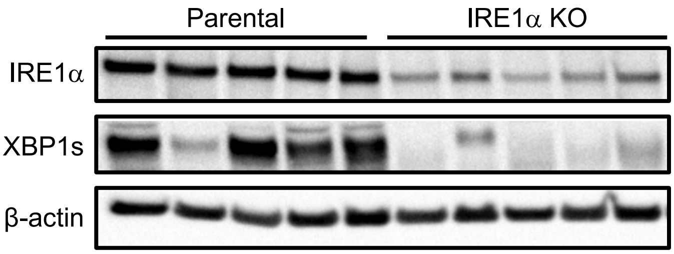

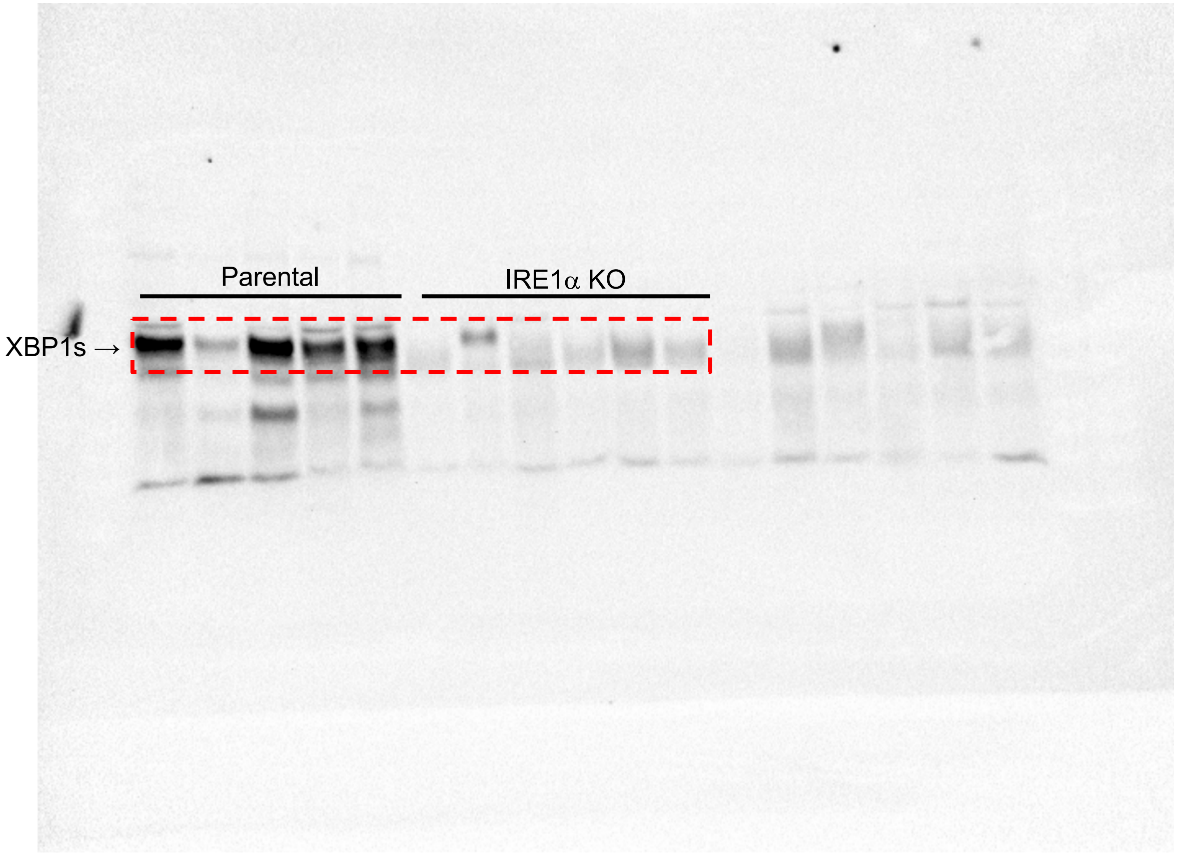

Figure S5. IRE1α inhibition attenuates 4T1 tumor growth. (B) Animals were inoculated s.c. with parental or IRE1α KO 4T1 cells and tumor growth was monitored over 25 days, IB analysis of total tumor lysates **(B)** presented.

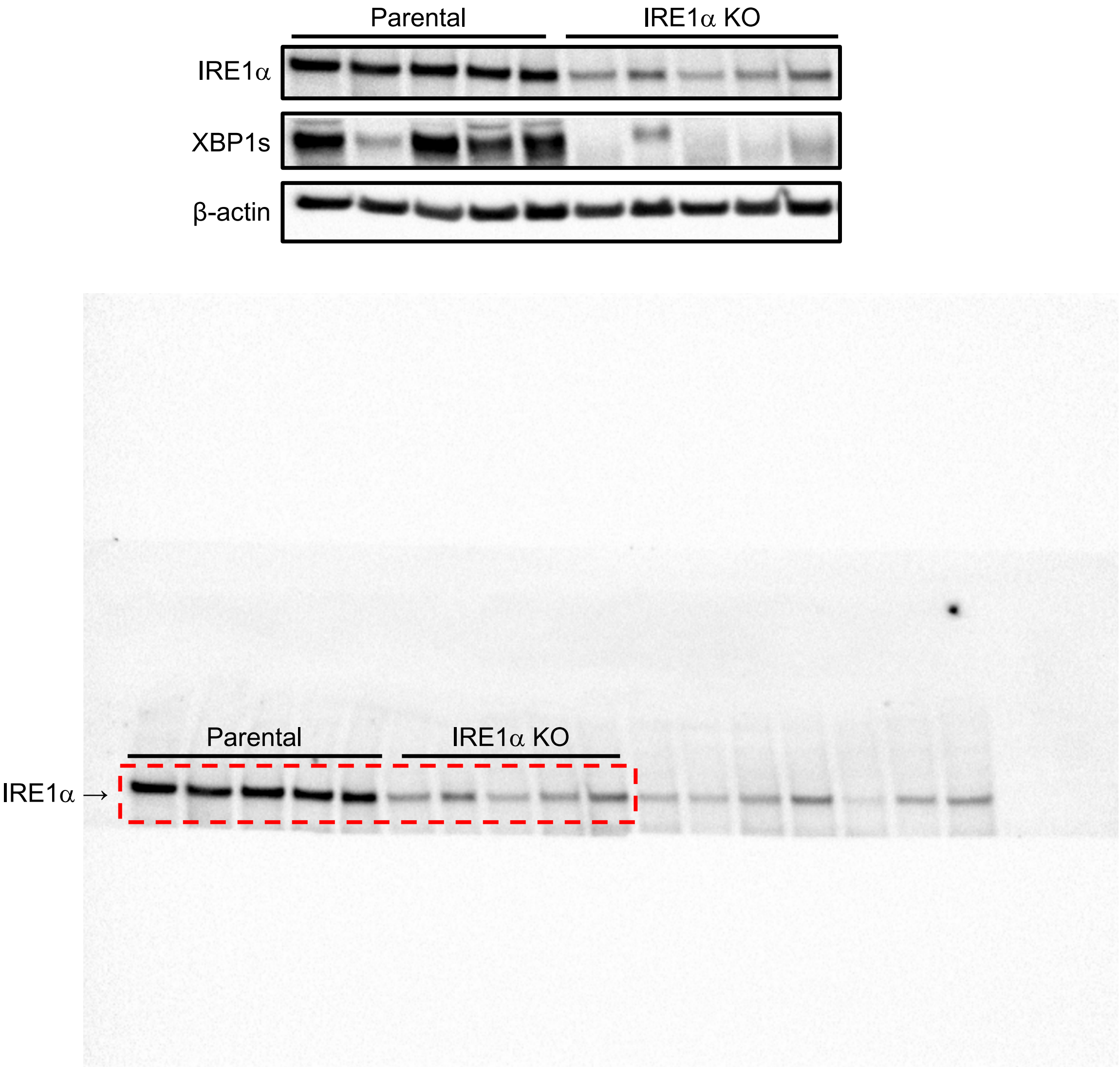

Figure S5. IRE1α inhibition attenuates 4T1 tumor growth. (B) Animals were inoculated s.c. with parental or IRE1α KO 4T1 cells and tumor growth was monitored over 25 days, IB analysis of total tumor lysates **(B)** presented.

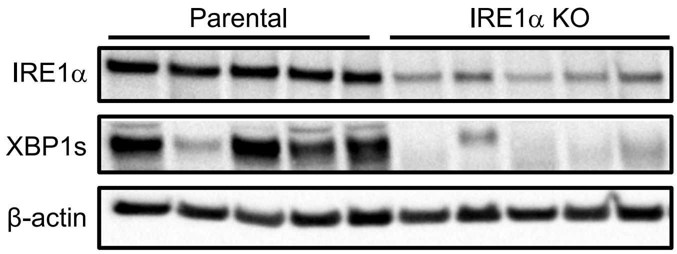

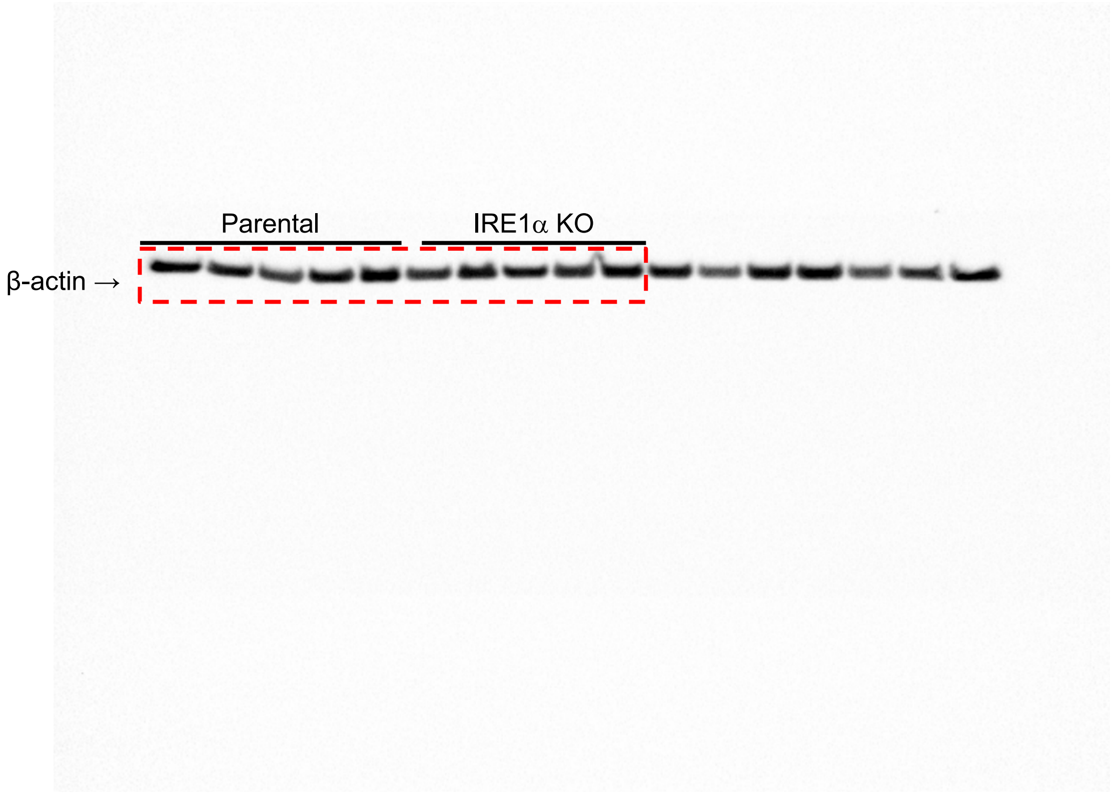

Figure S5. IRE1α inhibition attenuates 4T1 tumor growth. (B) Animals were inoculated s.c. with parental or IRE1α KO 4T1 cells and tumor growth was monitored over 25 days, IB analysis of total tumor lysates **(B)** presented.

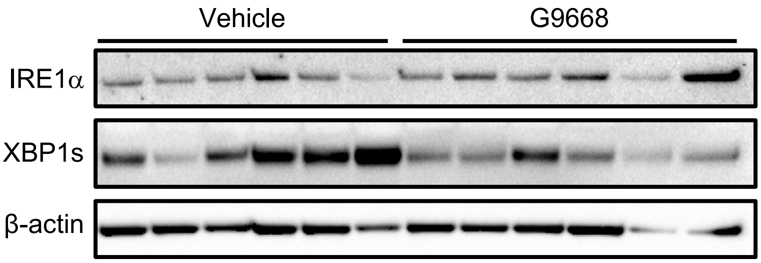

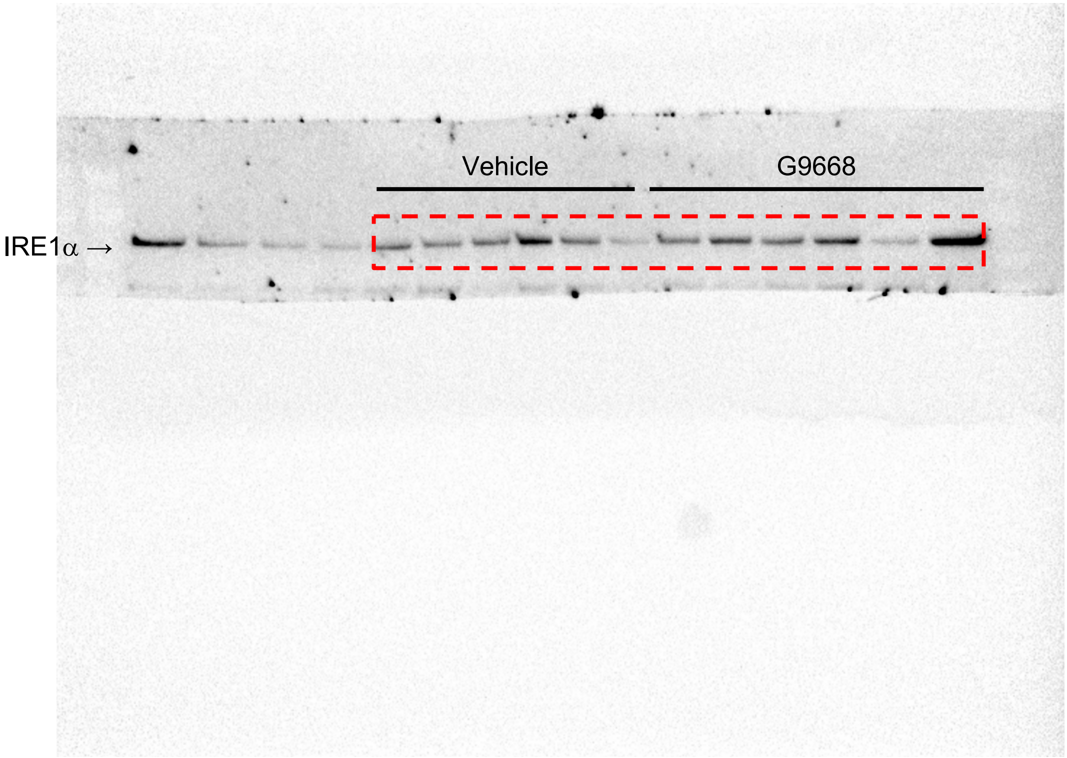

Figure S5. IRE1α inhibition attenuates 4T1 tumor growth. (D) Mice were inoculated s.c. with 4T1 cells, grouped out 7 days afterwards and treated with vehicle or G9668 (250 mg/kg, BID). IRE1α expression and activation were analyzed by IB.

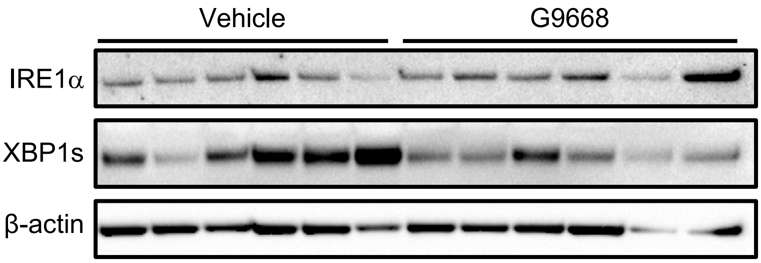

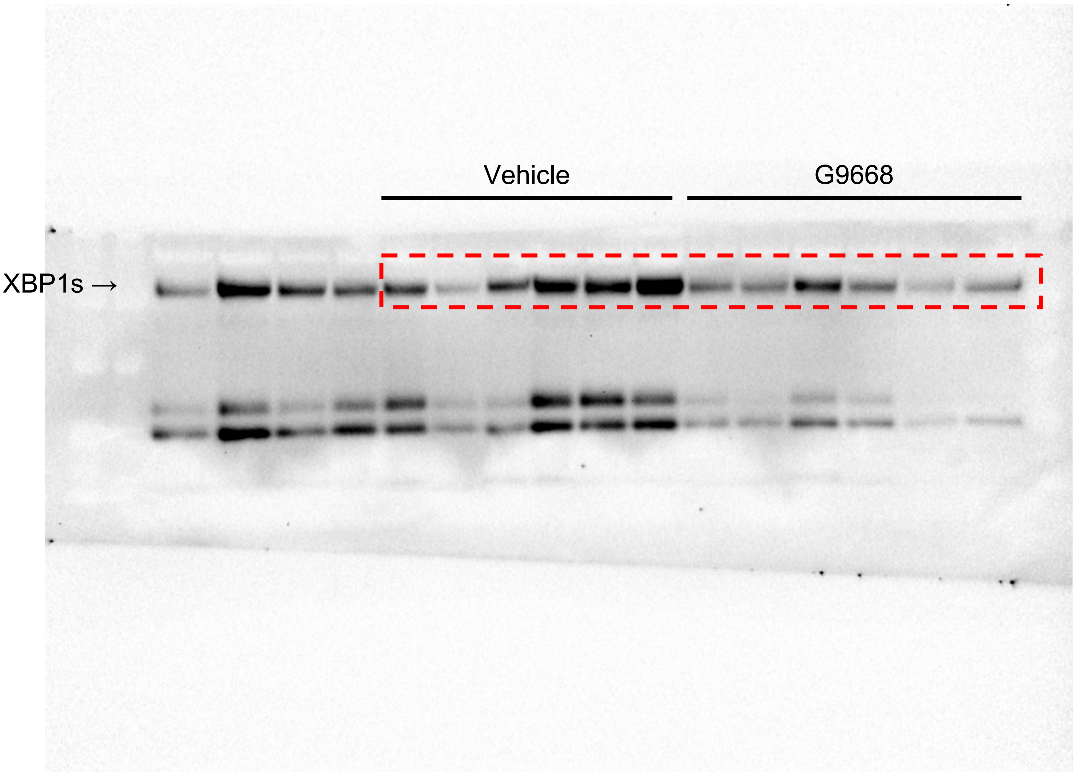

Figure S5. IRE1α inhibition attenuates 4T1 tumor growth. (D) Mice were inoculated s.c. with 4T1 cells, grouped out 7 days afterwards and treated with vehicle or G9668 (250 mg/kg, BID). IRE1α expression and activation were analyzed by IB.

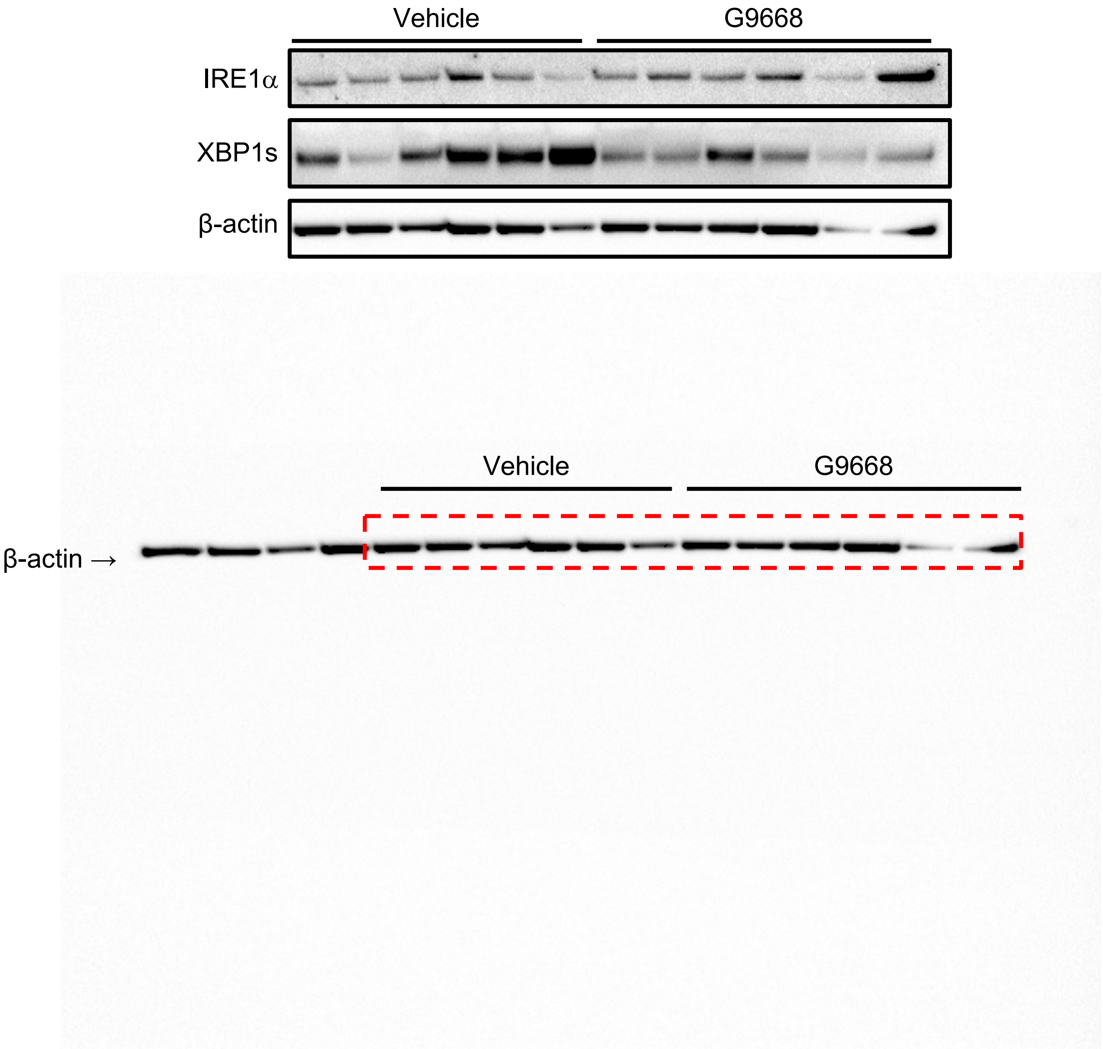

Figure S5. IRE1α inhibition attenuates 4T1 tumor growth. (D) Mice were inoculated s.c. with 4T1 cells, grouped out 7 days afterwards and treated with vehicle or G9668 (250 mg/kg, BID). IRE1α expression and activation were analyzed by IB.
