# Supplementary material for: Antigen-derived peptides engage the ER stress sensor IRE1α to curb dendritic cell cross-presentation
Source: J Cell Biol. 2022 Apr 21;221(6):e202111068. doi: 10.1083/jcb.202111068 (PMC9036094; doi:10.1083/jcb.202111068)
Supplement: SourceData FS6 — contains original blots for Fig. S6. [file JCB_202111068_SourceDataFS6.pdf]

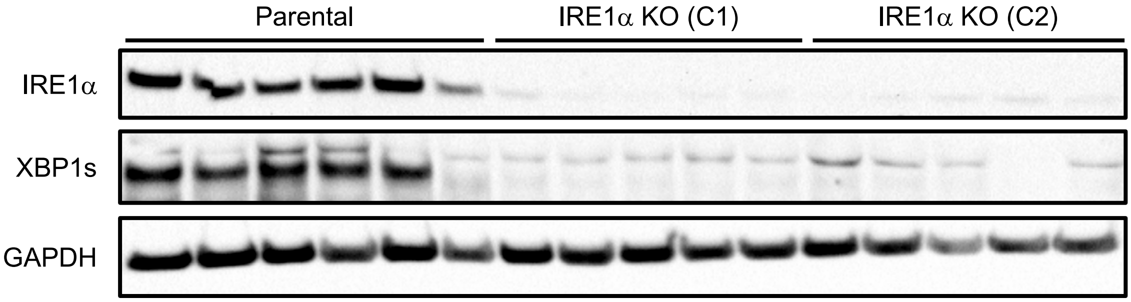

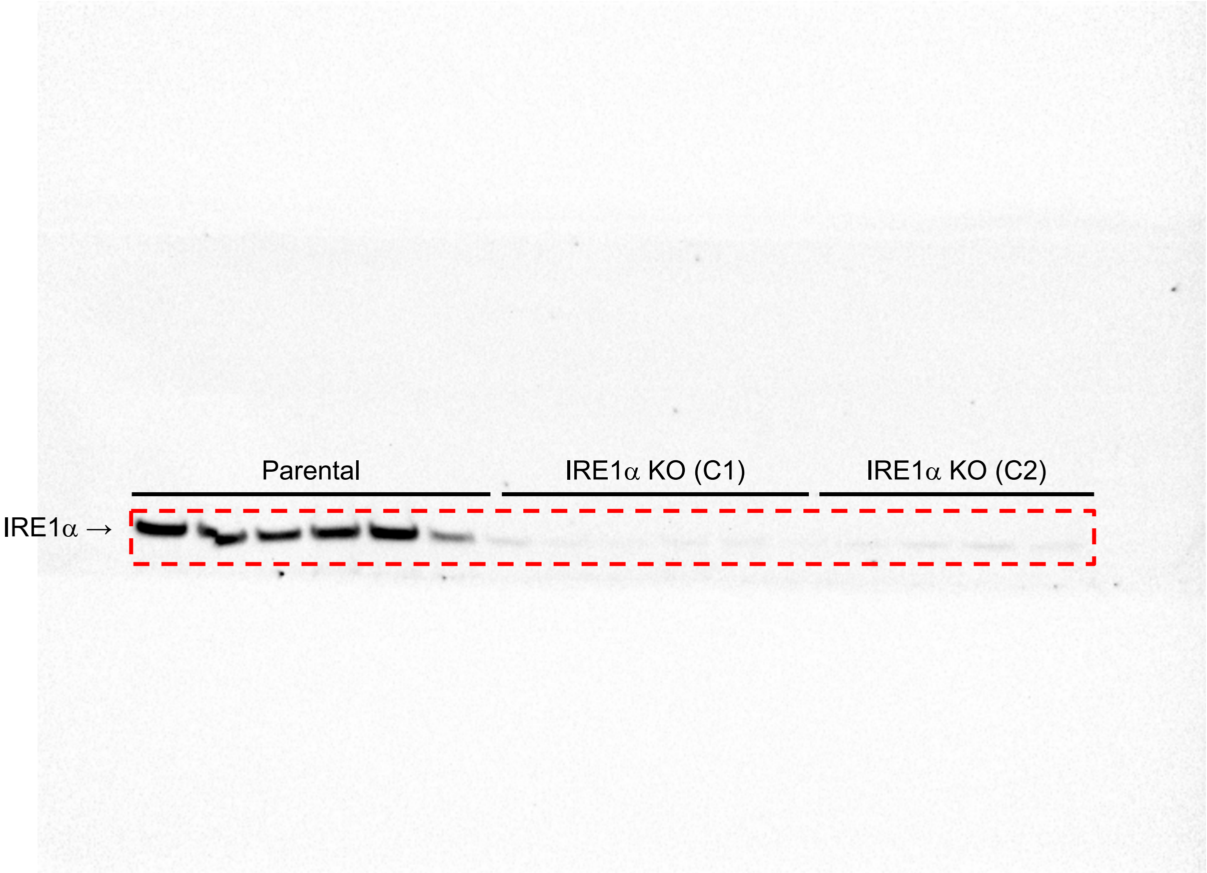

Figure S6. IRE1α inhibition attenuates EMT6 tumor growth and synergizes with anti-PD-L1 antibody. (B) Mice were inoculated orthotopically with WT or IRE1α KO EMT6 cells and tumor growth was measured over 24 days. IB analysis of total tumor lysates **(B)** presented.

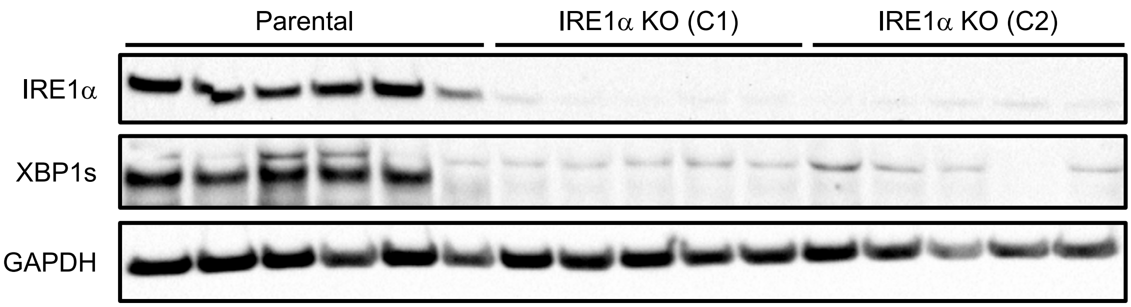

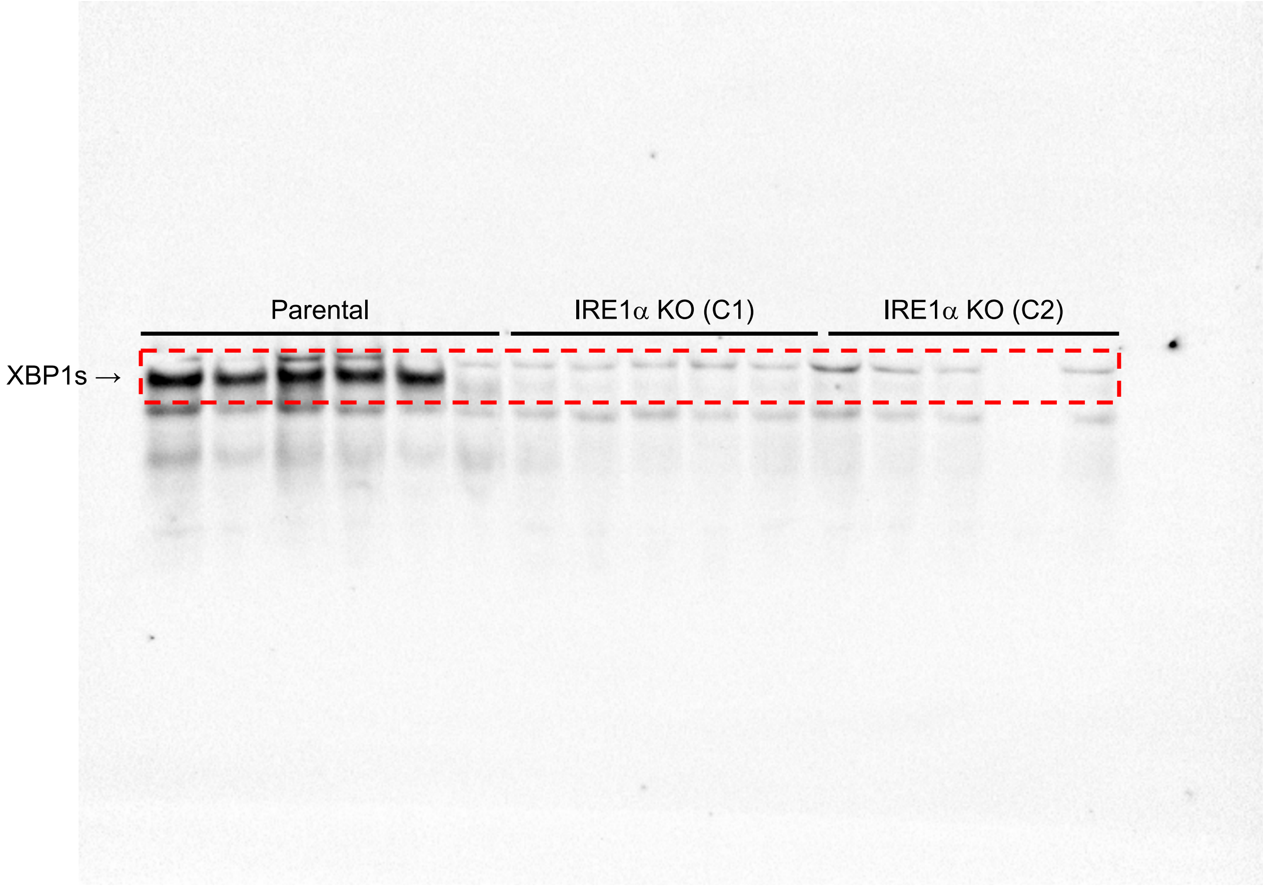

Figure S6. IRE1α inhibition attenuates EMT6 tumor growth and synergizes with anti-PD-L1 antibody. (B) Mice were inoculated orthotopically with WT or IRE1α KO EMT6 cells and tumor growth was measured over 24 days. IB analysis of total tumor lysates **(B)** presented.

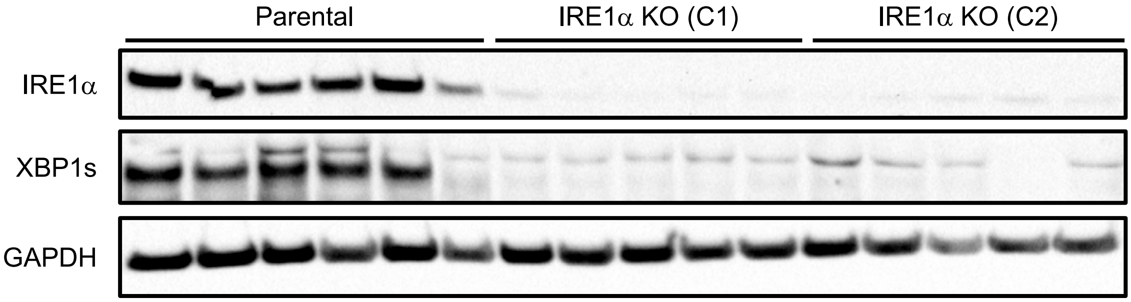

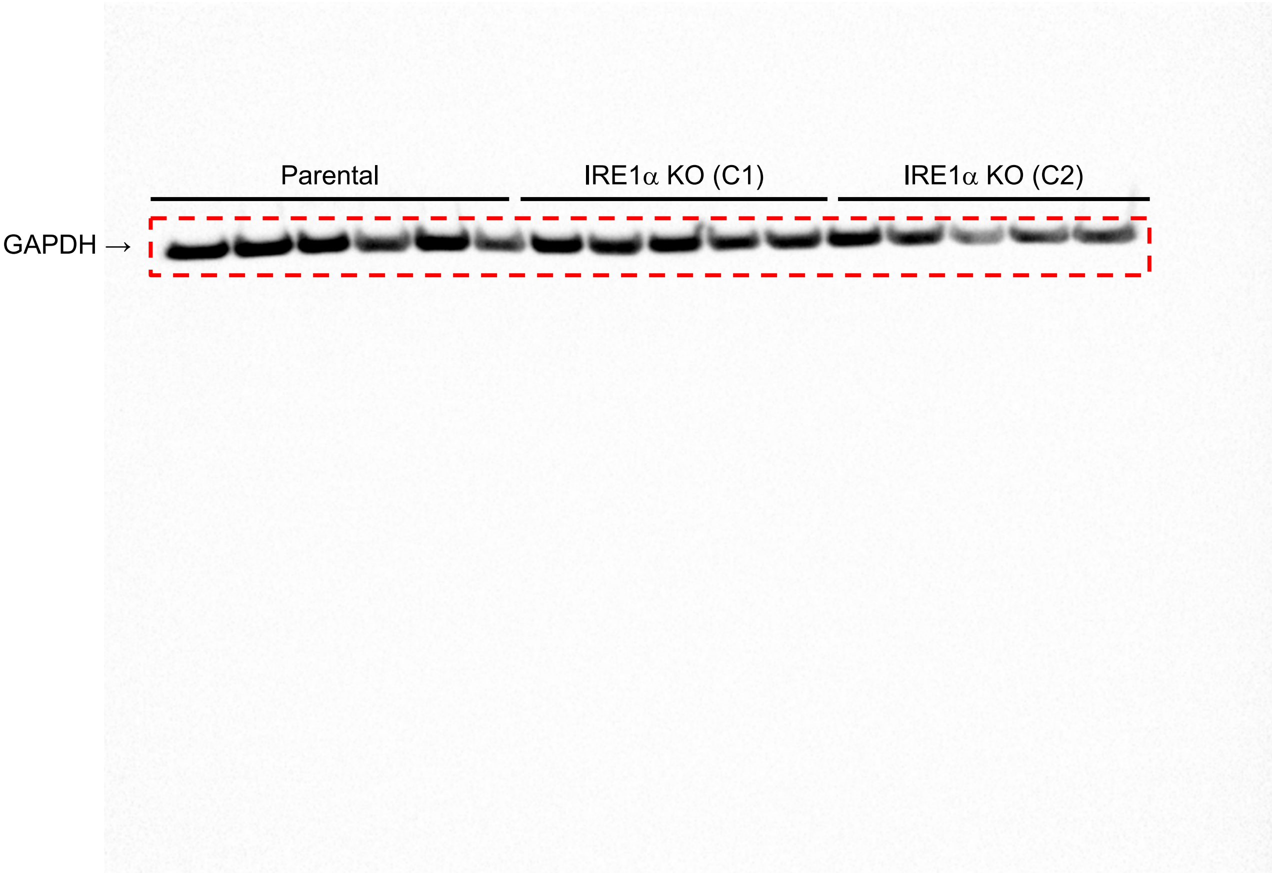

Figure S6. IRE1α inhibition attenuates EMT6 tumor growth and synergizes with anti-PD-L1 antibody. (B) Mice were inoculated orthotopically with WT or IRE1α KO EMT6 cells and tumor growth was measured over 24 days. IB analysis of total tumor lysates **(B)** presented.

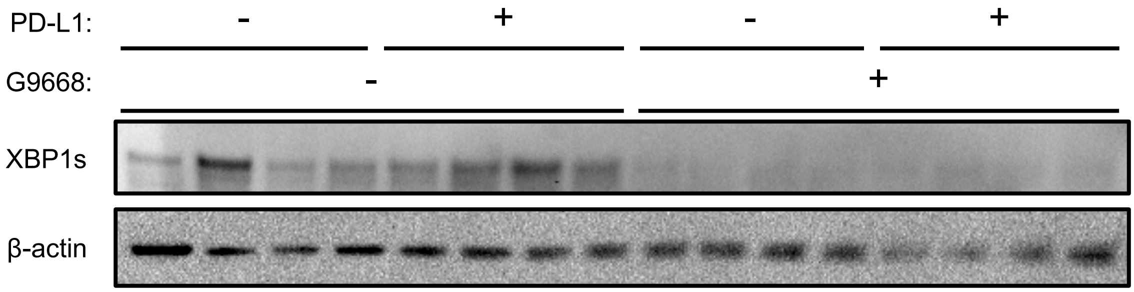

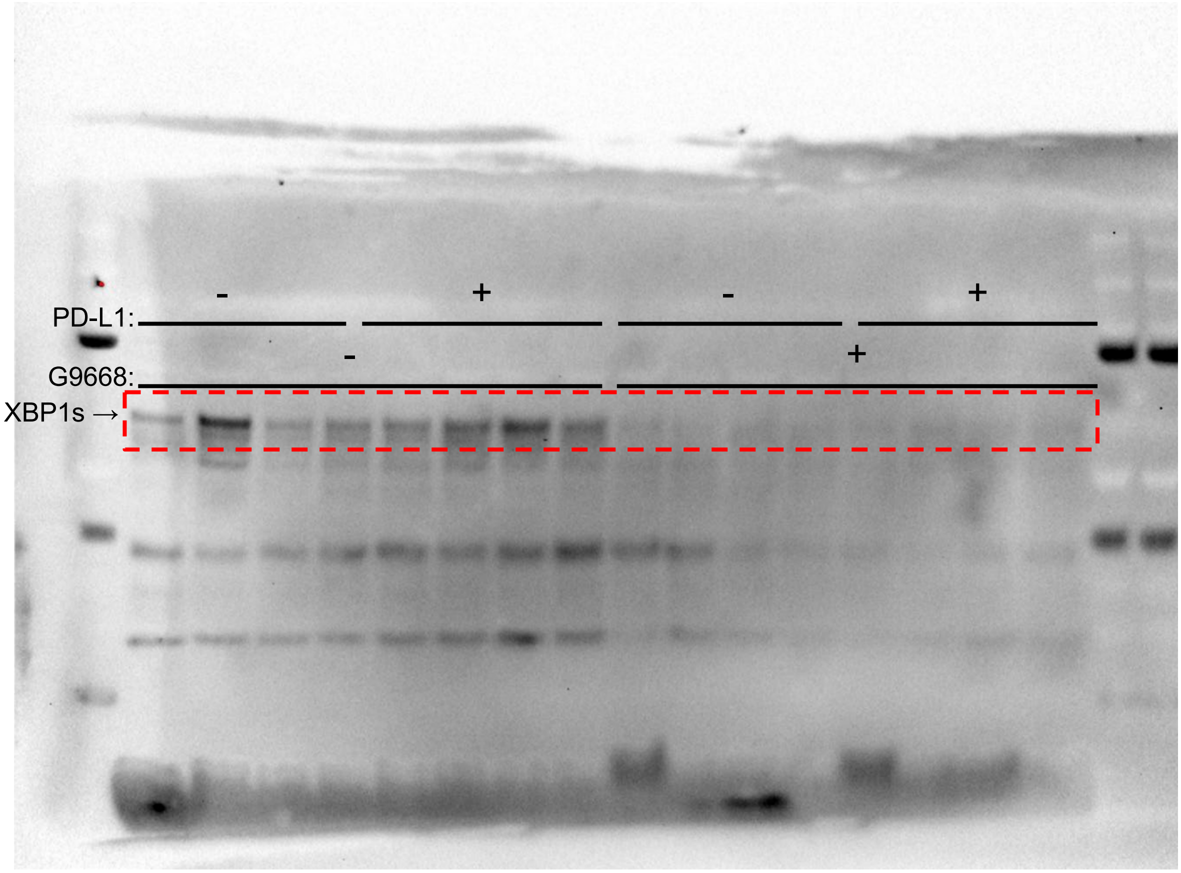

Figure S6. IRE1α inhibition attenuates EMT6 tumor growth and synergizes with anti-PD-L1 antibody. (D) Mice were inoculated with WT or IRE1α KO EMT6 cells, grouped out 7 days afterwards and treated with vehicle, G9668 (250 mg/kg, BID), anti-PD-L1 antibody (10 mg/kg at first dose, 5 mg/kg BIW thereafter), or the combination. (**D)** IRE1α activation was analyzed by IB.

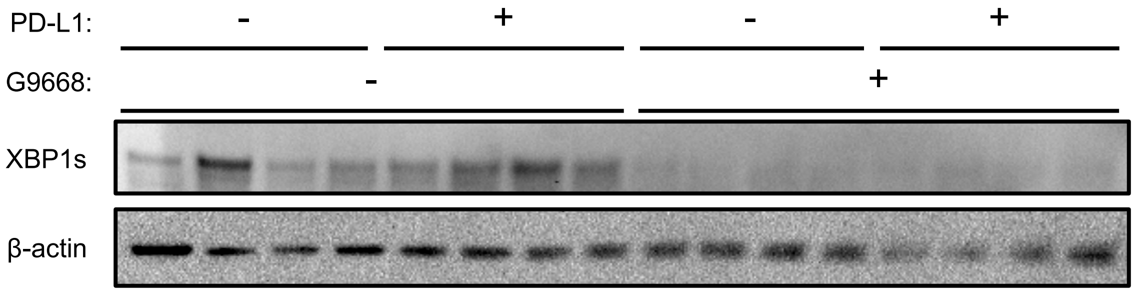

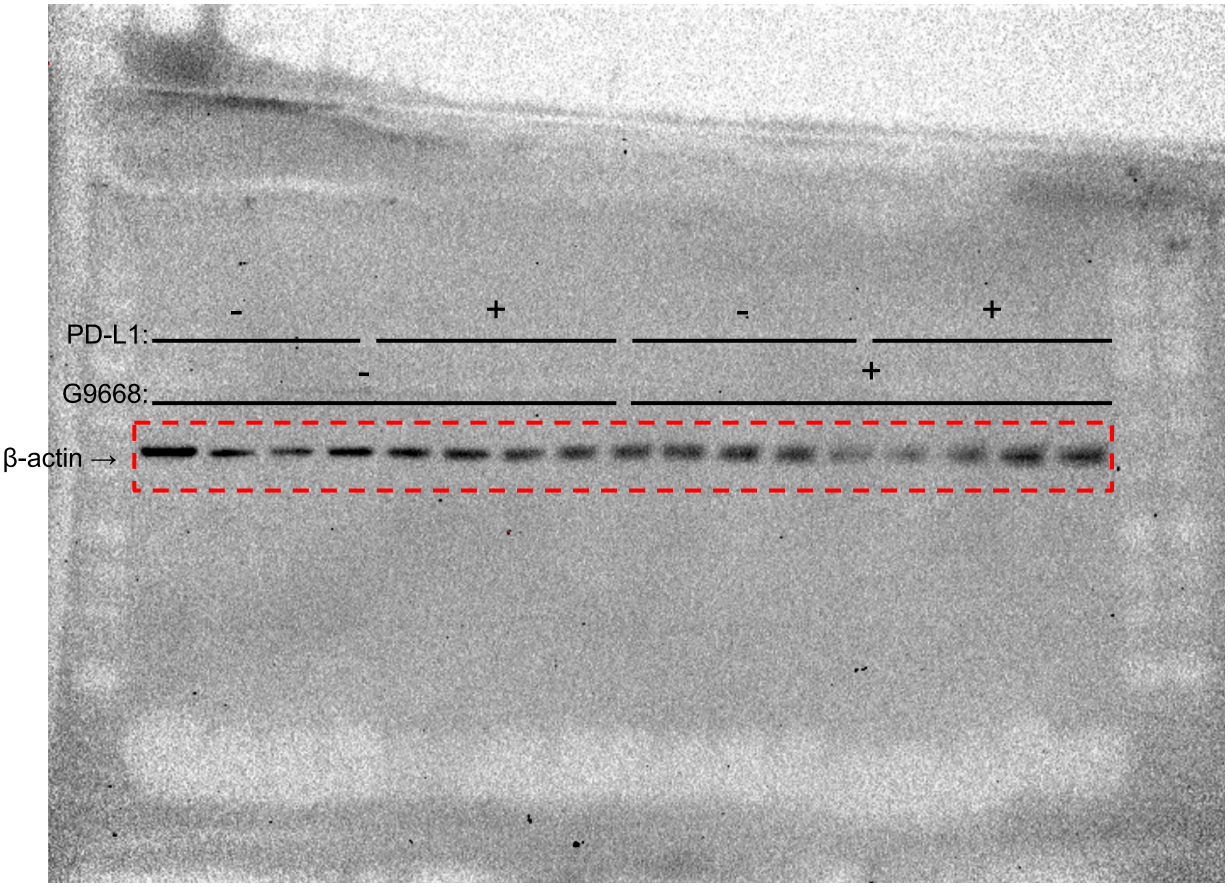

Figure S6. IRE1α inhibition attenuates EMT6 tumor growth and synergizes with anti-PD-L1 antibody. (D) Mice were inoculated with WT or IRE1α KO EMT6 cells, grouped out 7 days afterwards and treated with vehicle, G9668 (250 mg/kg, BID), anti-PD-L1 antibody (10 mg/kg at first dose, 5 mg/kg BIW thereafter), or the combination. (**D**) IRE1α activation was analyzed by IB.
